# 2‐Chloro‐5‐(trifluoromethyl)aniline‐Derived Schiff Base Palladium(II) Complexes: From Molecular Design to Acute Toxicity Assessment

**DOI:** 10.1002/open.70315

**Published:** 2026-09-30

**Authors:** Andreea Elena Ştefan, Cristina Elena Dinu‐Pîrvu, Sergiu Shova, Ştefania Felicia Bărbuceanu, Teodora Venera Apostol, Diana Carolina Ilieş, Mihaela Badea, Mihaela Dinu, Robert Viorel Ancuceanu, Doina Drăgănescu, Elena Pahonţu

**Affiliations:** ^1^ General and Inorganic Chemistry Department Faculty of Pharmacy “Carol Davila” University of Medicine and Pharmacy Bucharest Romania; ^2^ Physical and Colloidal Chemistry Department Faculty of Pharmacy “Carol Davila” University of Medicine and Pharmacy Bucharest Romania; ^3^ Institute of Macromolecular Chemistry “Petru Poni” Iasi Romania; ^4^ Organic Chemistry Department Faculty of Pharmacy “Carol Davila” University of Medicine and Pharmacy Bucharest Romania; ^5^ Inorganic Chemistry Department Faculty of Chemistry University of Bucharest Bucharest Romania; ^6^ Pharmaceutical Botany and Cell Biology Department Faculty of Pharmacy “Carol Davila” University of Medicine and Pharmacy Bucharest Romania; ^7^ Pharmaceutical Physics Department Faculty of Pharmacy “Carol Davila” University of Medicine and Pharmacy Bucharest Romania

**Keywords:** artemia toxicity, halogenated ligands, Pd(II) Schiff base complexes, phytotoxicity, X‐ray crystallography

## Abstract

Four Schiff bases newly synthesized from 2‐chloro‐5‐(trifluoromethyl)aniline served as ligands for the preparation of four palladium(II) complexes. Characterization of all compounds was performed using the following physicochemical techniques: elemental analysis, IR, UV–vis, ^1^H NMR, ^13^C NMR, mass spectrometry, thermal analysis, molar conductivity, and magnetic susceptibility. In addition, crystals of the ligands **HL** ^**1**^–**HL** ^**4**^ and the complexes **2** and **4** were obtained, enabling their structures to be confirmed by single‐crystal X‐ray diffraction. Crystallography showed that ligands form molecular crystals with one molecule per unit cell, except **HL** ^**1**^, which contains two chemically identical molecules; **HL** ^**1**^, **HL** ^**2**^, and **HL** ^**4**^ are centrosymmetric, and **HL** ^**3**^ crystallizes as a pure enantiomer. Complex **4** is mononuclear, coordinated by two deprotonated ligands through an N_2_O_2_ donor set, while complex **2** is dinuclear, with each palladium(II) coordinated via an O_3_N set by one bidentate anionic ligand and two bidentate‐bridging acetate anions. Phytotoxicity and animal toxicity were assessed using *Triticum aestivum* L. and *Artemia franciscana* Kellogg, respectively. Complexes were slightly more inhibitory than ligands on *Triticum*, but overall effects were weak, with root growth exceeding that of the negative control at all tested concentrations. Only complexes **2** and **4** exhibited dose‐dependent toxicity in *Artemia*, with LC_50_ values of 389.8 and 48.7 µM.

## Graphical abstract

Four new Schiff bases derived from 2‐chloro‐5‐(trifluoromethyl)aniline and their corresponding palladium(II) complexes were obtained and analyzed by multiple spectroscopic techniques. Single‐crystal X‐ray diffraction elucidated the structures of all the ligands and complexes **2** and **4**. Their toxicity was assessed on *Triticum aestivum* L. and *Artemia franciscana* Kellogg species.

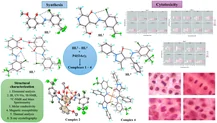

## 1 Introduction

For a long time, platinum compounds have been a key component in cancer treatment. However, their severe toxicity and the increasing resistance of tumor cells to these drugs have determined the scientific community to explore safer alternatives [1]. In the past decade, palladium complexes have gained attention as a promising alternative to platinum chemotherapeutics [2].

Palladium is very similar to platinum regarding its physical and chemical properties. Moreover, the studies have shown that palladium‐based drugs are less toxic to healthy tissues compared to platinum‐based ones. This occurs because sulfhydryl groups of amino acids cannot substitute the strongly bound ligands of palladium(II) during interactions with cellular proteins [3, 4]. However, research on palladium(II) complexes has extended beyond their antitumor activity, with studies showing that they may also exhibit antioxidant [5, 6], antiviral [7], antimalaric [8], antibacterial [9], antifungal [10, 11], antidiabetic [12], anticoagulant [13], and antiinflammatory [14] properties.

Existing literature highlights the importance of choosing appropriate ligands, as they significantly influence a complex's reactivity, lipophilicity, oxidation state, and substitutional stability [15]. In this context, Schiff bases, which exhibit distinctive properties due to the imine group, have been the subject of significant research. The presence of donor atoms enables them to act as ligands and allows the formation of complexes with various coordination geometries and coordination numbers, enhancing both stability and selectivity [16].

Additionally, numerous studies have shown that the nature of the carbonyl compounds, like vanillin, pyrazole, or pyridine derivatives, used in Schiff base synthesis greatly affect the biological behavior of the resulting compounds. As a consequence, those Schiff bases exhibit a variety of pharmacological activities, including anticancer [17, 18, 19], antibacterial [20, 21], antifungal [22, 23], antipyretic [24], antioxidant [25, 26], and anti‐inflammatory [27, 28].

Besides the wide range of applications in the medical field, Schiff bases also have applications in nonmedical areas such as catalysis [29], fluorescence, and chemosensing [30, 31], as pigments and dyes [32, 33], and as polymer stabilizers [34].

Another key element in optimizing the biological activity of drugs is the presence of fluorine. Incorporating fluorine atoms into a molecule can increase its solubility, lipophilicity, and membrane permeability, leading to enhanced pharmacological activity [35]. Fluorination can also change a molecule's acidity and basicity, significantly affecting its pharmacodynamic and pharmacokinetic properties [36]. Additionally, the position of the fluorine atom on an aromatic ring can influence electron distribution and, consequently, how the molecule interacts with its biological targets [23, 37, 38].

As fluorine substitution improves biological efficacy and chemical and metabolic stability, it has been widely explored in drug development. Therefore, fluorinated Schiff bases have gained attention in medicinal chemistry [39].

Recent studies have focused on the synthesis of fluorine‐containing Schiff bases and the evaluation of their biological activities. For example, a Schiff base derived from 4‐fluorobenzaldehyde exhibited superior antibacterial activity against *S. aureus*, *B. cereus*, *Rhizopus,* and *E. coli* compared with penicillin [40]. Another Schiff base, derived from 3,5‐difluorine‐2‐hydroxy‐benzaldehyde, exhibited antifungal effects against *Cryptococcus* spp. [23]. Additionally, six Schiff bases obtained from 3‐fluorosalicylaldehyde showed antibacterial activity against *K. pneumoniae*, *S. typhi*, *S. aureus*, *B. subtilis*, *P. aeruginosa*, *E. coli*, and *P. chrysogenum*, as well as antifungal activity against *C. albicans*, *A. niger,* and *A. flavus* [41].

In addition to their antimicrobial efficacy, Schiff bases containing fluorine substituents have been reported to exhibit notable cytotoxic effects against human cancer cell lines. A series of Schiff bases containing fluorine or trifluoromethyl groups in their molecular structure exhibited cytotoxic activity against three human cancer cell lines, including HeLa, A549, and BGC‐823 [42].

In another study, several Schiff bases were synthesized, and the compound bearing a trifluoromethyl substituent exhibited the highest biological activity against A549, HeLa, and MCF‐7 cell lines [43].

Today, many marketed drugs contain fluorine atoms or fluorinated groups. This is reflected in the high proportion of fluorine‐containing FDA‐approved pharmaceuticals over the past decade, highlighting the key role of fluorine in drug development [36, 44, 45].

These drugs are used in therapeutic areas including oncology (adagrasib [46], ensartinib [47], inavolisib [48], pirtobrutinib [49], revumenib [50], tovorafenib [51], trametinib [52], vorasidenib [53]), cardiovascular disease (acoramidis [54], vericiguat [55]), neurology and psychiatry (atogepant [56], lasmiditan [57], lemborexant [58], lumateperone [59], pimavanserin [60], ubrogepant [61]), immunology (berotralstat [62], etrasimod [63], leniolisib [64], siponimod [65], upadacitinib [66]), hematology (danicopan [67]), infectious diseases (baloxavir marboxil [68], bictegravir [69], delafloxacin [70], doravirine [71], eravacycline [72], glecaprevir/pibrentasvir [73], letermovir [74], nirmatrelvir [75]), gynecological conditions (elagolix [76]) and metabolic diseases (osilodostrat [77]). A notable advancement occurred in 2023 when the FDA‐approved omaveloxolone as the first treatment for Friedreich's ataxia, emphasizing the importance of fluorine‐derived compounds in rare disease therapy [78].

As part of modern fluorination strategies, the trifluoromethyl (—CF_3_) group has been increasingly added to scaffolds because of its strong electronic effects and lipophilic nature [36, 79].

This group can shape the way molecules interact with their specific targets, modifying their stability, selectivity, and overall enhancing binding affinity and biological activity [80].

In the context of pharmaceutical research, where palladium complexes with aromatic Schiff base ligands offer a potential alternative to platinum‐based therapies and fluorination enhances bioactivity, palladium–fluorine complexes represent a promising direction for further investigation. The available scientific literature describes a variety of palladium complexes based on fluorine‐substituted Schiff bases. Following this line of research, our team has previously synthesized two palladium complexes with Schiff bases derived from 2‐chloro‐5‐(trifluoromethyl)aniline [81, 82], and the present study is a continuation of this work.

Using newly designed ligands derived from 2‐chloro‐5‐(trifluoromethyl)aniline, four palladium(II) complexes were synthesized. They were characterized using various physicochemical methods and the resulting crystals were analyzed by single‐crystal X‐ray diffraction. Additionally, their cytotoxic effects were evaluated on *Triticum aestivum* L. and *Artemia franciscana* Kellogg.

## 2 Experimental

### 2.1 Materials

The reagents 2‐chloro‐5‐(trifluoromethyl)aniline (98%), 2,3‐dihydroxybenzaldehyde (97%), pyridoxal hydrochloride (99%) (Sigma–Aldrich, Munich, Germany), 4‐benzoyl‐3‐methyl‐1‐phenyl‐5‐pyrazolinone (98%) (Thermo Fisher Scientific, Waltham, MA, USA), 2‐hydroxy‐3‐methoxybenzaldehyde (98%), and Pd(CH_3_COO)_2_ (98%) (Merck, Darmstadt, Germany) were used as supplied.

### 2.2 Ligands Synthesis

The condensation of 2‐chloro‐5‐(trifluoromethyl)aniline with 2,3‐dihydroxybenzaldehyde, 2‐hydroxy‐3‐methoxybenzaldehyde, pyridoxal hydrochloride, and 4‐benzoyl‐3‐methyl‐1‐phenyl‐5‐pyrazolinone in 1:1 or 1:2 molar ratios in ethanol led to the formation of ligands **HL** ^**1**^–**HL** ^**4**^.

#### 2.2.1 3‐((2‐Chloro‐5‐(trifluoromethyl)phenyl)iminomethyl)benzene‐1,2‐diol (HL^1^)

2,3‐Dihydroxybenzaldehyde (0.138 g, 1 mmol) was dissolved in ethanol (10 mL) and reacted with 2‐chloro‐5‐(trifluoromethyl)aniline (0.27 mL, 2 mmol) under reflux with continuous stirring for 6 h. Fine orange crystals of the product were obtained after a few days of storage in a refrigerator.

Yield 85%; M.p. 155.3 °C; M.wt. 315.67 g/mol; Anal. Calc. for C_14_H_9_ClF_3_NO_2_: C, 53.26; H, 2.87; N, 4.43%. Found: C, 53.47; H, 2.52; N, 4.36%.

IR (KBr, cm^–1^): *ν*(C=N) 1620; *ν*(Ar–OH) 1114; *ν*(CF_3_) 1327.

^1^H NMR (DMSO‐d_6_, *δ* (ppm), *J* (Hz)): *δ* = 12.96 (s, 1H, OH); 9.34 (s, 1H, OH); 9.11 (s, 1H, CH=N); 8.05 (d, 1H, *J* = 0.8 Hz, H_Ar_); 7.84 (d, 1H, *J* = 8.1 Hz, H_Ar_); 7.67 (dd, 1H, *J* = 0.8 Hz, *J* = 7.7 Hz, H_Ar_); 7.17 (dd, 1H, *J* = 1.4 Hz, *J* = 8.1 Hz, H_Ar_); 7.00 (dd, 1H, *J* = 0.9 Hz, *J* = 7.8 Hz, H_Ar_); 6.84 (t, 1H, *J* = 7.8 Hz, H_Ar_) ppm.

^13^C NMR (DMSO‐d_6_, *δ* (ppm)): *δ* = 166.9; 149.5; 145.7; 145.5; 132.6; 131.0; 129.1 (q, CF_3_, *J*_*C‐F*_ = 32.4 Hz); 124.7; 124.3; 123.3; 119.9; 119.2; 119.1; 117.5 ppm.

#### 2.2.2 2‐((2‐Chloro‐5‐(trifluoromethyl)phenyl)iminomethyl)‐6‐Methoxy‐Phenol (HL^2^)

A solution of 2‐hydroxy‐3‐methoxybenzaldehyde (0.152 g, 1 mmol) in ethanol (10 mL) was combined with 2‐chloro‐5‐(trifluoromethyl)aniline (0.27 mL, 2 mmol), and the resulting mixture was refluxed for 6 h. It was then stored at 4–6 °C for a few days, during which yellow crystals formed.

Yield 82%; M.p. 135.6 °C; M.wt. 329.70 g/mol; Anal. Calc. for C_15_H_11_ClF_3_NO_2_: C, 54.64; H, 3.36; N, 4.24%. Found: C, 54.73; H, 3.28; N, 4.15%.

IR (KBr, cm^–1^): *ν*(C=N) 1614; *ν*(Ar–OH) 1229; *ν*(CF_3_) 1330; *ν*_as_(Ar–O–C_aliphatic_) 1194; *ν*_s_(Ar–O–C_aliphatic_) 1085.

^1^H NMR (DMSO‐d_6_, *δ* (ppm), *J* (Hz)): *δ* = 12.92 (s, 1H, O*H*); 9.10 (s, 1H, CH=N); 8.01 (s, 1H, H_Ar_); 7.81 (d, 1H, H_Ar_, *J* = 10.0); 7.65 (d, 1H, H_Ar_, *J* = 10.0); 7.29 (d, 1H, H_Ar_, *J* = 10.0); 7.18 (d, 1H, H_Ar_, *J* = 10.0); 6.94 (t, 1H, H_Ar_, *J* = 5.0, 10.0); 3.83 (s, 3H, H_Ar_).

^13^C NMR (DMSO‐d_6_, *δ* (ppm)): *δ* = 166.6; 150.8; 147.9; 145.6; 132.6; 130.9; 129.1; 124.3; 124.2; 123.6; 119.0; 118.9; 117.2; 116.3; 55.9.

#### 2.2.3 4‐((2‐Chloro‐5‐(trifluoromethyl)phenyl)iminomethyl)‐5‐(hydroxymethyl)‐2‐Methyl‐Pyridin‐3‐Ol (HL^3^)

To a solution of pyridoxal hydrochloride (0.204 g, 1 mmol) in ethanol (10 mL) was added 2‐chloro‐5‐(trifluoromethyl)aniline (0.13 mL, 1 mmol), followed by the addition of sodium acetate (0.10 g). After heating the mixture under reflux for 3 h, it was stored in the refrigerator, where yellow crystals formed over a few days.

Yield 83%; M.wt. 344.72 g/mol; Anal. Calc. for C_15_H_12_ClF_3_N_2_O_2_: C, 52.25; H, 3.50; N, 8.12%. Found: C, 52.48; H, 3.36; N, 8.04%.

IR (KBr, cm^–1^): *ν*(C=N) 1611; *ν*(Ar–OH) 1144; *ν*(CF_3_) 1329; *ν*(C–OH) 1087.

^1^H NMR (DMSO‐d_6_, *δ* (ppm), *J* (Hz)): *δ* = 13.57 (s, 1H, OH); 9.27 (s, 1H, CH=N); 8.12 (s, 1H, H_Ar_); 8.05 (s, 1H, H_Py_); 7.90 (d, 1H, *J* = 8.4 Hz, H_Ar_); 7.77 (d, 1H, *J* = 8.4 Hz, H_Ar_); 5.46 (t, 1H, *J* = 5.4 Hz, OH); 4.84 (d, 1H, *J* = 5.4 Hz, CH_2_); 2.45 (s, 3H, CH_3_) ppm.

#### 2.2.4 4‐(N‐(2‐Chloro‐5‐(trifluoromethyl)phenyl)‐C‐Phenyl‐Carbonimidoyl)‐5‐Methyl‐2‐Phenyl‐4H‐Pyrazol‐3‐One (HL^4^)

An ethanolic solution (10 mL) of 4‐benzoyl‐3‐methyl‐1‐phenyl‐5‐pyrazolinone (0.278 g, 1 mmol) was treated with 2‐chloro‐5‐(trifluoromethyl)aniline (0.27 mL, 2 mmol) and refluxed for 9 h. The mixture was then cooled and stored at 4–6 °C, allowing yellow crystals to form.

Yield 85%; M.p. 224.4 °C; M.wt. 455.85 g/mol; Anal. Calc. for C_24_H_17_ClF_3_N_3_O: C, 63.23; H, 3.75; N, 9.22%. Found: C, 63.40; H, 3.54; N, 9.18%.

IR (KBr, cm^–1^): *ν*(C=O) 1635; *ν*(N–H) 3,104; *ν*(CF_3_) 1329.

^1^H NMR (DMSO‐d_6_, *δ* (ppm), *J* (Hz)): *δ* = 12.79 (bs, 1H, NH); 8.00 (d, *J* = 7.9 Hz, 2H, H_Ar_); 7.77 (d, *J* = 8.4 Hz, 1H, H_Ar_); 7.54–7.47 (m, 6H, H_Ar_); 7.44 (t, *J* = 7.9 Hz, 2H, H_Ar_); 7.20–7.18 (m, 2H, H_Ar_); 1.5 (s, 3H, CH_3_) ppm.

^13^C NMR (DMSO‐d_6_, *δ* (ppm)): *δ* = 165.1 (C‐7); 162.5; 147.8; 138.5; 135.5; 132.5; 130.9; 130.8; 130.2; 128.9; 128.7; 128.4; 127.8 (q, *J*_*C‐F*_ = 33.0 Hz); 124.3; 124.1; 123.9; 118.2; 102.3; 15.7 ppm.

### 2.3 Synthesis of Palladium(II) Complexes (**1**–**4**)

Palladium(II) complexes were formed upon reaction of Pd(CH_3_COO)_2_ with **HL** ^**1**^–**HL** ^**4**^.

#### 2.3.1 [Pd(L^1^)_2_] (1)

The ligand **HL** ^**1**^ (0.315 g, 1 mmol) and Pd(CH_3_COO)_2_ (0.224 g, 1 mmol) were dissolved separately in 10 and 5 mL of acetonitrile, respectively. The solutions were mixed and stirred for 4 h at 50 °C, resulting in precipitation of a brown solid. Following filtration, the precipitate was washed with ether and air‐dried.

Yield 76%; M.p. 249.8 °C; M.wt. 735.75 g/mol; Anal. Calc. for C_28_H_16_Cl_2_F_6_N_2_O_4_Pd: C, 45.70; H, 2.19; N, 3.80; Pd, 14.46. Found: C, 45.74; H, 2.07; N, 3.72; Pd, 14.28%.

IR (KBr, cm^–1^): *ν*(C=N) 1600; *ν*(Ar–OH) –; *ν*(CF_3_) 1329.

#### 2.3.2 [Pd_2_(L^2^)_2_(CH_3_COO)_2_] (2)

A methanolic solution (5 mL) of Pd(CH_3_COO)_2_ (0.224 g, 1 mmol) was mixed with 10 mL chloroform/methanol solution (1:5, v/v) of **HL** ^**2**^ (0.329 g, 1 mmol) and then stirred at 50 °C for 4 h. Subsequently, the suspension was filtered, resulting in a brown precipitate. For recrystallization, a CH_3_OH/CHCl_3_ (1:1, v/v) mixture was used.

Yield 72%; M.p. 236.1 °C; M.wt. 988.27 g/mol; Anal. Calc. for C_34_H_26_Cl_2_F_6_N_2_O_8_Pd_2_: C, 41.31; H, 2.65; N, 2.83; Pd, 21.53. Found: C, 41.47; H, 2.49; N, 2.73; Pd, 21.42%.

IR (KBr, cm^–1^): *ν*(C=N) 1602; *ν*(Ar–OH) 1210; *ν*(CF_3_) 1329; *ν*_as_ (COO^−^) 1542; *ν*_s_ (COO^−^) 1389; *ν*_as_(Ar–O–C_aliphatic_) 1191; *ν*_s_(Ar–O–C_aliphatic_) 1083.

#### 2.3.3 [Pd(L^3^)_2_] (3)

To 10 mL of an acetonitrile solution of **HL** ^**3**^ (0.344 g, 1 mmol), 5 mL of an acetonitrile solution of Pd(CH_3_COO)_2_ (0.224 g, 1 mmol) was added. The reaction medium was maintained under reflux for 6 h, yielding a brown precipitate. This solid was separated by filtration, washed with ether, and air‐dried.

Yield 74%; M.wt. 793.83 g/mol; Anal. Calc. for C_30_H_22_Cl_2_F_6_N_4_O_4_Pd: C, 45.38; H, 2.79; N, 7.05; Pd, 13.40. Found: C, 45.47; H, 2.58; N, 6.89; Pd, 13.26%.

IR (KBr, cm^–1^): *ν*(C=N) 1600; *ν*(Ar–OH) 1131; *ν*(CF_3_) 1329; *ν*_as_(C–OH) 1083.

#### 2.3.4 [Pd(L^4^)_2_] (4)

To a solution of **HL** ^**4**^ (0.455 g, 1 mmol) in CHCl_3_/CH_3_OH (1:5, v/v, 10 mL), a methanolic solution (5 mL) of Pd(CH_3_COO)_2_ (0.224 g, 1 mmol) was added. After stirring at 50 °C for 4 h, a pale brown precipitate was obtained, which was isolated by filtration and recrystallized from DMSO/CH_3_OH (1:1, v/v).

Yield 70%; M.p. 344.2 °C; M.wt. 1016.09 g/mol; Anal. Calc. for C_48_H_32_Cl_2_F_6_N_6_O_2_Pd: C, 56.73; H, 3.17; N, 8.27; Pd, 10.47. Found: C, 56.80; H, 3.01; N, 8.15; Pd, 10.32%.

IR (KBr, cm^–1^): *ν*(C=O) 1595; *ν*(N–H) –; *ν*(CF_3_) 1327.

### 2.4 Physical Measurements

Infrared spectra were obtained using the KBr pellet technique on a Bruker Vertex 70 spectrophotometer in the range of 4000–400 cm^–1^. UV–vis spectra were measured in diffuse reflectance mode on a Jasco V‐670 spectrophotometer. Nuclear magnetic resonance spectra were recorded on a Bruker DRX 400 spectrometer using TMS as the reference. Elemental analysis for carbon, hydrogen, and nitrogen content was carried out with a Carlo‐Erba LA‐118 microanalyzer, while palladium content was determined following the method described by Fries and Getrost [83]. Magnetic susceptibilities were measured with a homemade Faraday balance. Thermal stability and decomposition of the complexes were studied under synthetic air using a TGA 2 Star System thermogravimetric analyzer (Mettler Toledo). A Boetius apparatus was used to determine the melting points. The mass spectra were recorded using a Xevo TQ‐S Cronos (Waters Corporation) mass spectrometer. Finally, molar conductivity in DMF solution (10^–3^ M) at room temperature was determined using a Consort C‐533 conductometer.

### 2.5 X‐ray Crystallographic Analysis

Single‐crystal X‐ray diffraction characterization of complexes **2** and **4** was carried out on a Rigaku Oxford‐Diffraction XCALIBUR E CCD diffractometer with Mo Kα radiation. The crystal data for the ligands were recorded on an XtaLAB Synergy, Dualflex, HyPix diffractometer with Cu Kα radiation. CrysAlis software package from Oxford Diffraction was used for unit cell characterization and data analysis [84]. Structure determination was performed by Intrinsic Phasing [85] in Olex2, with the SHELXT program [86], while optimization was performed by full‐matrix least‐squares refinement on *F* ^2^ with SHELXL‐2015 [87]. Nonhydrogen atoms were treated anisotropically. Hydrogens attached to carbon atoms were fixed at a bond length of 0.96 Å and refined via the riding model. Hydrogens of hydroxyl groups were identified from Fourier maps and adjusted according to hydrogen‐bonding geometries. Molecular structures were depicted through Olex2. Tables 1 and 2 summarize the crystallographic and refinement data for the studied compounds.

**TABLE 1:** Crystallographic and refinement data for **H** **L** ^**1**^–**H** **L** ^**4**^.

Compound	HL^1^	HL^2^	HL^3^	HL^4^
Empirical formula	C_14_H_9_ClF_3_NO_2_	C_15_H_11_ClF_3_NO_2_	C_15_H_12_ClF_3_N_2_O_2_	C_24_H_17_ClF_3_N_3_O
Formula weight	315.67	329.70	344.72	455.85
Temperature, K	100	293	100	100
Space group	*P*2_1_/*c*	*Pbca*	*P*2_1_	*C*2/*c*
*a,* Å	4.74686(5)	14.2400(10)	4.38004(9)	7.94922(13)
*b*, Å	17.33535(18)	6.3799(4)	12.2992(3)	23.0785(4)
*c*, Å	30.8269(3)	30.9467(19)	13.0684(3)	23.2173(4)
*α*, °	90	90	90	90
*β*, °	90.5227(9)	90	92.144(2)	94.5364(15)
*γ*, °	90	90	90	90
Volume, Å^3^	2536.59(5)	2811.5(3)	703.51(3)	4246.00(12)
*Z*	4	8	2	8
*ρ*_calc,_ g/cm^3^	1.653	1.558	1.627	1.426
*μ*, mm^–1^	3.084	0.312	2.855	2.021
Crystal size, mm^3^	0.40 × 0.03 × 0.03	0.30 × 0.06 × 0.03	0.50 × 0.02 × 0.02	0.20 × 0.10 × 0.10
2Θ range, °	5.734–149.008	6.298–50.036	6.768–153.56	7.64–133.182
Refl. collected	16,899	7514	8781	15,603
Indep. refls., *R*_int_	4983, 0.0271	2480, 0.0442	2692, 0.0610	3760, 0.0302
Data/rest./param.	4983/0/384	2480/0/200	2692/1/211	3760/0/290
*R*_1_ a, *wR*_2_ b (all data)	0.0415, 0.1082	0.0680, 0.1693	0.0353, 0.0919	0.0303, 0.0797
GOF^c^ on *F* ^2^	1.038	1.072	1.026	1.029
Flack parameter	—	—	0.044(12)	—

a *R*_1 _= Σ||*F*_o_| − |*F*_c_||/Σ|*F*_o_|.

b *wR*_2 _= {Σ[*w*(*F*_o_^2^ − *F*_c_^2^)^2^]/Σ[*w*(*F*_o_^2^)^2^]}^1/2^.

c GOF = {Σ[*w*(*F*_o_^2^ − *F*_c_^2^)^2^]/(*n* − *p*)}^1/2^, where *n* is the number of reflections and *p* is the total number of parameters refined.

**TABLE 2:** Crystallographic and refinement data for complexes **2** and **4**.

Compound	**[Pd**_**2**_ **(L** ^**2**^ **)**_**2**_ **(CH**_**3**_ **COO)**_**2**_ **] (2)**	**[Pd(L** ^**4**^ **)**_**2**_ **] (4)**
Empirical formula	C_34_H_26_Cl_2_F_6_N_2_O_8_Pd_2_	C_48_H_32_Cl_2_F_6_N_6_O_2_Pd
Formula weight	988.27	1016.09
Temperature, K	293	100
Space group	*P*2_1_2_1_2_1_	*P*2_1_/*n*
*a*, Å	13.6162(7)	6.57430(9)
*b*, Å	16.4874(7)	14.33700(19)
*c*, Å	16.6751(7)	22.8142(3)
*α*, °	90	90
*β*, °	90	92.4493(12)
*γ*, °	90	90
Volume, Å^3^	3743.5(3)	2148.40(5)
*Z*	4	2
*ρ*_calc,_ g/cm^3^	1.754	1.571
*μ*, mm^−1^	1.185	5.274
Crystal size, mm^3^	0.15 × 0.05 × 0.03	0.06 × 0.05 × 0.03
2Θ range, °	3.474–58.202	7.284–133.182
Refl. collected	15,564	13,666
Indep. refls., *R*_int_	8198, 0.0809	3791, 0.0332
Data/rest./param.	8198/50/404	3791/0/294
*R*_1_ a, *wR*_2_ b (all data)	0.0801, 0.1212	0.0463, 0.1152
GOF^c^ on *F* ^2^	0.969	1.067
Flack parameter	0.00(4)	—

a *R*_1 _= Σ||*F*_o_| − |*F*_c_||/Σ|*F*_o_|.

b *wR*_2 _= {Σ[*w*(*F*_o_^2^ − *F*_c_^2^)^2^]/Σ[*w*(*F*_o_^2^)^2^]}^1/2^.

c GOF = {Σ[*w*(*F*_o_^2^ − *F*_c_^2^)^2^]/(*n* − *p*)}^1/2^, where *n* is the number of reflections and *p* is the total number of parameters refined.

### 2.6 Cellular Toxicity Evaluation

#### 2.6.1 Plant Toxicity Assessment

Both ligands and the corresponding palladium(II) complexes were assessed for phytotoxic effects on *Triticum aestivum* L. rootlets through Constantinescu method [88]. Controlled laboratory conditions were used to germinate seeds, and rootlet length was measured to evaluate the impact of compound concentration, molecular structure, and exposure time.

Data were collected across three consecutive days, using day 1 and ligand **HL** ^**1**^ as reference points. A total of five distinct concentrations were applied (0.1, 1, 10, 50, and 100 μM), with 1% DMSO as a solvent. Since a minor inhibitory effect of the solvent was observed compared to that of pure water, it was used as a negative control.

Root tips were treated with acetic orcein for staining [89], and the squash technique was applied to prepare them for microscopic evaluation [90]. A Nikon Labophot‐2 light microscope was used to examine the samples.

Statistical analyses were conducted using R (v4.5.1). A linear regression model was used to assess the influence of substance, concentration, and measurement day on root length, all treated as factors, so as to obtain the equivalent of an ANOVA model. Model structure was determined by comparing a full interaction model against a main‐effects‐only model, with the latter being selected based on a better Bayesian information criterion value for improved parsimony and reduction of noise from minor, nonplausible interactions. To ensure the validity of standard error estimation, two robust modeling techniques were utilized. Initial coefficient estimation was conducted via ordinary least squares (OLS), with robust inference achieved through the application of the HC3 heteroscedasticity‐consistent standard error correction, a method specifically chosen for its performance in finite samples [91]. Additionally, we used robust regression via the lmrob() function from the “robustbase” R package [92] to account for potential outliers and heavy‐tailed errors in the dataset. This model uses M‐estimation, a method that yields robust regression coefficient estimates by reducing the influence of outliers [93, 94]. The robust regression model delivers more reliable coefficient estimates in the presence of influential outliers while also accounting for heteroscedasticity [95]. Standard errors for these robust estimates were obtained directly from the lmrob() function (from “robustbase”), which simultaneously adjusts for both heteroscedasticity and the influence of outliers. Both the OLS model with robust standard errors and the robust regression model produced consistent results, reinforcing the stability of the estimated coefficients and the validity of statistical inference despite potential heteroscedasticity. Model visualization (including plots of coefficients and predicted values with 95% confidence intervals) was carried out using the sjPlot package [96], with ggplot_2_ [97] employed for enhanced graphical customization.

#### 2.6.2 Animal Toxicity Assessment

The lethality assay on *A. franciscana* Kellogg was performed to evaluate the acute toxicity of the newly synthesized compounds [98, 99].

Mortality of crustacean nauplii was recorded at 24 and 48 h following exposure to solutions of the compounds prepared in 1% DMSO at five concentrations (100 µM–C_1_; 50 µM–C_2_; 25 µM–C_3_; 12.5 µM–C_4_; and 6.5 µM–C_5_) to determine the median lethality concentration lethality (i.e., the concentration causing 50% of the exposed population) [100, 101].

Dose–response relationships were modeled using the three‐parameter Weibull type 1 (W1.3) function in the R package “drc” [102], selected based on the lowest Akaike information criterion and a lack‐of‐fit *p*‐value of 1.0 from the mselect() function.

## 3 Results and Discussion

### 3.1 General Aspects

According to the analytical results, complexes **1**, **3,** and **4** have a 1:2 metal‐to‐ligand ratio, whereas complex **2** has a 1:1 ratio. They are stable at room temperature and decompose only at temperatures above 236 °C. The molar conductivity data were found to be in the range of 7–20 Ω^−1 ^cm^2 ^mol^−1^ which evidence the nonelectrolitic behavior of the complexes [103].

The palladium(II) complexes are soluble in DMSO, CHCl_3_, and CH_2_Cl_2_.

### 3.2 Crystal Structure Description for Ligands **H** **L** ^**1**^–**H** **L** ^**4**^ and Complexes **2** and **4**

Suitable crystals were grown for **HL** ^**1**^–**HL** ^**4**^ organic proligands, and their structures in the solid state were elucidated using single‐crystal X‐ray diffraction. Figures 1, 2, 3, 4 show the X‐ray crystallographic structures of the four proligands, while Table S1 lists the corresponding geometric parameters. All of them exhibit a molecular crystal structure comprising one molecular unit in the asymmetric part of the unit cell except **HL** ^**1**^, which crystallizes with two crystallographically independent but chemically identical molecules (denoted as **A** and **B**). The crystals **HL** ^**1**^, **HL** ^**2**^, and **HL** ^**4**^ are centrosymmetric, while **HL** ^**3**^ crystallizes as pure enantiomer in a Sohncke space group *P*2_1_ with Flack parameter 0.044(12). No interstitial solvent molecules were found in the crystals. The conformation of each molecule is stabilized by classical intramolecular hydrogen bonds: O‐H···Cl, O‐H···N in **HL** ^**1**^–**HL** ^**3**^, and N‐H···O in **HL** ^**4**^. **HL** ^**1**^ also presents intramolecular O‐H···O interactions. Table 3 lists the corresponding hydrogen‐bond parameters.

**FIGURE 1:**
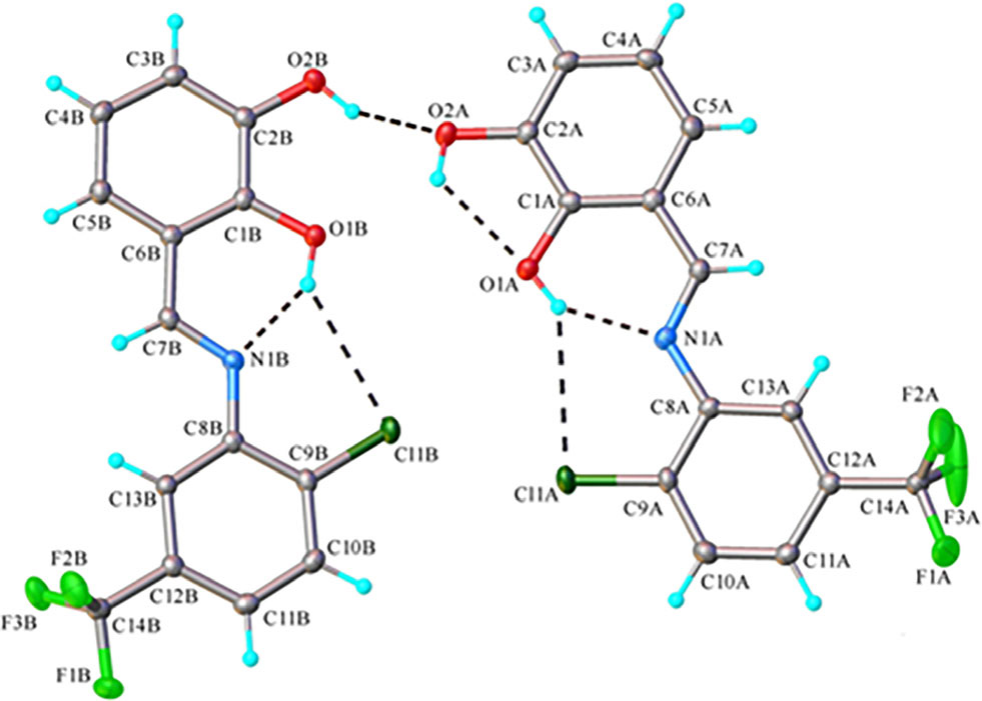
X‐ray molecular structure for **HL** ^**1**^ with atom labelling and thermal ellipsoids at 50% probability.

**FIGURE 2:**
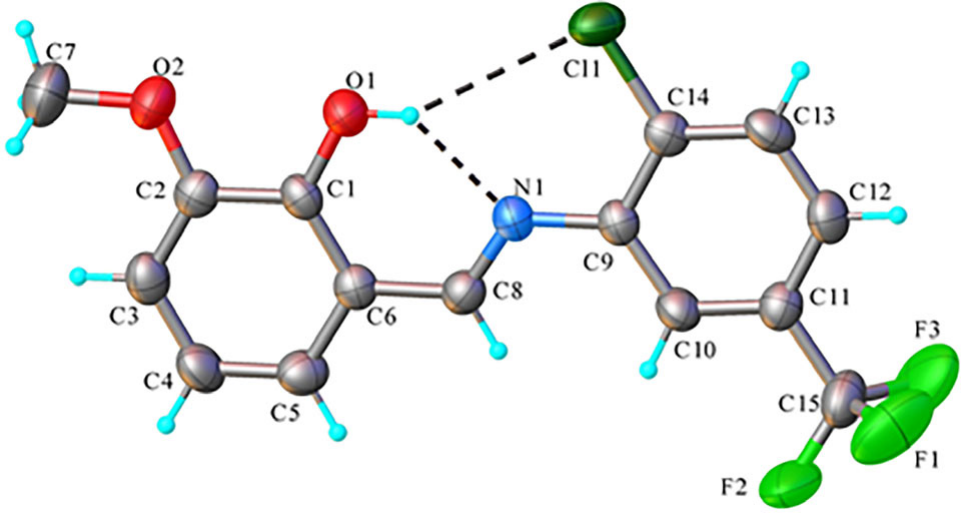
X‐ray molecular structure for **HL** ^**2**^ with atom labelling and thermal ellipsoids at 50% probability.

**FIGURE 3:**
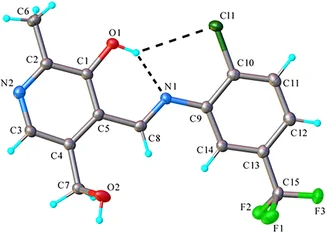
X‐ray molecular structure for **HL** ^**3**^ with atom labelling and thermal ellipsoids at 50% probability.

**FIGURE 4:**
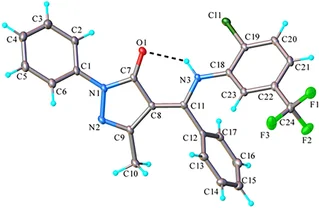
X‐ray molecular structure for **HL** ^**4**^ with atom labelling and thermal ellipsoids at 50% probability.

**TABLE 3:** Intramolecular hydrogen bonds for **H** **L** ^**1**^–**H** **L** ^**4**^.

D‐H···A	*d*_D‐H_, Å	*d*_H‐A_, Å	*d*_D‐A_, Å	∠D‐H‐A, °
HL^1^				
O1A‐H···Cl1A	0.84	2.92	3.6253(14)	142.7
O1A‐H···N1A	0.84	1.88	2.616(2)	145.1
O2A‐H···O1A	0.84	2.28	2.7094(19)	112.0
O2A‐H···O1B	0.84	2.51	2.944(2)	113.2
O1B‐H···Cl1B	0.84	2.84	3.5378(14)	142.2
O1B‐H···N1B	0.84	1.87	2.599(2)	144.7
O2B‐H···O2A	0.84	2.19	2.901(2)	142.2
O2B‐H···O1B	0.84	2.26	2.7104(19)	113.6
HL^2^				
O1‐H···Cl1	0.86	2.82	3.604(3)	151.7
O1‐H···N1	0.86	1.92	2.609(4)	135.7
HL^3^				
O1‐H···Cl1	0.84	2.82	3.512(2)	140.6
O1‐H···N1	0.84	1.83	2.567(3)	144.9
HL^4^				
N3‐H···O1	0.88	1.99	2.6790(14)	134.4

The crystal structures of palladium(II) complexes **2** and **4** were also determined by X‐ray diffraction, each exhibiting a molecular crystal structure with one neutral entity in the asymmetric part, as depicted in Figures 5 and 6. Complex **4** is mononuclear, while complex **2** represents a dinuclear species where the two palladium(II) atoms are linked by two acetate anions that play the role of bidentate‐bridging ligands. Palladium(II) atoms in both complexes are tetra‐coordinated in slightly distorted square planar geometry. In the mononuclear complex **4**, palladium(II) is surrounded by N_2_O_2_ set of atoms provided by two deprotonated (L^4^)^−^ ligands. By contrary, in dinuclear complex **2**, both palladium(II) atoms exhibit O_3_N coordination environment being completed by one bidentate anionic ligand (L^2^)^−^ and two bidentate‐bridging acetate anions. It is also to be emphasized that the molecular structure of complex **2** is consolidated via π···π stacking involving adjacent aromatic rings with centroid‐to‐centroid distance of 3.771 Å (Figure 5).

**FIGURE 5:**
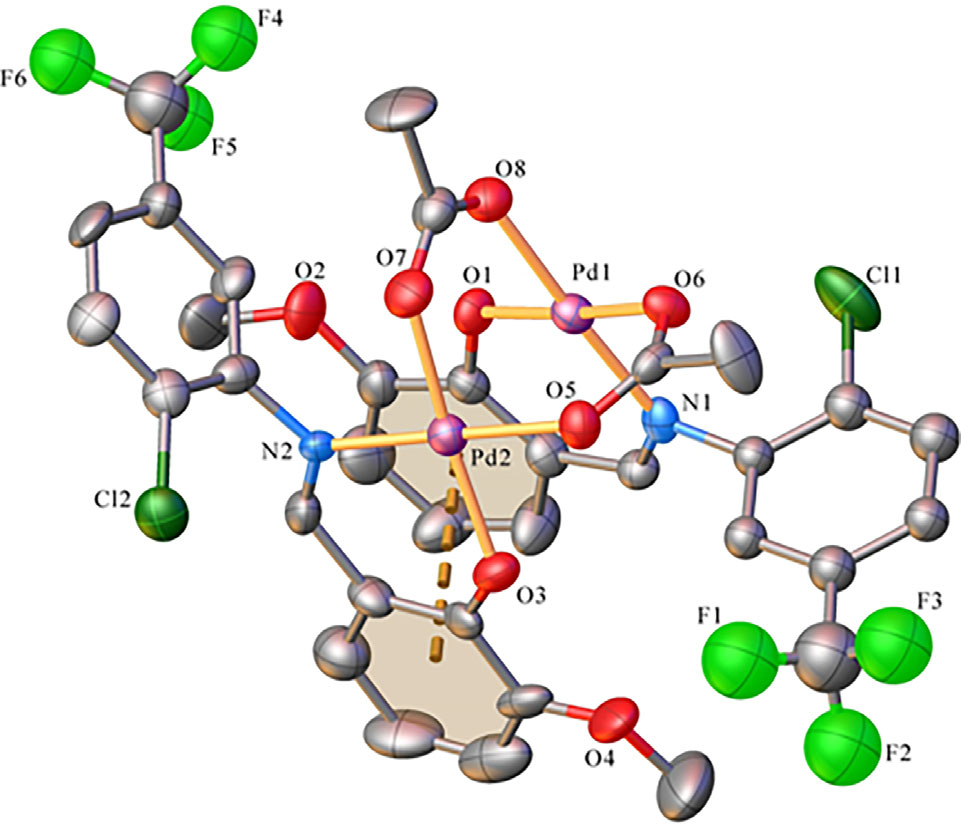
X‐ray molecular structure for complex **2** with atom labelling and thermal ellipsoids at 30% probability level. The centroid‐to‐centroid distance is shown in dashed‐brown line.

**FIGURE 6:**
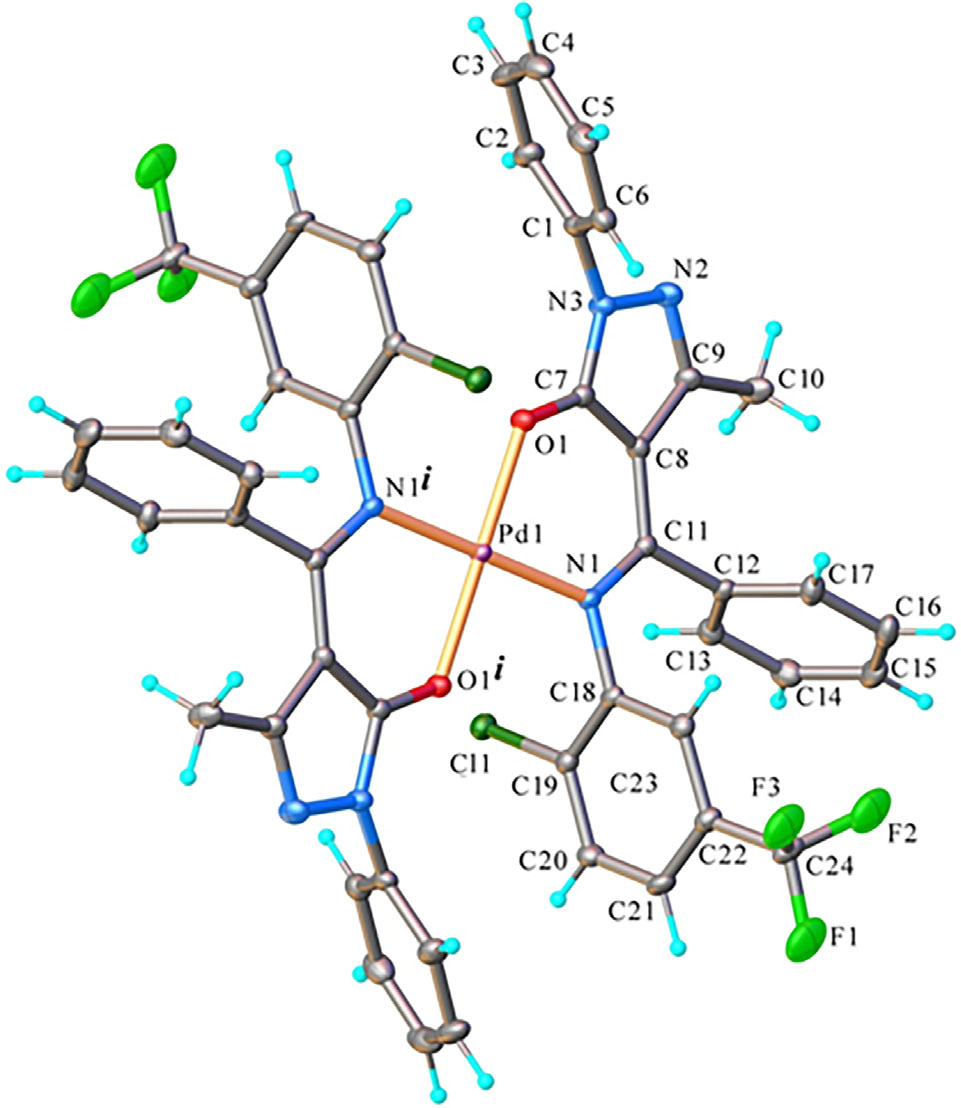
X‐ray molecular structure for complex **4** with atom labelling and thermal ellipsoids at 50% probability level.

### 3.3 Spectral Characterization

The IR spectra of the palladium(II) complexes exhibited strong ν(C=N) bands in the 1600–1602 cm^–1^ region, shifted 11–20 cm^–1^ to lower wavenumbers compared with the free ligands. This shift reflects coordination to the metal center through the azomethine nitrogen [104], with ligand **HL** ^**4**^ binding to the palladium ion in the amine–one form [105].

For ligands **HL** ^**1**^–**HL** ^**3**^, the *ν*(Ar–OH) band of the phenolic group, observed at 1114–1229 cm^–1^, shifted 13–19 cm^–1^ to lower frequencies or disappeared in the IR spectra of the palladium(II) complexes, indicating that coordination occurs via the phenolic oxygen [106].

Regarding **HL** ^**4**^ **,** a *ν*(C=O) band was observed at 1635 cm^–1^, which shifted to 1595 cm^–1^ upon complexation, indicating coordination via the exocyclic carbonyl oxygen.

The IR spectrum of complex **2** displayed new bands, which were assigned to the symmetric and asymmetric stretches of the carboxylate ion of the acetate group. The difference of about 153 cm^–1^ between the *ν*_as_(COO^–^) and *ν*_s_(COO^–^) vibrations is an indicator of the bridging coordination of the acetate [107].

For reference, the IR spectra of the ligands and their complexes are shown in Figures S1–S8.

The ligands were analyzed by ^1^H NMR and ^13^C NMR spectroscopy in DMSO as solvent (Figures S9–S15). In the ^1^H NMR spectra, the azomethine (CH=N) proton appeared as a singlet between 9.10 and 9.27 ppm, while the NH proton was observed as a broad singlet at 12.79 ppm. In the ^13^C NMR spectra, signals due to the imine (CH=N) carbons were detected in the range of 166.6–169.9 ppm, while those for the CF_3_ carbons were observed between 127.8 and 129.1 ppm. Additionally, the signal of the carbonyl (C=O) carbon appeared at 165.1 ppm.

In the UV–vis spectra of the free ligands, absorption bands attributed to intraligand π → π* and n → π* transitions were observed in the range of 41,666–22,988 cm^–1^. In the corresponding palladium(II) complexes, these bands were bathochromically shifted, indicating coordination of the ligands to the metal center through the azomethine nitrogen atom. Additionally, new absorption bands appeared between 23,529 and 20,202 cm^–1^ in the complexes. These bands are assigned to ligand‐to‐metal charge transfer and d–d transitions, notably the ^1^A_1g_→^1^A_2g_ transition, indicating a square‐planar geometry of the palladium ions [108]. Figures S16–S19 display the UV–vis spectra of all synthesized compounds. Diamagnetic behavior supports a square‐planar geometry for the palladium(II) complexes [109].

The mass spectra of complexes **1** and **3** were recorded (Figures S20 and S21), and the results were consistent with the proposed formulas. The spectra exhibit the molecular‐ion [M]^+^ peaks at *m*/*z* = 732.46 for complex **1** and *m*/*z* = 781.47 for complex **3**, along with a series of peaks corresponding to various fragments that are in good agreement with the proposed structures. A summary of the mass spectral data is presented in Table S2.

### 3.4 Thermal Analysis

The chemical composition of the resulting complexes was further confirmed by thermal decomposition.

The thermal degradation of complex **1** occurs in a single step (Figure 7), and the mass loss corresponds to the loss of the two ligand molecules (exp. 76.88%, calc. 83.36%), which occurs between 254 and 409 °C. The residue PdO represents 23.12% (calc. 16.64%).

**FIGURE 7:**
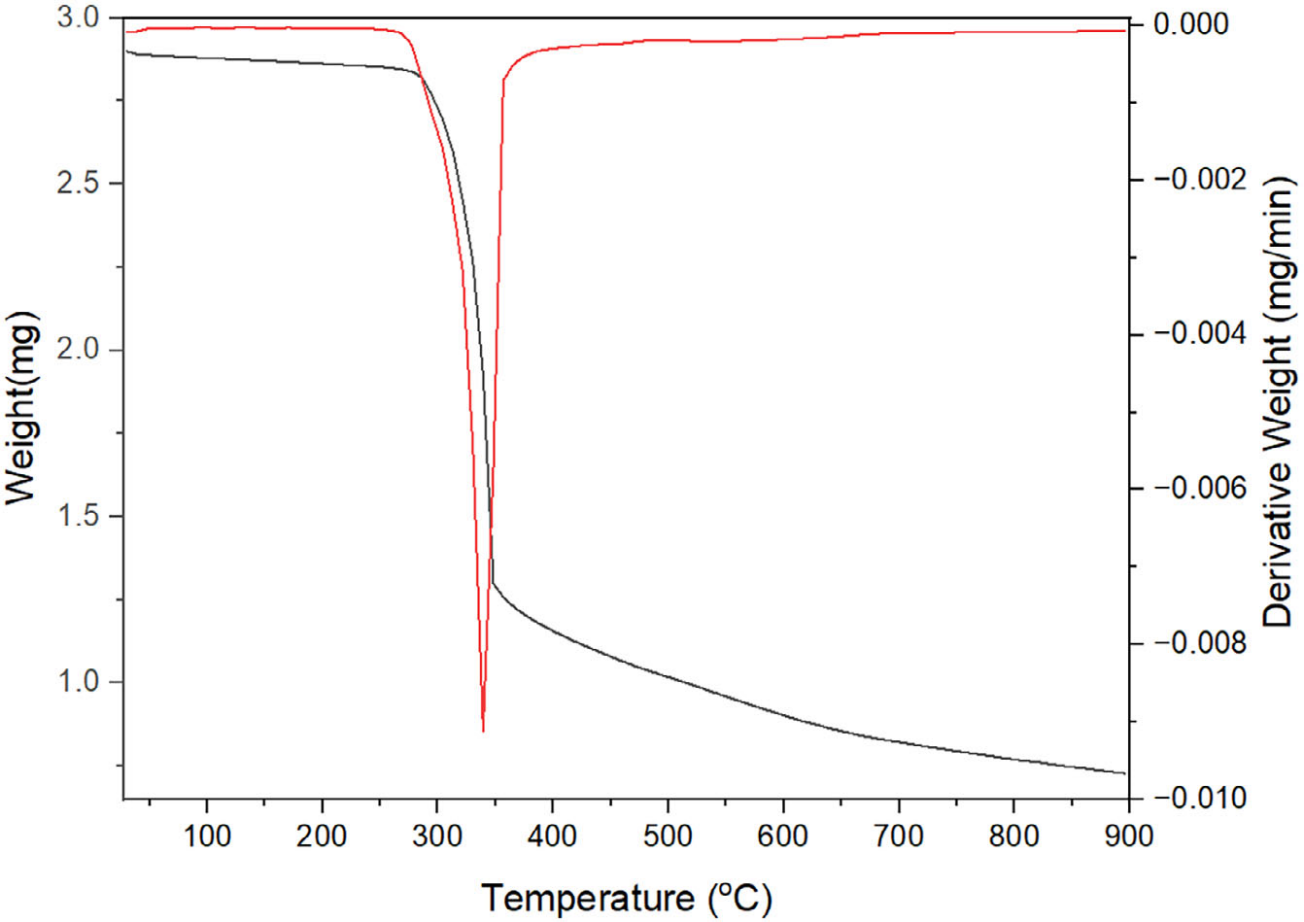
Thermogram of complex **1**.

The thermal degradation of complex **2** occurs in three steps (Figure 8). The first mass loss corresponds with the loss of two acetate molecules (exp. 12.69%, calc. 11.95%), and it occurs between 160 and 250 °C [110]. The next two mass losses occur in the temperature range of 250–800 °C and correspond to the loss of ligand molecules (exp. 55.13%, calc. 63.28%), and the residue (PdO) represents 32.18% (calc. 24.77%).

**FIGURE 8:**
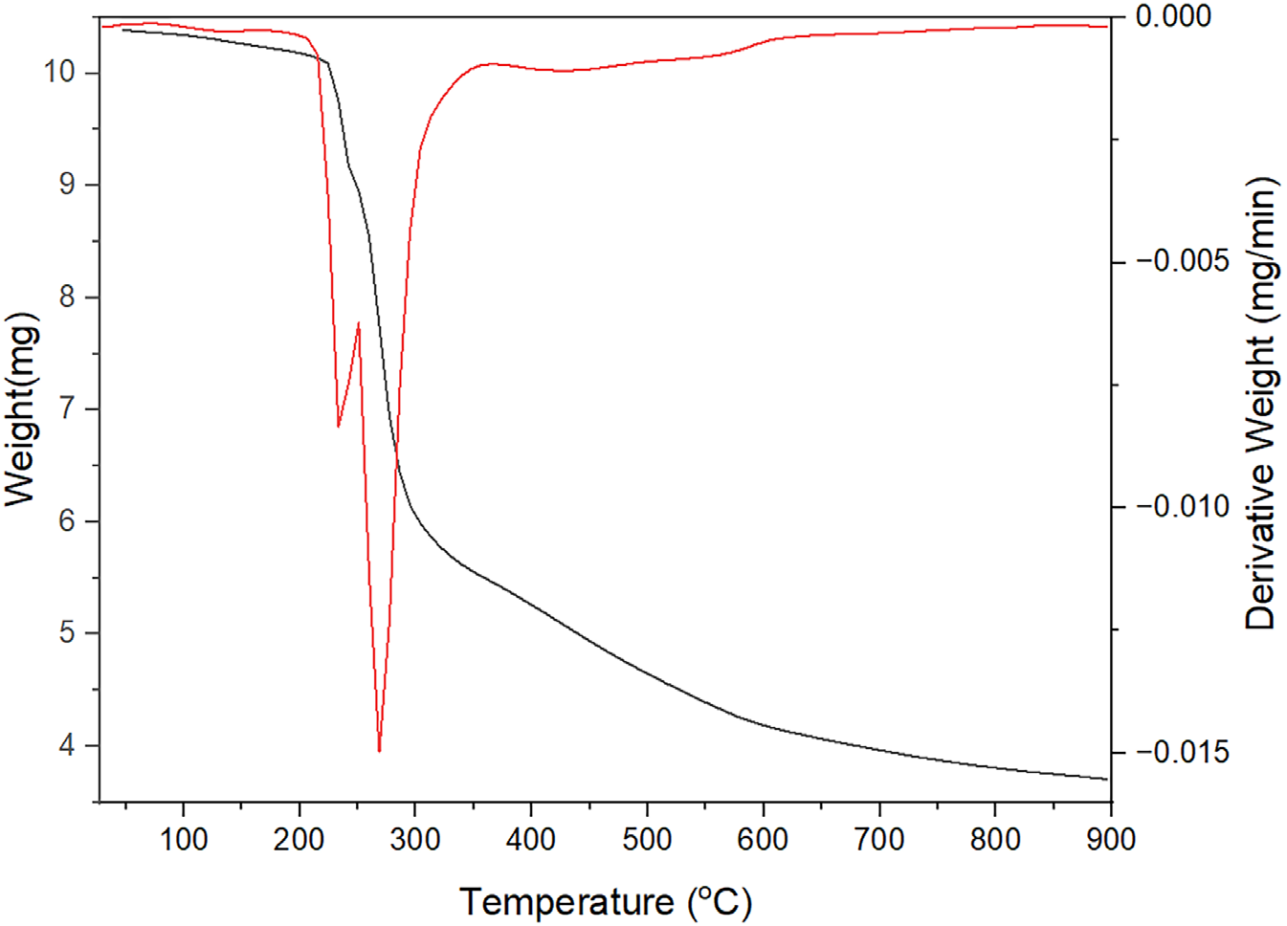
Thermogram of complex **2**.

The thermal degradation of complex **3** occurs in three steps (Figure S22). The first step corresponds to an acetonitrile molecule, indicating the loss of solvent adsorbed on the surface of the complex (exp. 3.33%, calc. 4.92%) [111]. The next two mass losses occur in the temperature range of 105–242 °C and correspond to the loss of two ligand molecules (exp. 57.79%, calc. 66.04%). These processes generate PdO along with an organic part as a residue (exp. 38.88%, calc. 29.04%).

The thermal degradation of complex **4** occurs in two steps (Figure S23). The first two mass losses correspond to the loss of ligand molecules (exp. 67.30%, calc. 76.13%), and they occur between 190 and 600 °C. The residue, PdO, along with an organic part, represents 32.70% (calc. 23.87%).

### 3.5 Phytotoxicity Evaluation

The standardized coefficients for the evaluated substances (both complexes and ligands), the tested concentrations, and the measurement days are presented in Figure 9.

**FIGURE 9:**
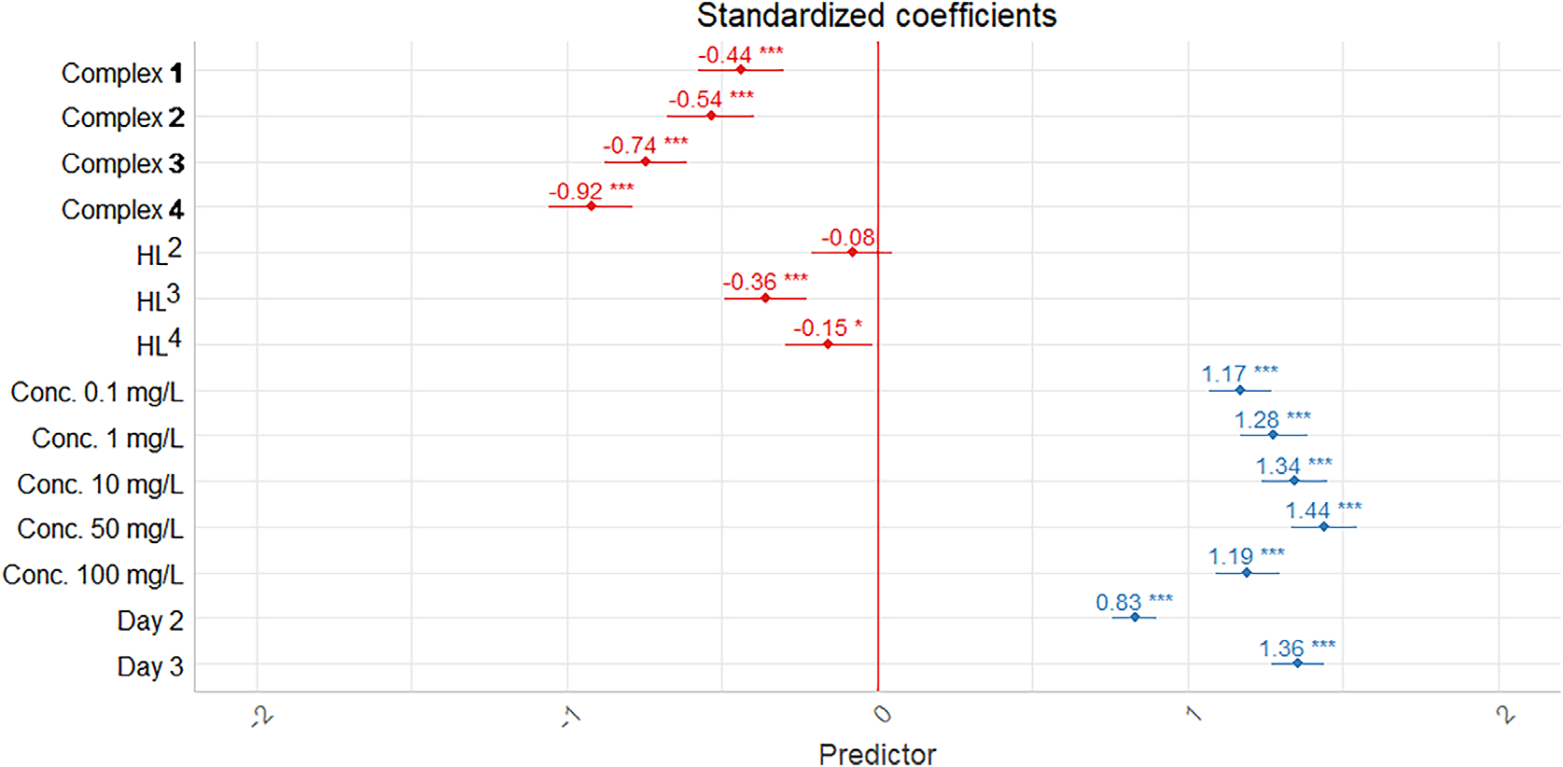
Standardized coefficients for the robust regression model with 95% confidence intervals. The plot shows the estimated effect sizes (standardized regression coefficients) of each predictor variable on the outcome, with the corresponding 95% confidence intervals based on the Wald method. Red vertical lines indicate the null hypothesis of zero effect, with coefficients and their confidence intervals marked. The size of each coefficient label corresponds to the magnitude of the effect, and stars indicate statistical significance based on the *p*‐values.

Overall, the complexes inhibited root growth more strongly than the ligands, although all substances showed small inhibitory effects. The difference between **HL** ^**1**^ and **HL** ^**2**^ was small and not statistically significant (*p* = 0.23), whereas the other ligands and all complexes exhibited more pronounced inhibition.

All tested concentrations tended to exert stimulatory effects compared to the negative control. The strongest effect was observed at 50 μM, with slightly lower effects at 10, 1, and 0.1 μM. Notably, the highest concentration, 100 μM, resulted in only a modest increase, similar to that at 0.1 μM, indicating a reduced stimulatory effect (a hormetic response).

At low concentrations, a slight stimulatory effect on growth was observed, but this effect diminished with increasing concentration, with the lowest growth recorded at the highest level (100 µM).

Growth increased over time, as reflected by the positive coefficients for measurement day, with a more pronounced increase on day 3 than on day 2 (for most variables and concentration levels, *p* < 0.001). The progression of root length over the three days of treatment is illustrated in Figures 10, 11, 12.

**FIGURE 10:**
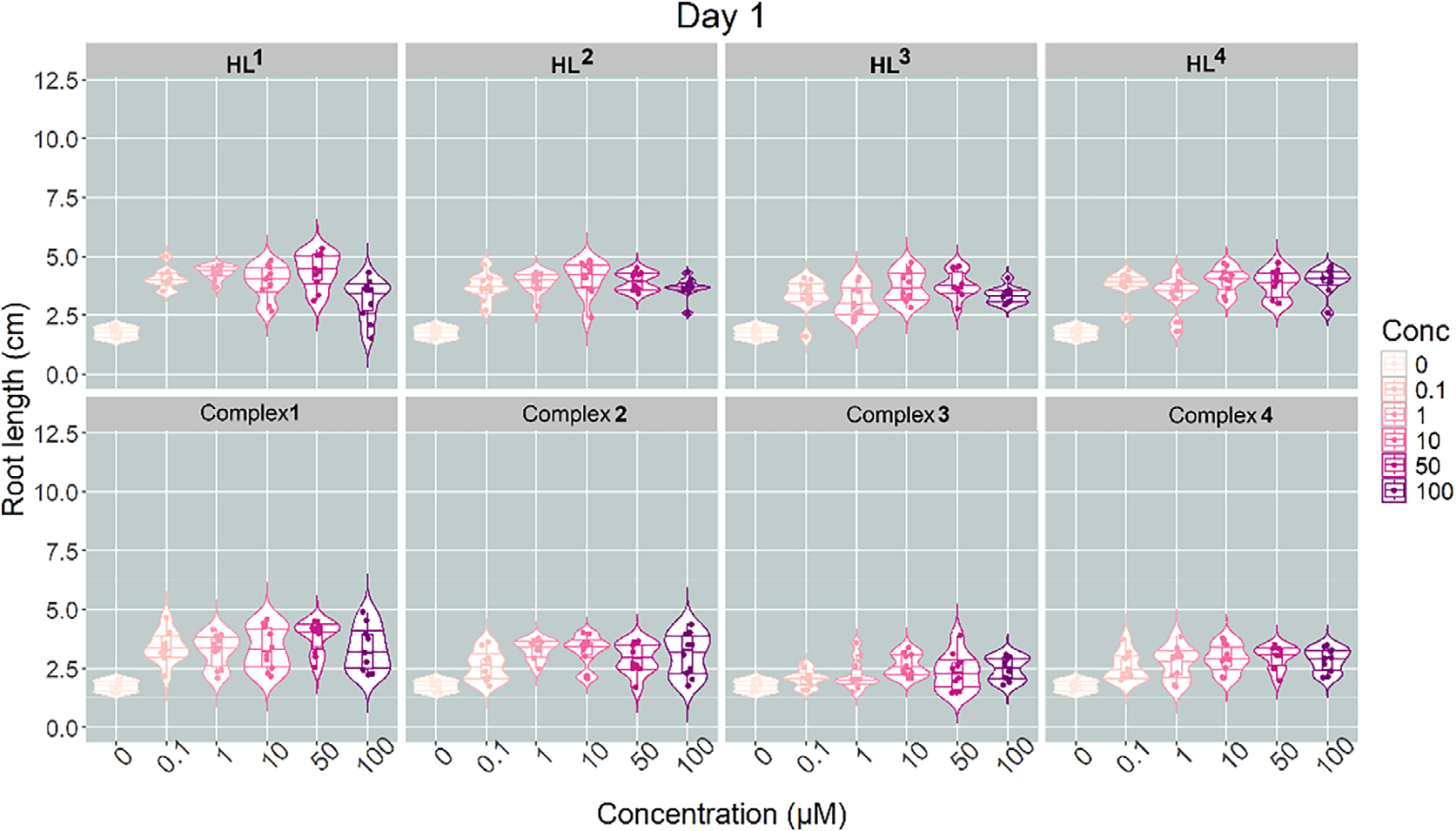
Root length progression of ligands and complexes on day 1 of treatment.

**FIGURE 11:**
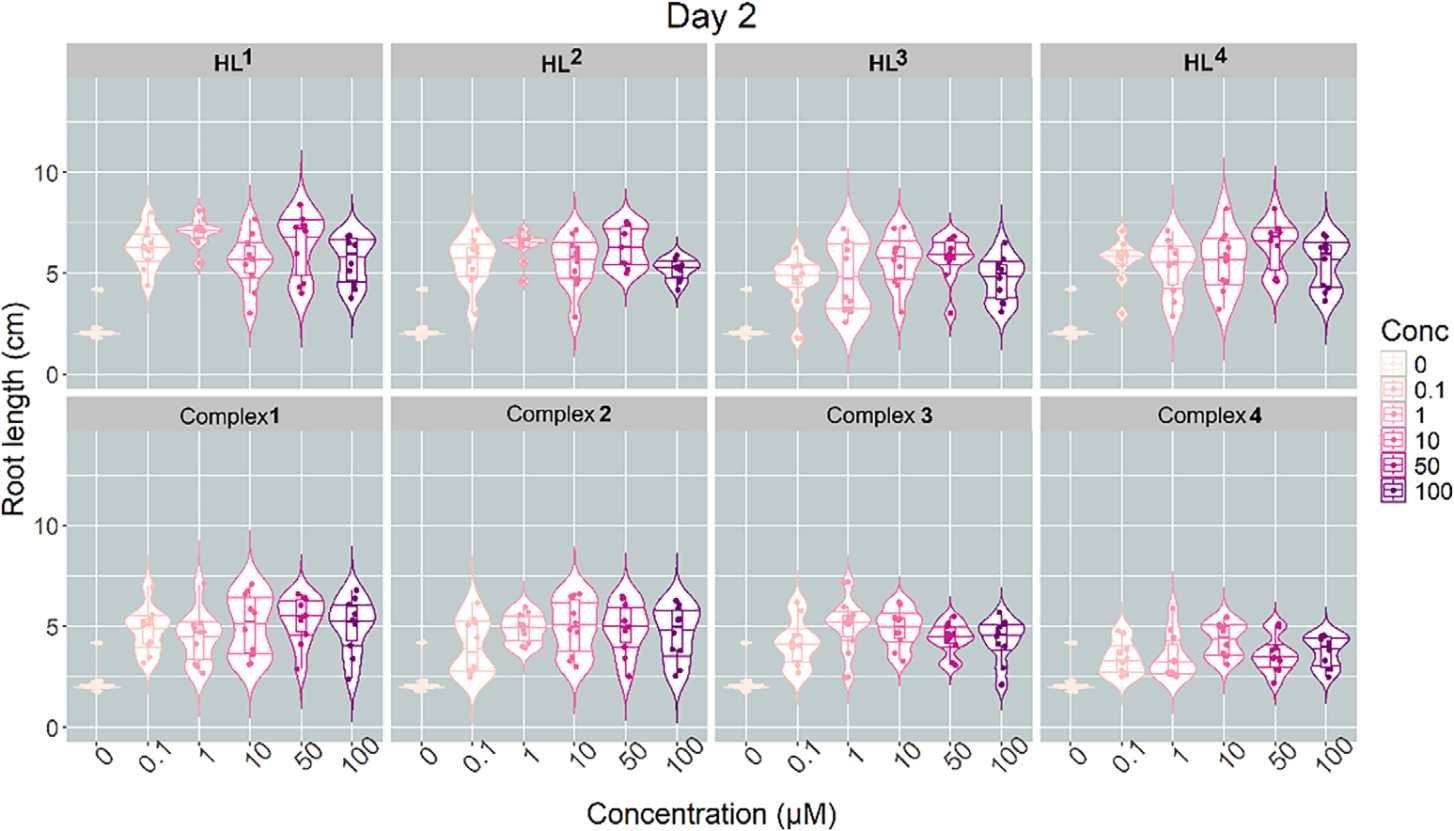
Root length progression of ligands and complexes on day 2 of treatment.

**FIGURE 12:**
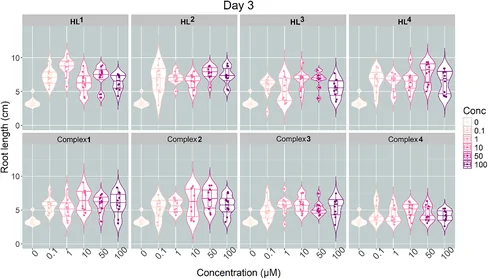
Root length progression of ligands and complexes on day 3 of treatment.

Because a clear concentration–response relationship was not observed, IC_50_ values could not be reliably estimated.

Given that root growth exceeded negative control levels at all concentrations and that the compounds’ negative coefficients were negligible compared to the larger, positive effects of concentration and time, we conclude that the compounds exert only minimal inhibitory effects.

Root growth analysis was also supported by microscopic evidence showing normal mitotic activity. Microscopy revealed active cell division across all phases, consistent with observed root growth. Moreover, the cells maintained their normal morphology similar to the control, indicating that no structural alterations occurred under the tested conditions.

Unusual mitotic figures, including certain metaphases, anaphases, and telophases in tropokinesis, were rarely noted, but their occurrence was similar to that observed in the control (1% DMSO) (Figures 13, 14, 15, 16; Figures S24 and S25).

**FIGURE 13:**
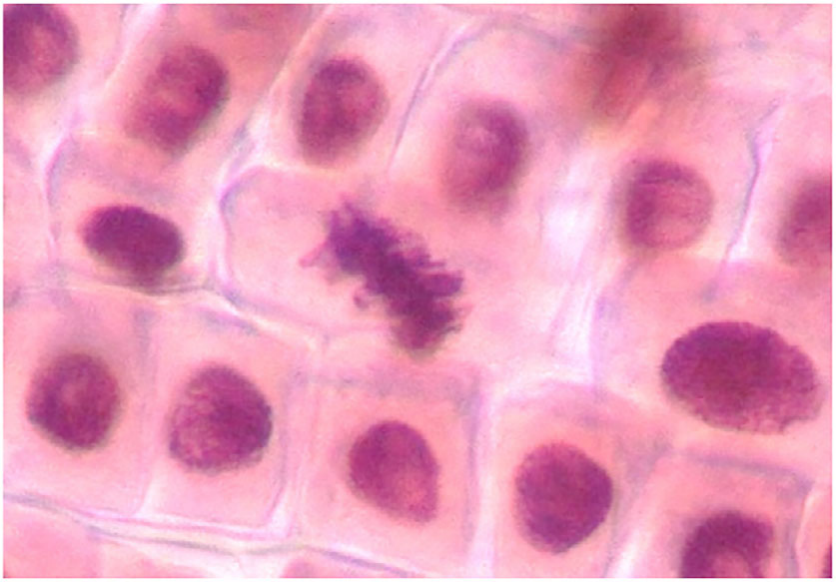
Metaphase in tropokinesis at C_3_ concentration of ligand **HL** ^**2**^.

**FIGURE 14:**
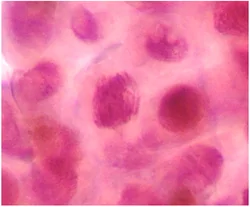
Anaphase in tropokinesis at C_1_ concentration of complex **1**.

**FIGURE 15:**
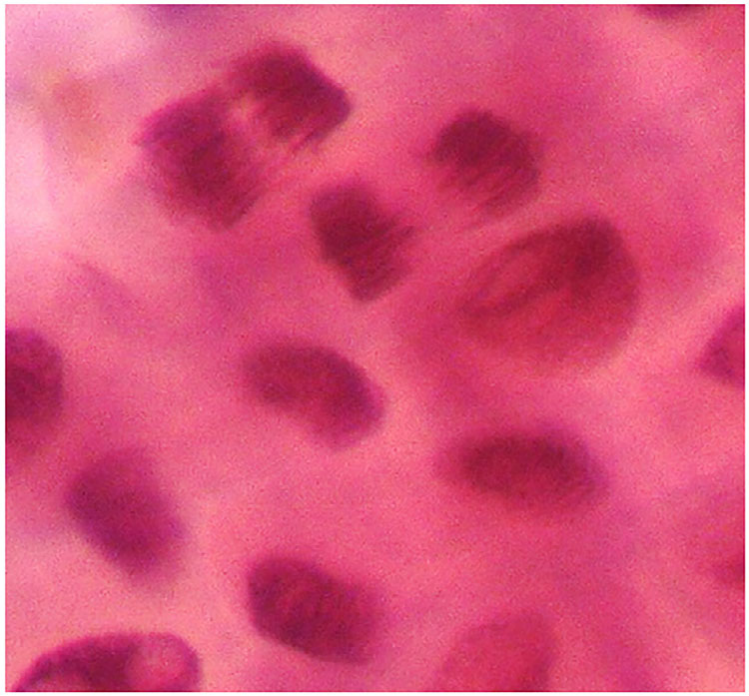
Anaphases and telophases in tropokinesis at C_1_ concentration of complex **3**.

**FIGURE 16:**
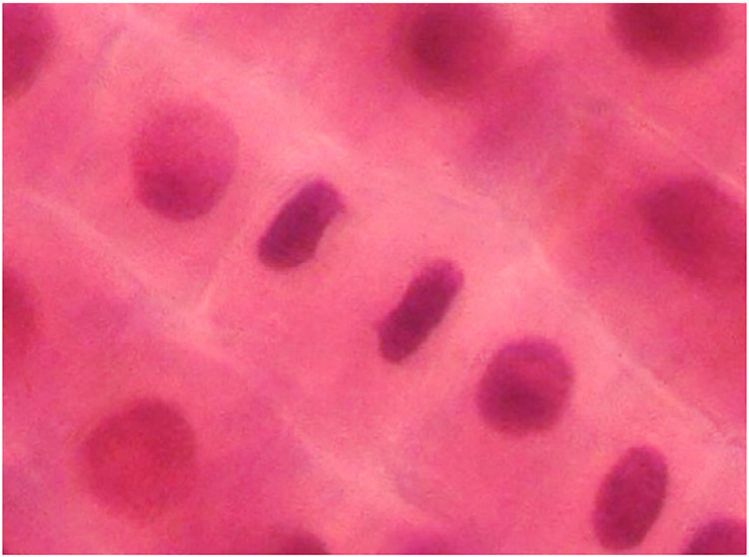
Telophase in slight tropokinesis at C_1_ concentration of ligand **HL** ^**4**^.

Additionally, anaphases with lagging chromosomes were observed for ligands **HL** ^**2**^ (Figure 17) and **HL** ^**3**^ (Figure S26), and telophases with lagging chromosomes were identified for ligand **HL** ^**1**^ (Figure 18).

**FIGURE 17:**
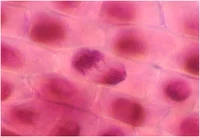
Anaphase with lagging chromosomes at C_1_ concentration of ligand **HL** ^**2**^.

**FIGURE 18:**
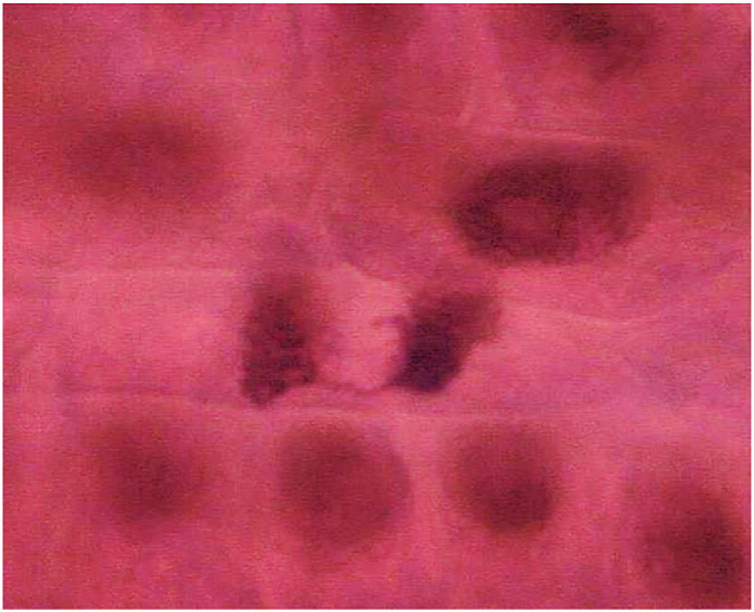
Telophase with lagging chromosomes at C_2_ concentration of ligand **HL** ^**1**^.

For ligand **HL** ^**2**^ (Figure S27), complex **2** (Figure 19), complex **3** (Figure S28), and complex **4** (Figure S29), rare areas with very limited mitotic activity were also observed.

**FIGURE 19:**
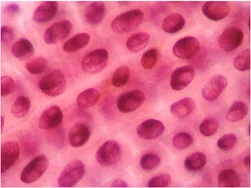
Area with rare cell division phases at C_1_ concentration of complex **2**.

Interphase cells with nuclei exhibiting hypertrophied nucleoli were observed in samples treated with **HL** ^**2**^ (Figure S30), **HL** ^**4**^ (Figure 20), and complex **3** (Figure S31).

**FIGURE 20:**
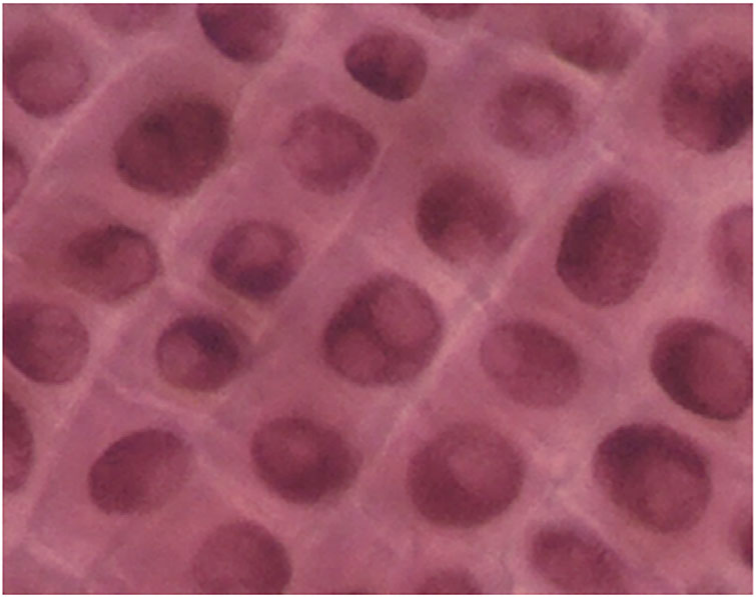
Interphase cells with nuclei showing slight hypertrophied nucleoli at C_3_ concentration of ligand **HL** ^**4**^.

### 3.6 Animal Toxicity Evaluation

All tested compounds were nonlethal to *A. franciscana* nauplii following 24 h of exposure at concentrations up to 100 µM. After 48 h of exposure, an increase in mortality was observed, but most ligands and complexes had low or very low mortality at all concentrations, and no clear concentration–lethality relationship was evident; therefore, an LC_50_ value could not be estimated. Higher mortality and clear concentration–lethality relationship were only observed for complexes **2** and **4**. LC_50_ values and their 95% confidence intervals are shown in Table 4. For reference, the literature reports LC_50_ values of 364 µM for cyclophosphamide and 10.2 µM for etoposide, two well‐known antineoplastic agents [112].

**TABLE 4:** LC_50_ values (95% confidence intervals) for *Artemia franciscana* Kellogg lethality test for complexes **2** and **4**.

Compound	LC_50_, μM	Std. error	95% confidence interval
Complex **2**	389.8	4922.488	−9258.1–10 037.7^a^
Complex **4**	48.7	32.910	−15.8–113.2^a^

a Because concentration cannot be negative, the lower bound is biologically constrained to zero. However, the extreme width of the interval indicates that the LC_50_ is highly uncertain based on the current data, particularly for complex **2**.

A graphical representation of the data and fitting lines for complexes **2** and **4** is shown in Figure 21.

**FIGURE 21:**
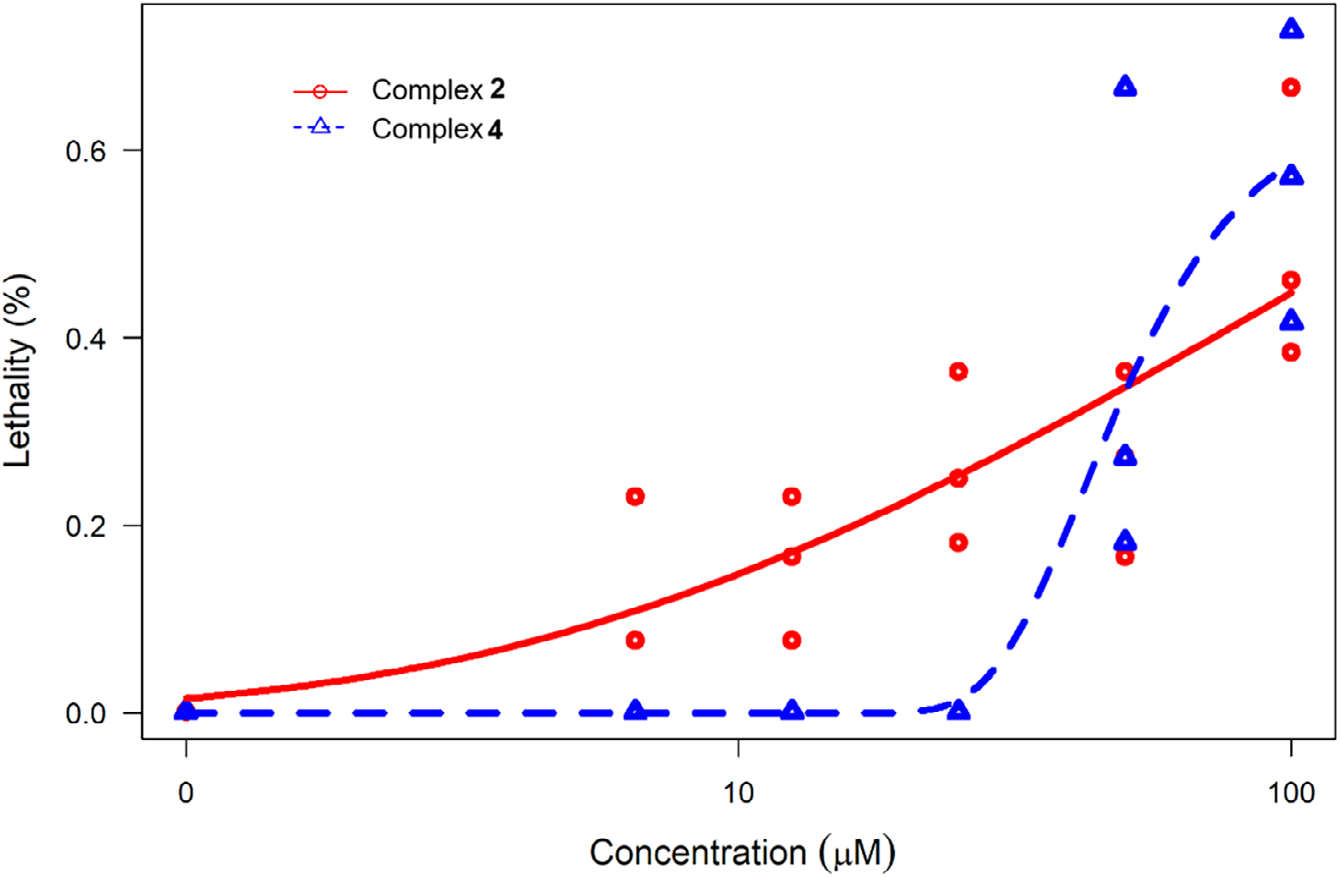
Concentration–lethality relationship for complexes **2** and **4**, showing observed lethality (%) across concentrations (μM) with fitted dose–response curves using the three‐parameter Weibull model.

For the remaining compounds, mortality did not exceed 38.5% even at the highest tested concentration of 100 µM.

## 4 Conclusion

Four new ligands derived from 2‐chloro‐5‐(trifluoromethyl)aniline and the corresponding palladium(II) complexes were obtained and characterized through elemental and spectral analyses. Additionally, the molecular and crystal architecture for all ligands and complexes **2** and **4** were elucidated through single‐crystal X‐ray diffraction technique. Ligands **HL** ^**1**^, **HL** ^**3**^, **HL** ^**4**^, and complex **4** crystallize in the monoclinic system, while **HL** ^**2**^ and complex **2** crystallize in the orthorhombic system. The ligands function as mononegative bidentate donors coordinating to the palladium ion through NO donor sets. Complexes **1**, **3**, and **4** are mononuclear species, containing a single palladium center, while complex **2** is dinuclear with two palladium ions linked via bidentate acetate bridges. In all cases, the palladium centers exhibit tetra‐coordinated, slightly distorted square‐planar geometry.

The tested complexes tended to inhibit root growth more strongly than the free ligands, although their overall inhibitory effects were modest. At lower concentrations (0.1–50 μM), a slight stimulatory effect on growth was observed, with the strongest stimulation at 50 μM, while the highest concentration (100 μM) produced only a modest increase. Overall, growth increased over time, particularly from day 2 to day 3, across most concentrations and variables.

Most ligands and complexes had low or very low mortality to *A. franciscana* nauplii at all tested concentrations, only complexes **2** and **4** exhibiting dose‐dependent toxicity.

These findings provide a basis for further exploration of the biological potential of the newly synthesized compounds.

## Author Contributions

**Andreea Elena Ştefan**: conceptualization, investigation, methodology, formal analysis, writing – original draft, writing – review and editing. **Cristina Elena Dinu Pîrvu**: funding acquisition, investigation, methodology, validation, resources, writing – review and editing. **Sergiu Shova**, **Ştefania Felicia Bărbuceanu**, **Teodora Venera Apostol**, **Diana Carolina Ilieş**, **Mihaela Badea**, **Mihaela Dinu**, **Robert Viorel**: investigation, methodology, validation. **Doina Drăgănescu**: funding acquisition, investigation, methodology, validation, writing – review and editing. **Elena Pahonţu**: conceptualization, investigation, formal analysis, supervision, validation, writing – original draft, writing – review and editing, resources, project administration.

## Funding

This work was supported by the “Carol Davila” University of Medicine and Pharmacy, Bucharest, Romania, through the INOFORMDOC project (MySMIS Code 351225).

## Conflicts of Interest

The authors declare no conflicts of interest.

## Supporting information

Deposition numbers 2515661 (for **HL^1^**), 2515662 (for **HL^2^**), 2515663 (for **HL^3^**), 2515664 (for **HL^4^**), 2515665 (for complex **2**), and 2515666 (for complex **4**) contain the supporting crystallographic data for this paper. These data are provided free of charge by the joint Cambridge Crystallographic Data Centre and Fachinformationszentrum Karlsruhe Access Structures service.

## Data Availability

The data that support the findings of this study are available in the Supporting Information of this article.
