# Supplementary material for: 2‐Chloro‐5‐(trifluoromethyl)aniline‐Derived Schiff Base Palladium(II) Complexes: From Molecular Design to Acute Toxicity Assessment
Source: ChemistryOpen. 2026 Sep 30;15(10):e70315. doi: 10.1002/open.70315 (PMC13627978; doi:10.1002/open.70315)
Supplement: Supplementary file 1 [file OPEN-15-e70315-s001.pdf]

2-Chloro-5-(trifluoromethyl)aniline-Derived Schiff Base Palladium(II) Complexes: From Molecular Design to Acute Toxicity Assessment

Andreea Elena Ștefan[a] | Cristina Elena Dinu-Pîrvu*[b] | Sergiu Shova[c] | Ștefania Felicia Bărbuceanu[d] | Teodora Venera Apostol[d] | Diana Carolina Ilieș[a] | Mihaela Badea[e] | Mihaela Dinu[f] | Robert Viorel Ancuceanu[f] | Doina Drăgănescu*[g] | Elena Pahonțu*[a]

[a] General and Inorganic Chemistry Department, Faculty of Pharmacy, "Carol Davila" University of Medicine and Pharmacy, Bucharest, Romania.
[b] Physical and Colloidal Chemistry Department, Faculty of Pharmacy, "Carol Davila" University of Medicine and Pharmacy, Bucharest, Romania.
[c] Institute of Macromolecular Chemistry „Petru Poni", Iasi, Romania.
[d] Organic Chemistry Department, Faculty of Pharmacy, "Carol Davila" University of Medicine and Pharmacy, Bucharest, Romania.
[e] Inorganic Chemistry Department, Faculty of Chemistry, University of Bucharest, Bucharest, Romania,
[f] Pharmaceutical Botany and Cell Biology Department, Faculty of Pharmacy, "Carol Davila" University of Medicine and Pharmacy, Bucharest, Romania.
[g] Pharmaceutical Physics Department, Faculty of Pharmacy, "Carol Davila" University of Medicine and Pharmacy, Bucharest, Romania.

Table of Contents

Figure S1. IR spectrum of ligand **HL^1** 3
Figure S2. IR spectrum of ligand **HL^2** 3
Figure S3. IR spectrum of ligand **HL^3** 4
Figure S4. IR spectrum of complex **HL^4** 4
Figure S5. IR spectrum of complex **1** 5
Figure S6. IR spectrum of complex **2.** 5
Figure S7. IR spectrum of complex **3.** 6
Figure S8. IR spectrum of complex **4.** 6
Figure S9. ^{1}H NMR spectrum of ligand **HL^1** 7
Figure S10. ^{13}C NMR spectrum of ligand **HL^1** 7
Figure S11. ^{1}H NMR spectrum of ligand **HL^2** 8
Figure S12. ^{13}C NMR spectrum of ligand **HL^2** 8
Figure S13. ^{1}H NMR spectrum of ligand **HL^3** 9
Figure S14. ^{1}H NMR spectrum of ligand **HL^4** 9
Figure S15. ^{13}C NMR spectrum of ligand **HL^4** 10
Figure S16. Electronic spectra of the ligand **HL^1** and complex **1** 10
Figure S17. Electronic spectra of the ligand **HL^2** and complex **2** 11
Figure S18. Electronic spectra of the ligand **HL^3** and complex **3** 11
Figure S19. Electronic spectra of the ligand **HL^4** and complex **4** 12
Figure S20. Mass spectrum of complex **1** 12
Figure S21. Mass spectrum of complex **3** 13
Figure S22. Thermogram of complex **3** 13
Figure S23. Thermogram of complex **4** 14
Figure S24. Anaphase in tropokinesis at C_3 concentration of ligand **HL^4** 14
Figure S25. Telophase in tropokinesis at C_5 concentration of ligand **HL^1** 14
Figure S26. Anaphase with lagging chromosomes at C_1 concentration of ligand **HL^3** 14
Figure S27. Area with rare cell division phases at C_1 concentration of ligand **HL^2** 15

Figure S28. Area with rare cell division phases at C_1 concentration of complex **3** **15**
Figure S29. Area with rare cell division phases at C3 concentration of complex **4** **15**
Figure S30. Interphase cells with nuclei showing slight hypertrophied nucleoli at C_1 concentration of ligand $\mathbf{HL^2}$ **15**
Figure S31. Interphase cells with nuclei showing slight hypertrophied nucleoli at C_1 concentration of complex **3** **16**
Table S1. Bond distances (Å) and angles (°).................. **16**
Table S2. Mass spectral data of complexes **1** and **3** **19**

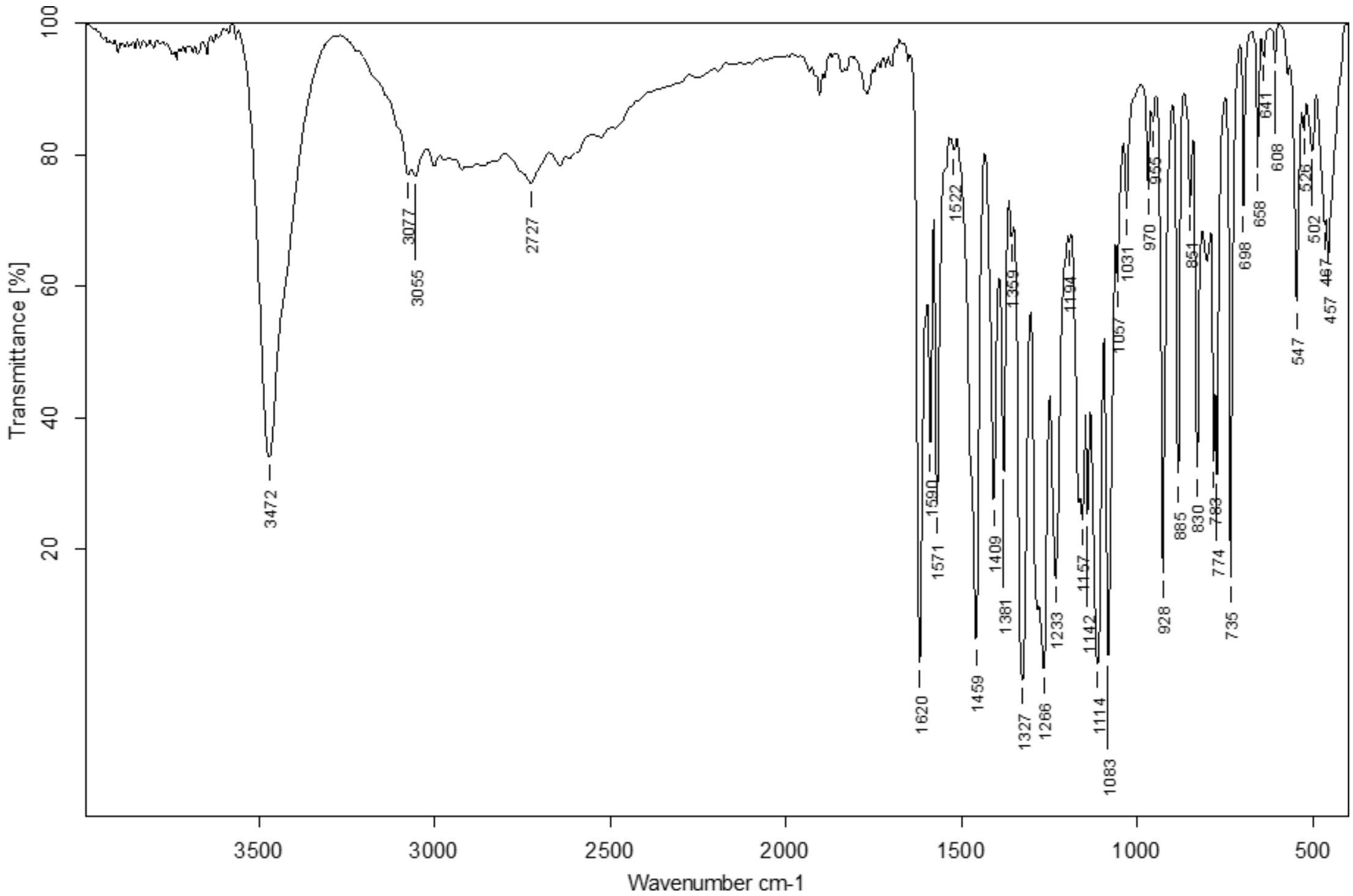

Figure S1. IR spectrum of ligand **HL**[1].

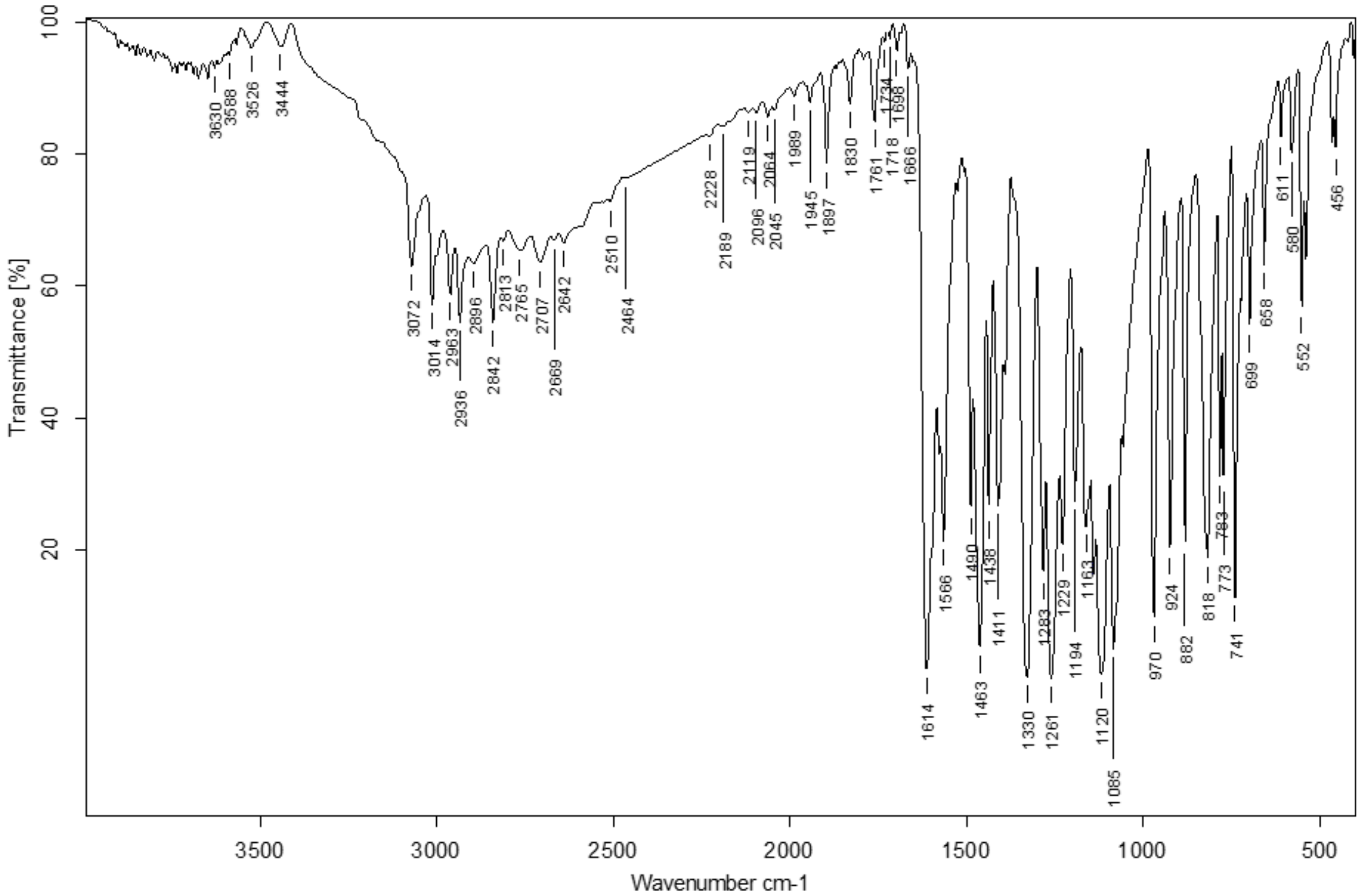

Figure S2. IR spectrum of ligand **HL**[2].

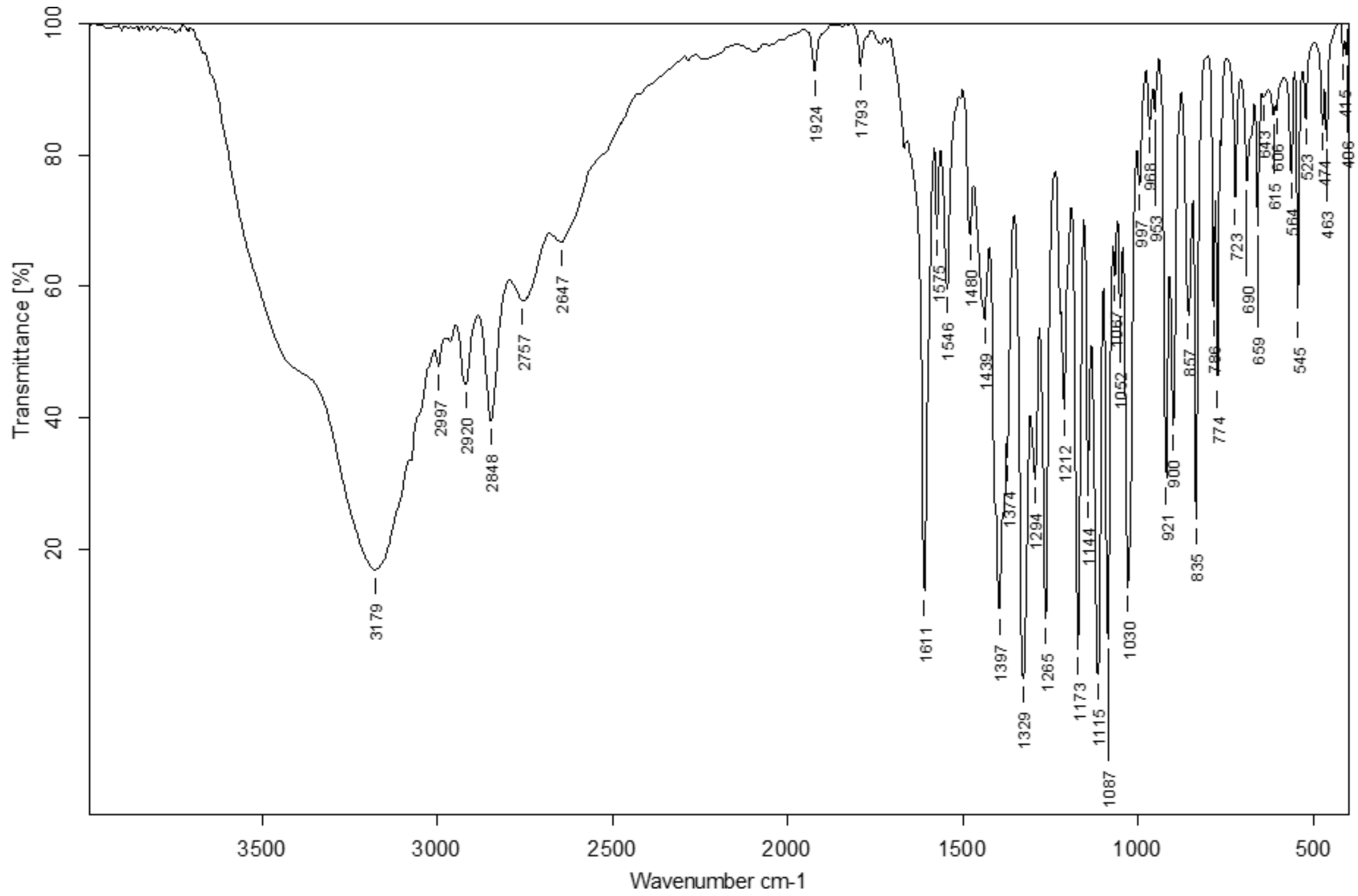

Figure S3. IR spectrum of ligand **HL[3]**.

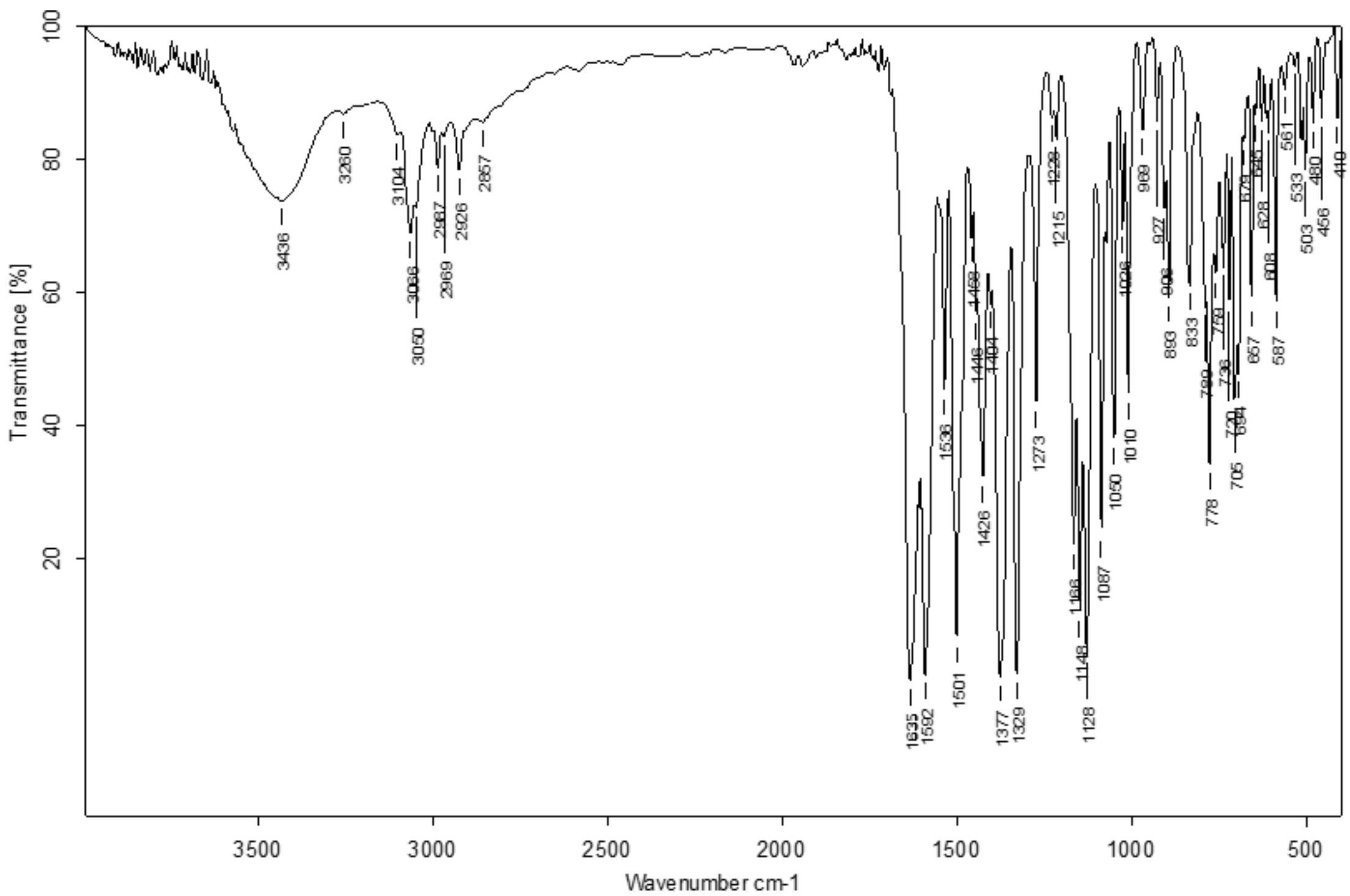

Figure S4. IR spectrum of complex **HL[4]**.

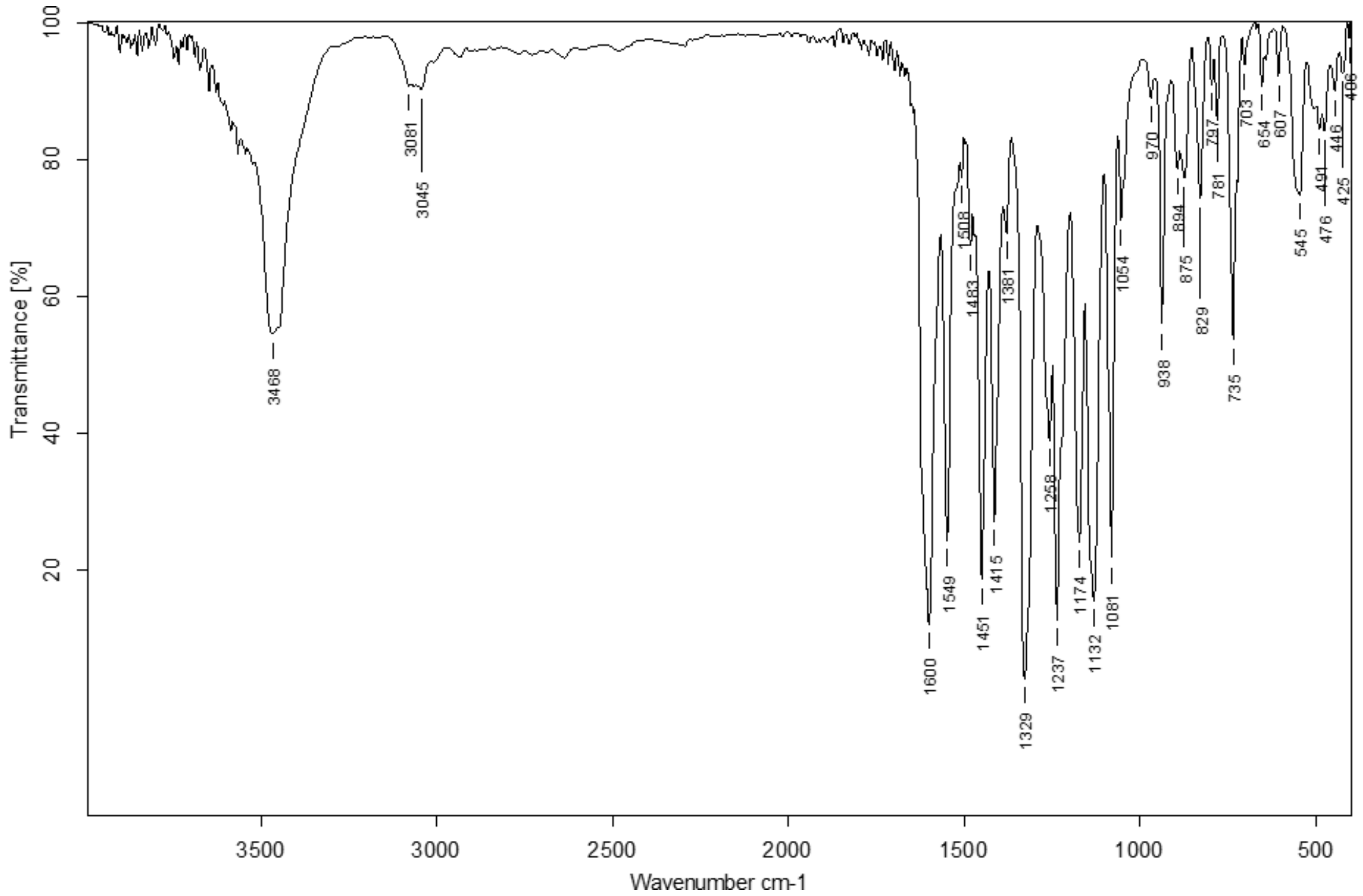

Figure S5. IR spectrum of complex **1**.

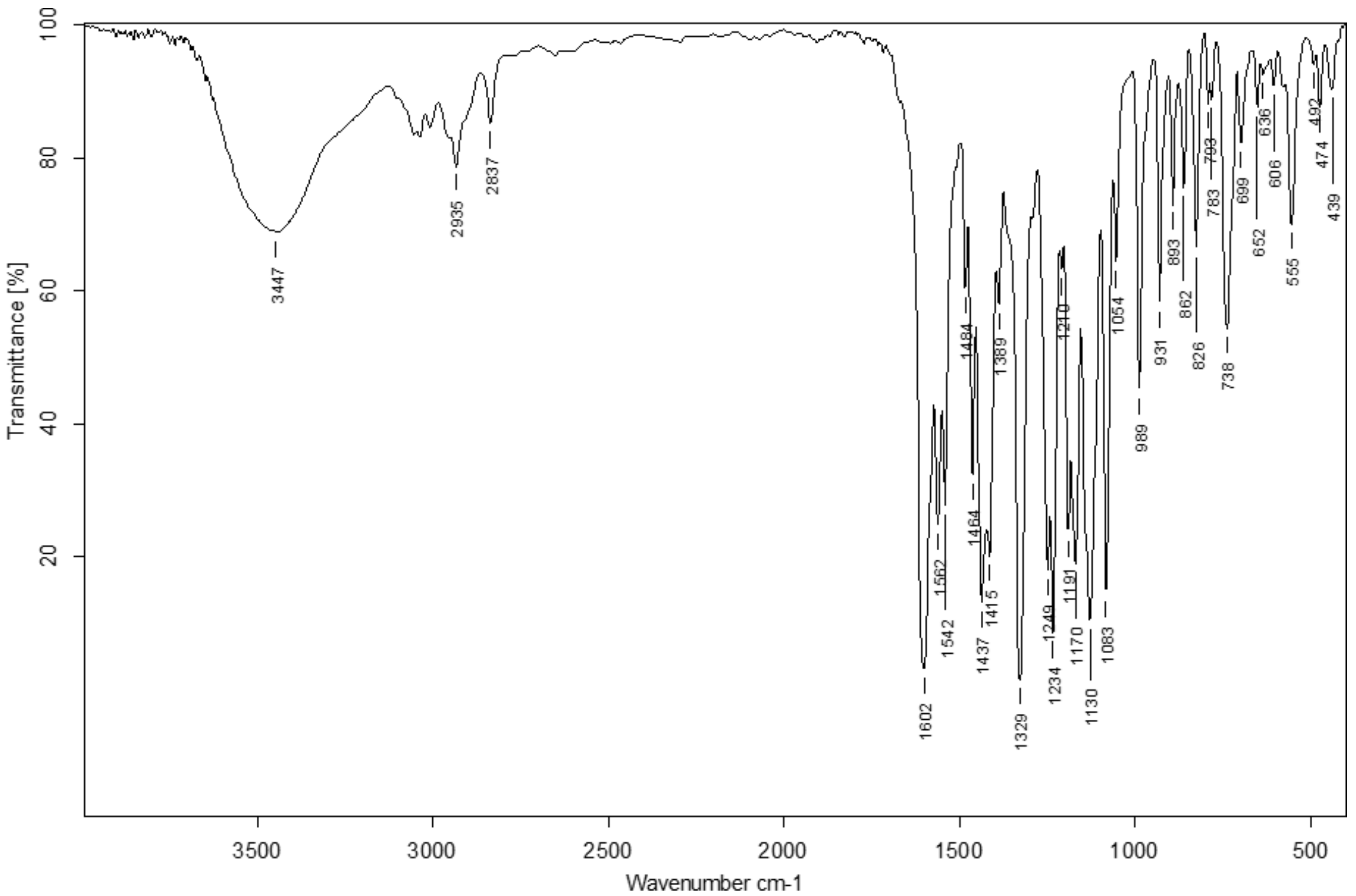

Figure S6. IR spectrum of complex **2**.

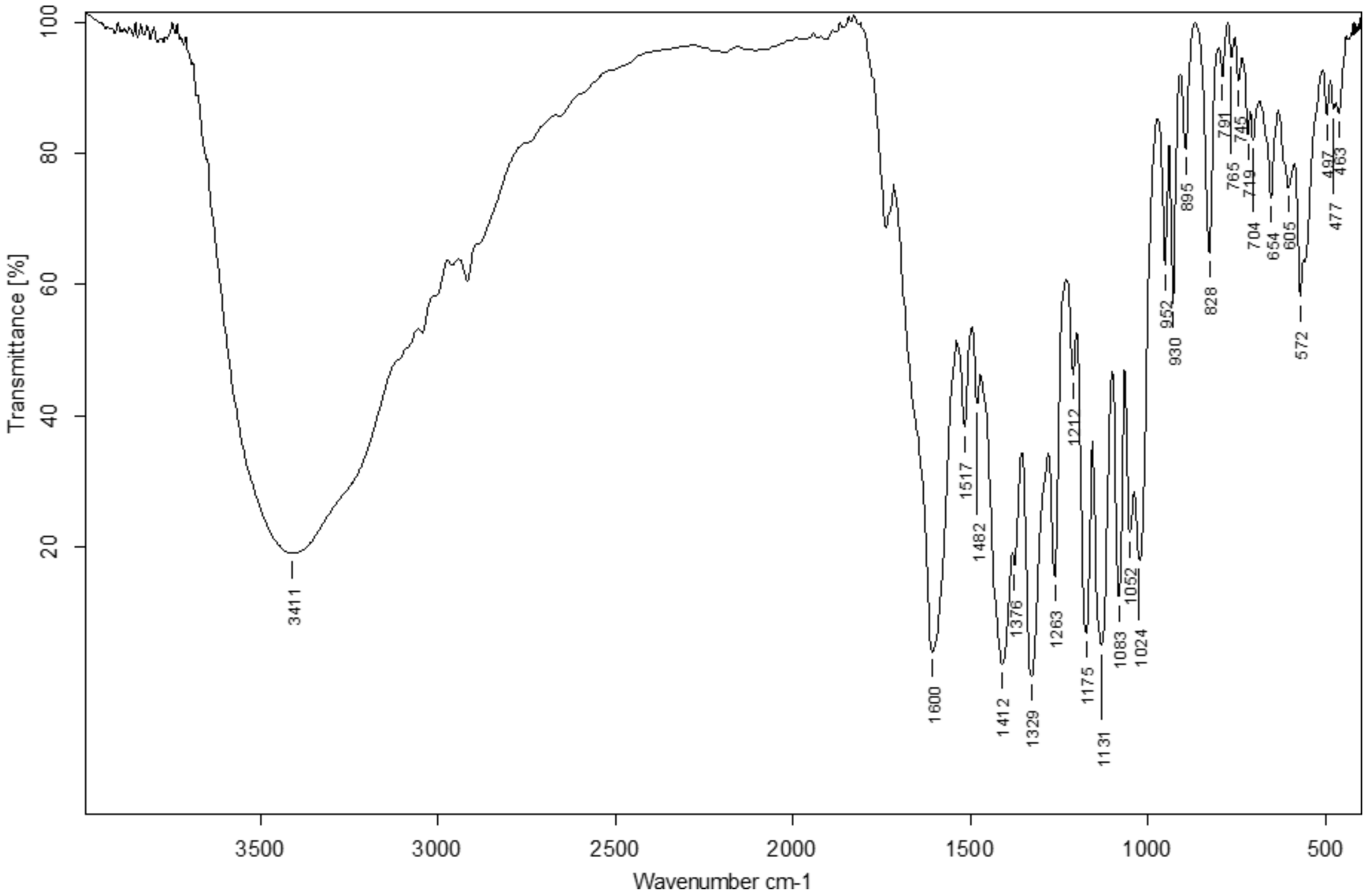

Figure S7. IR spectrum of complex **3**.

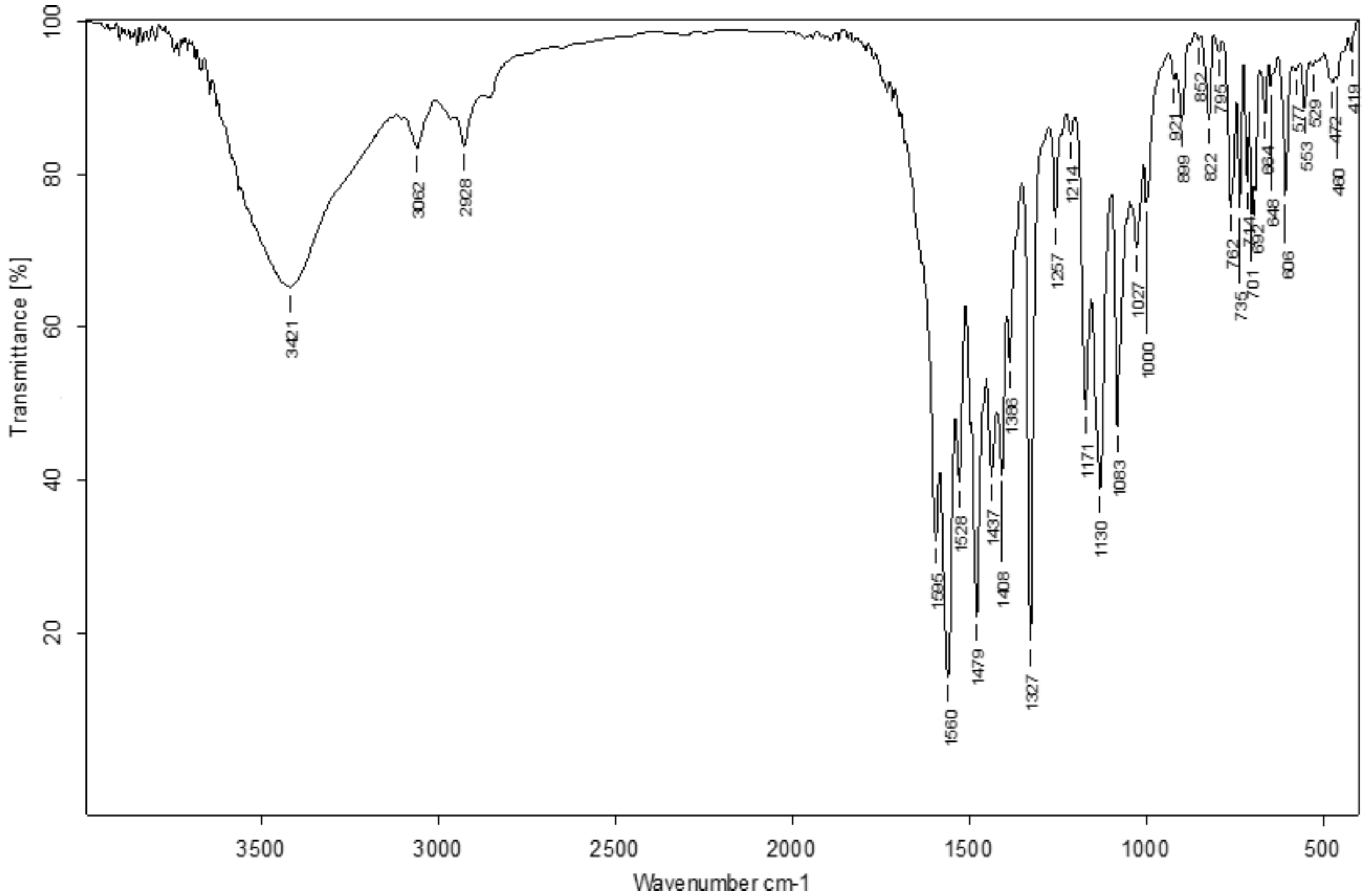

Figure S8. IR spectrum of complex **4**.

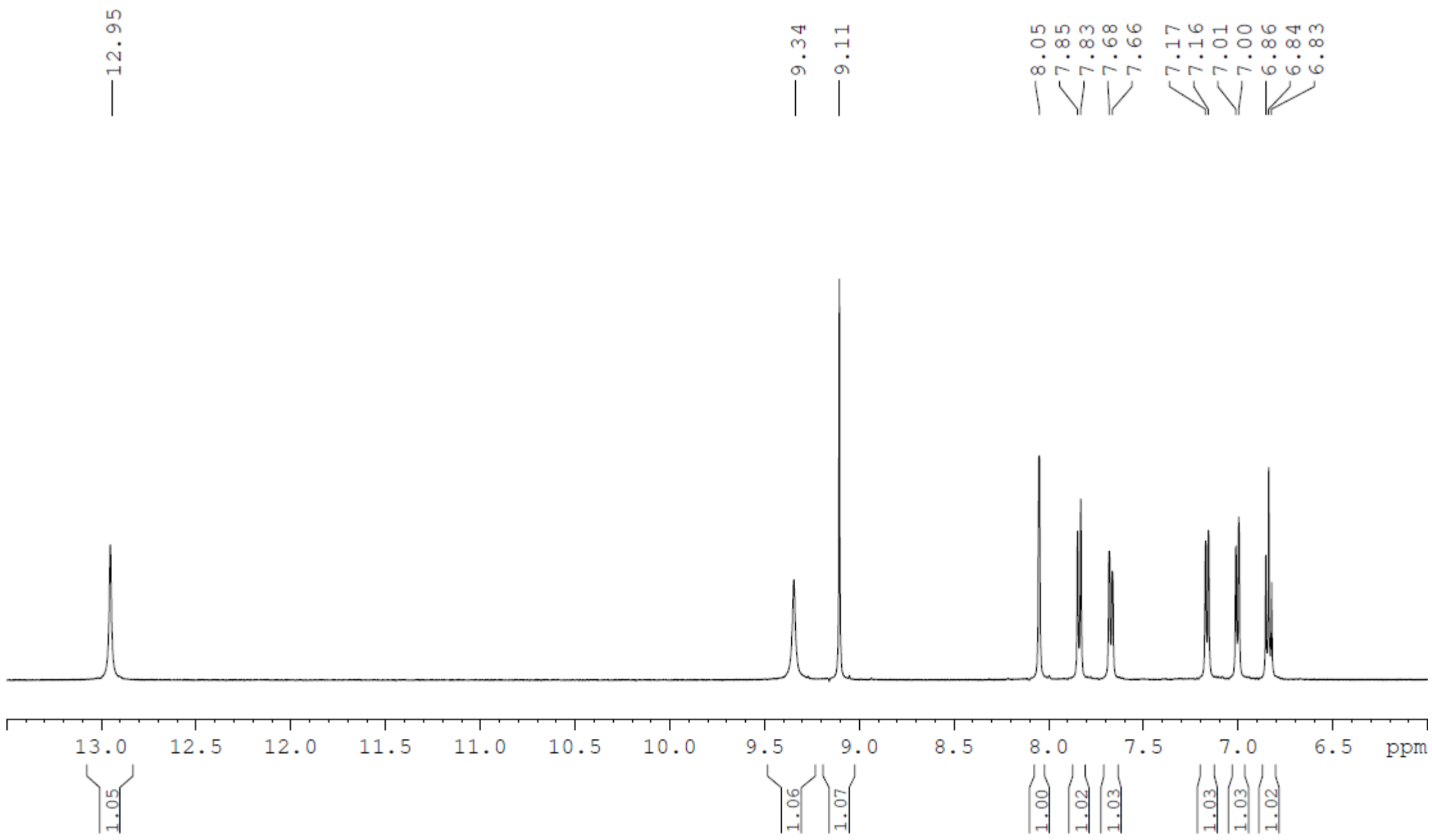

Figure S9. ^{1}H NMR spectrum of ligand **HL¹**.

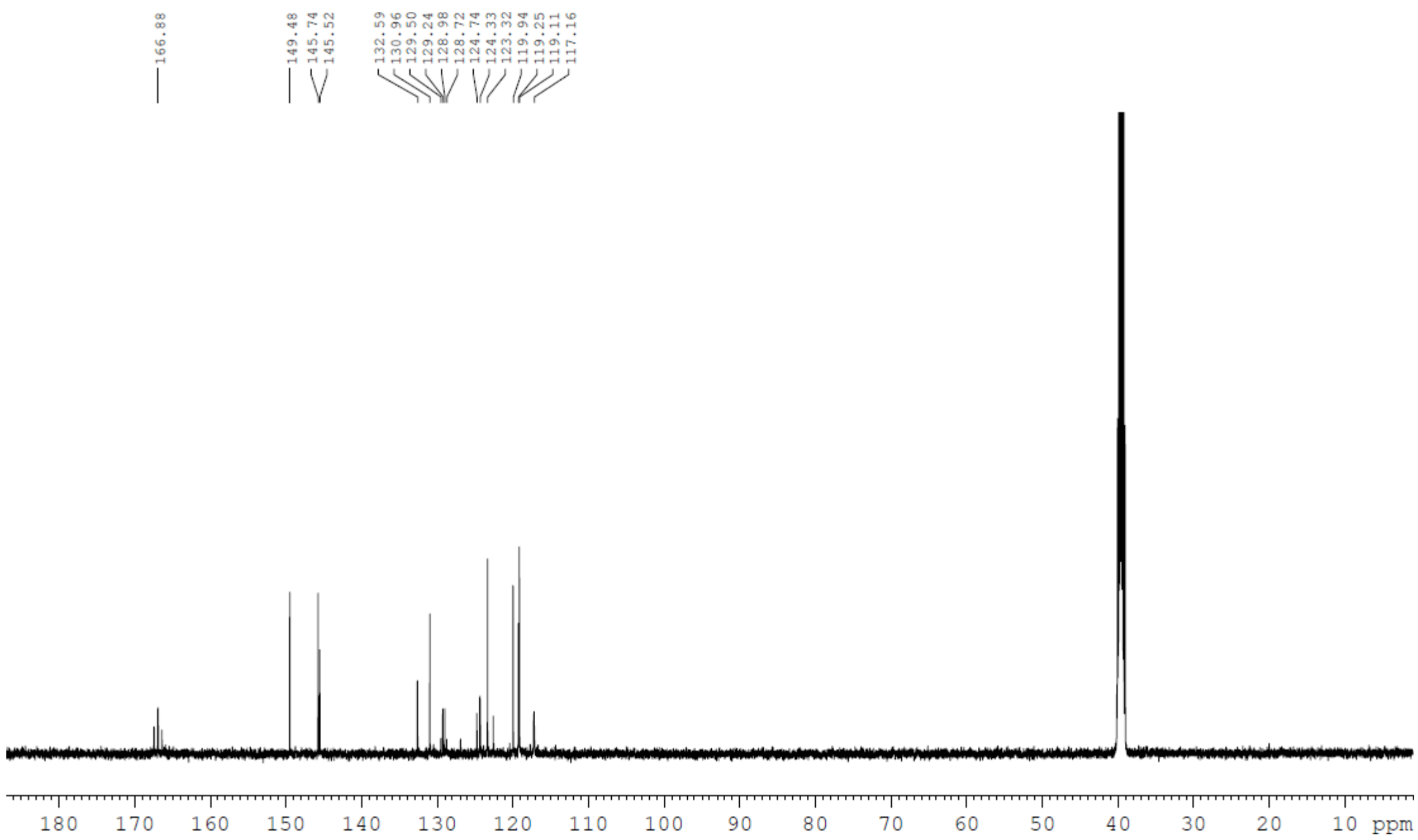

Figure S10. ^{13}C NMR spectrum of ligand **HL^{1}**.

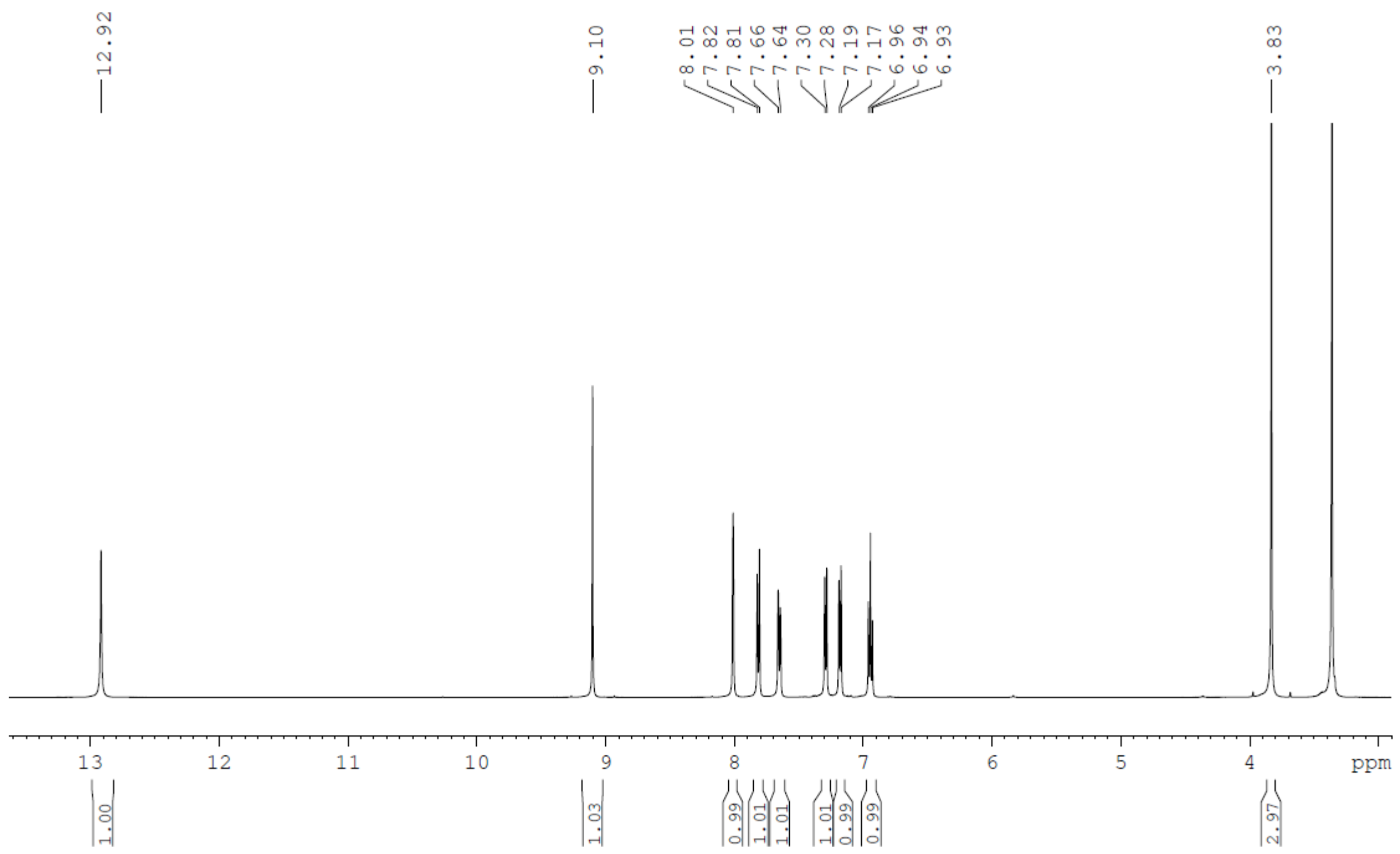

Figure S11. ^{1}H NMR spectrum of ligand $\mathbf{HL^2}$.

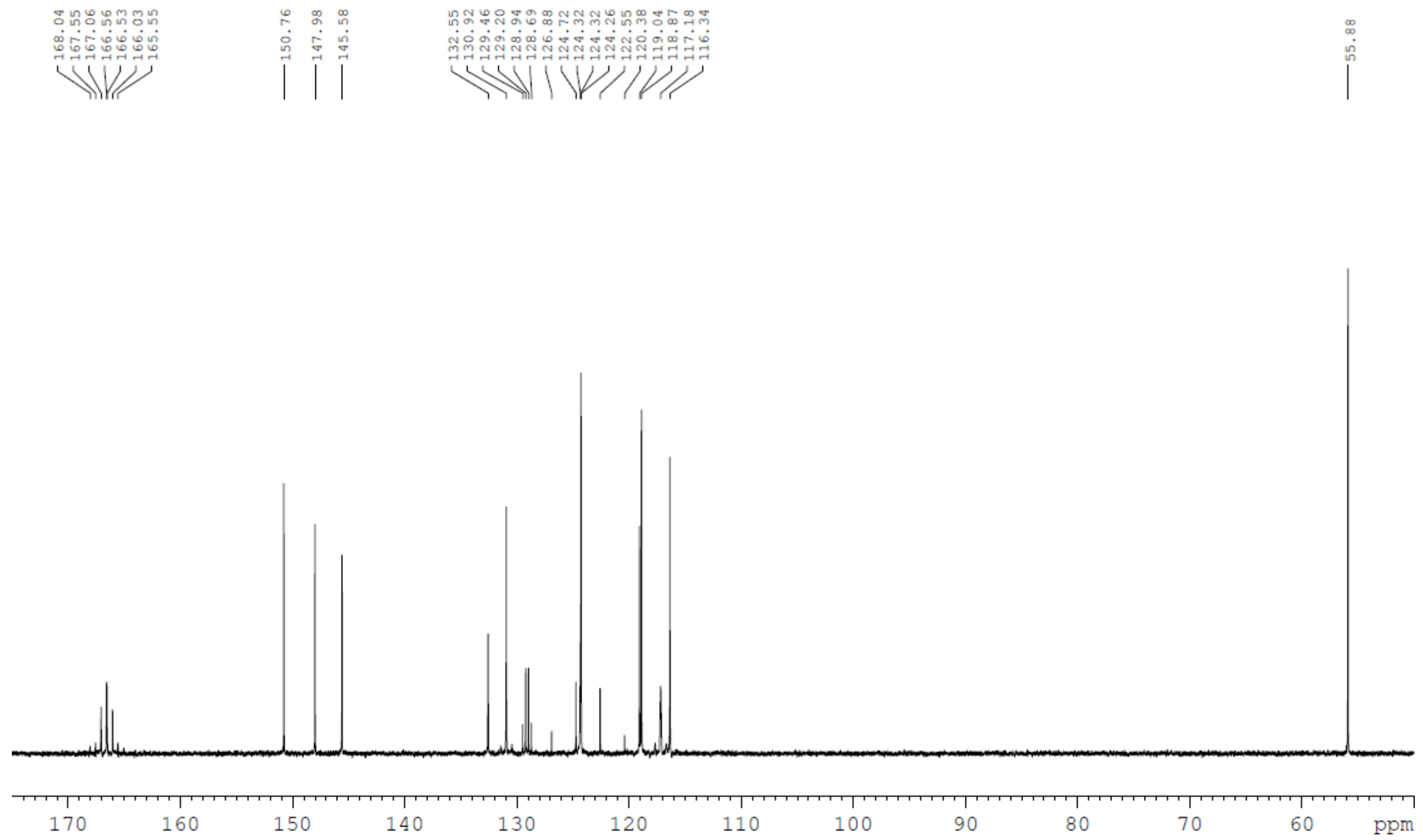

Figure S12. ^{13}C NMR spectrum of ligand $\mathbf{HL^2}$.

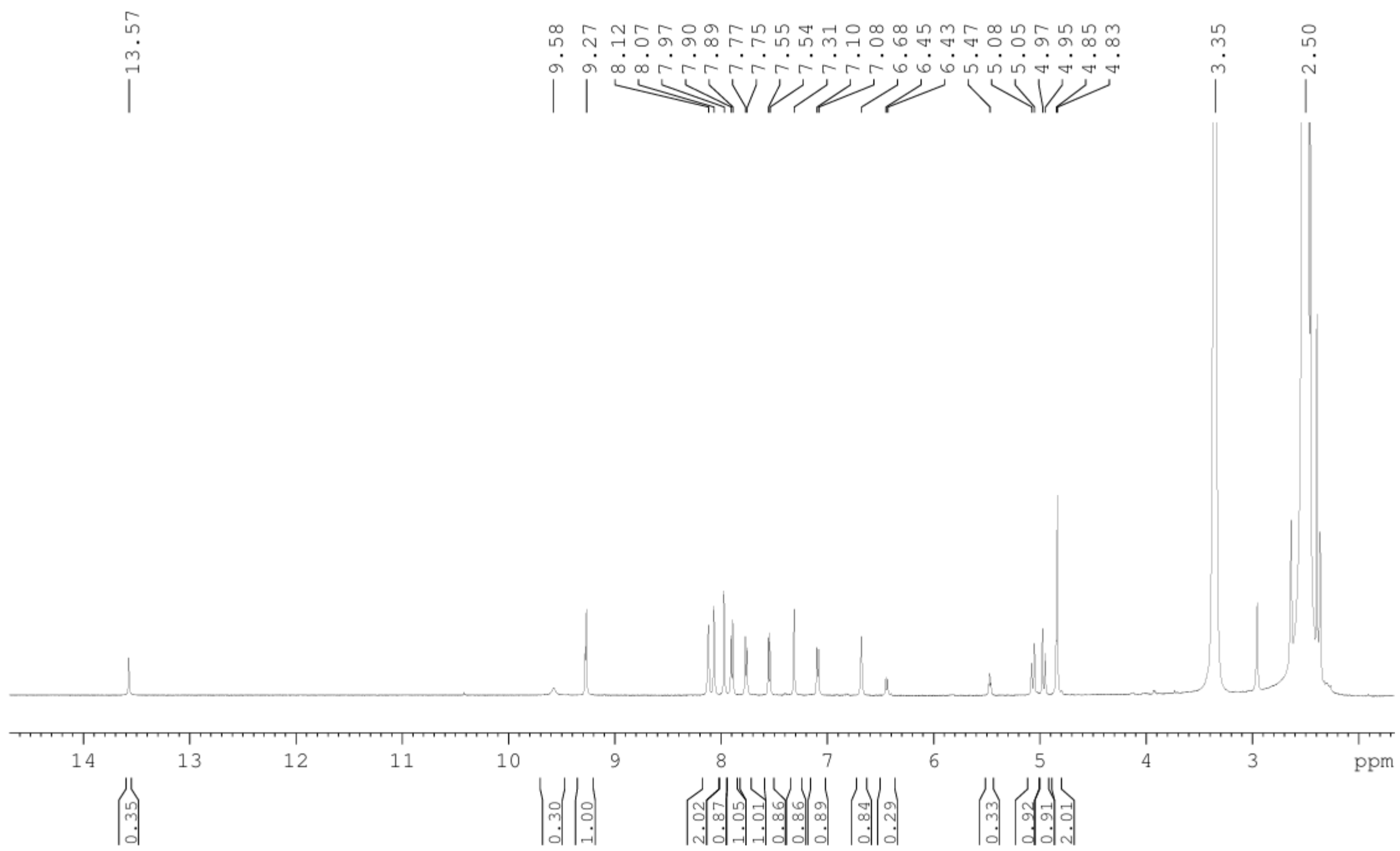

Figure S13. ^{1}H NMR spectrum of ligand **HL^{3}**.

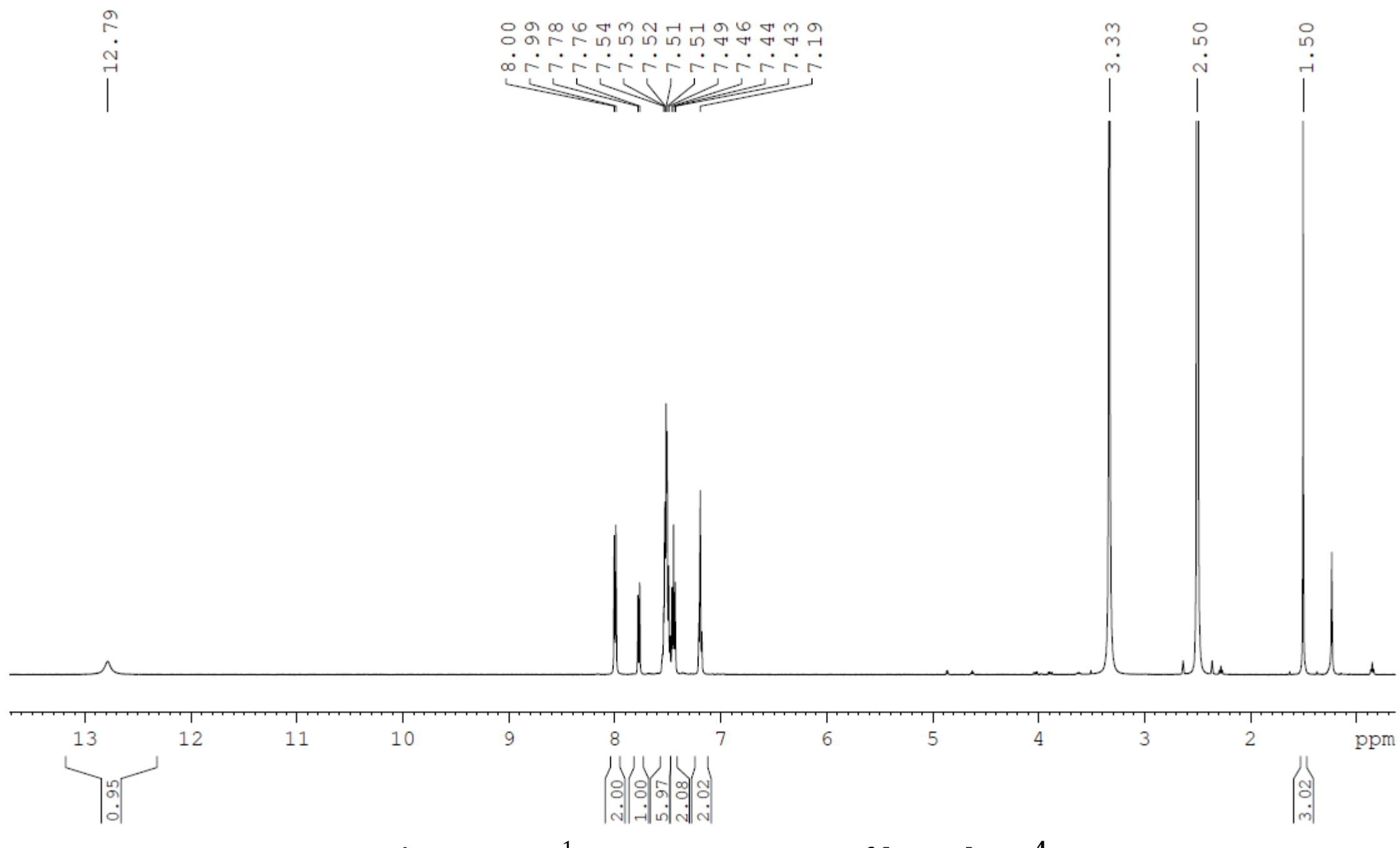

Figure S14. ^{1}H NMR spectrum of ligand **HL^{4}**.

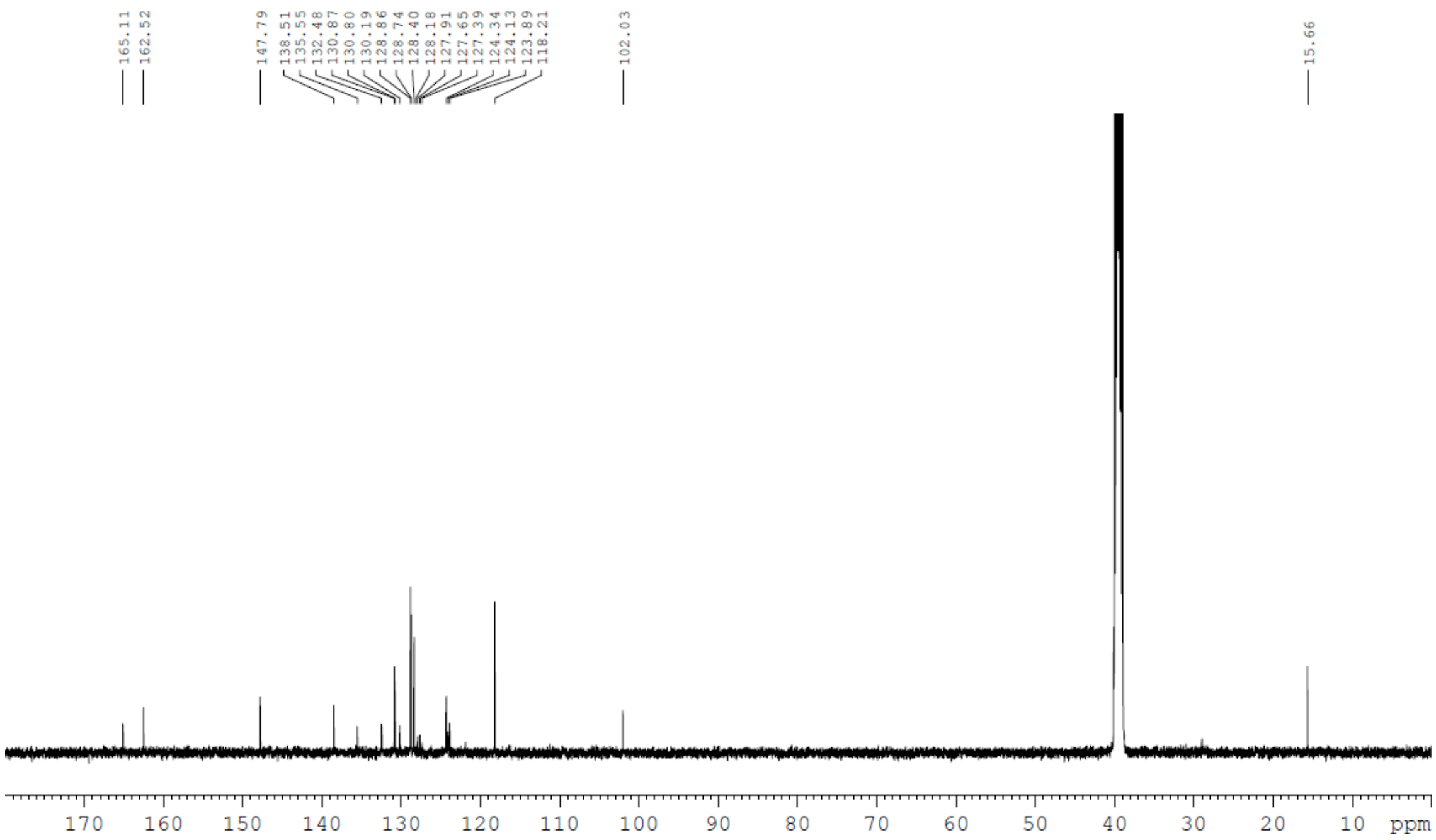

Figure S15. ^{13}C NMR spectrum of ligand **HL**[4].

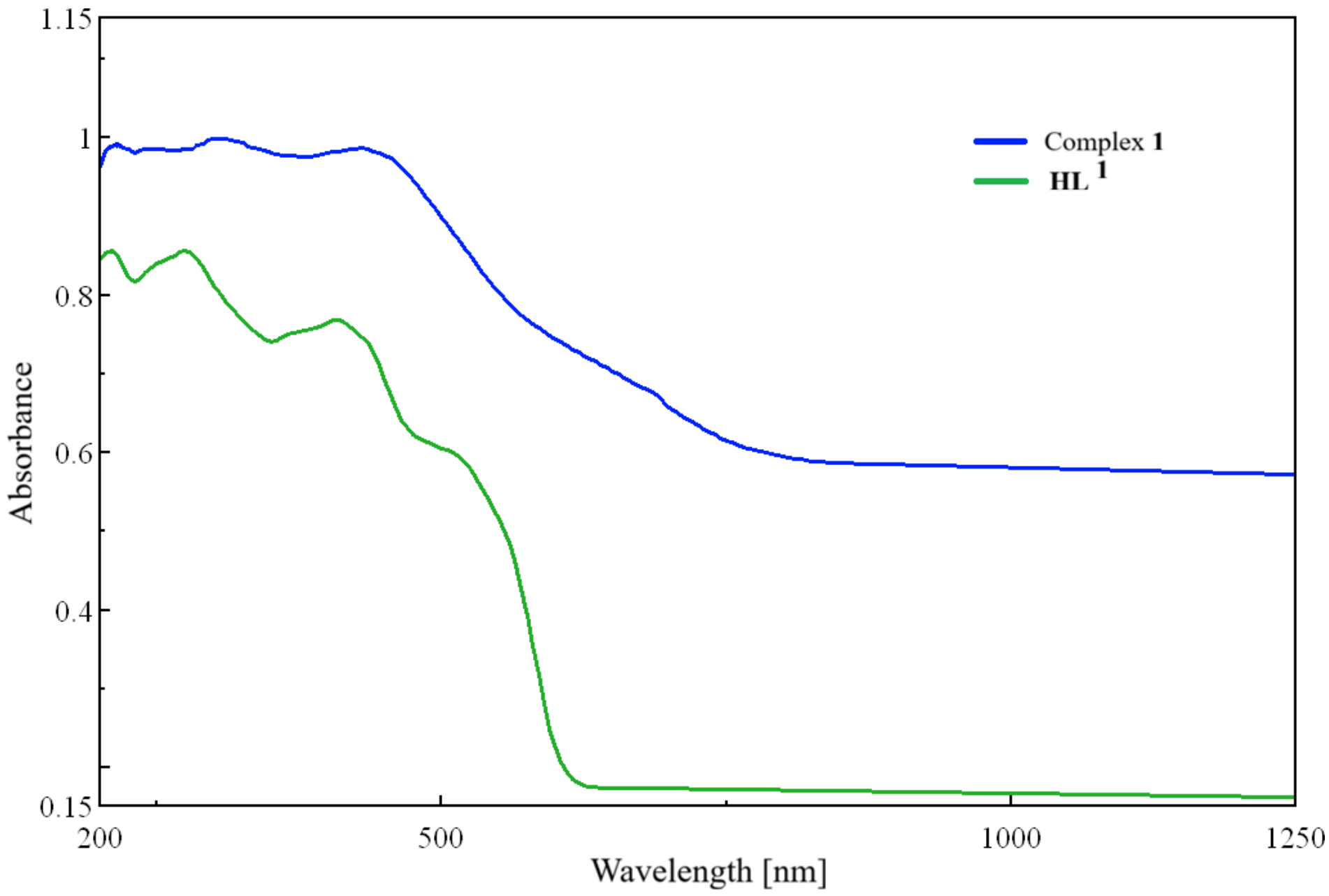

Figure S16. Electronic spectra of the ligand **HL**[1] and complex **1**.

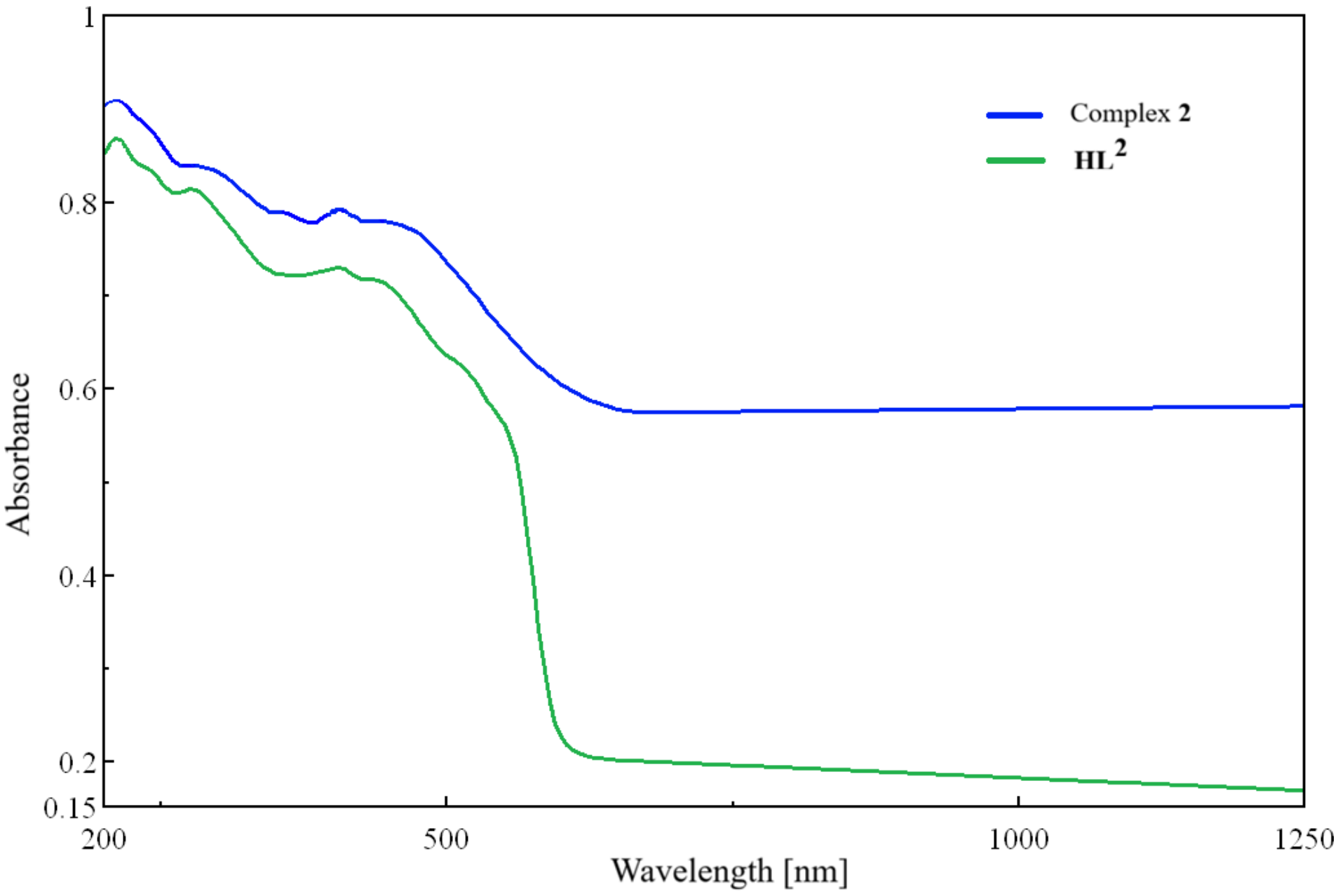

Figure S17. Electronic spectra of the ligand **HL**^{2} and complex **2**.

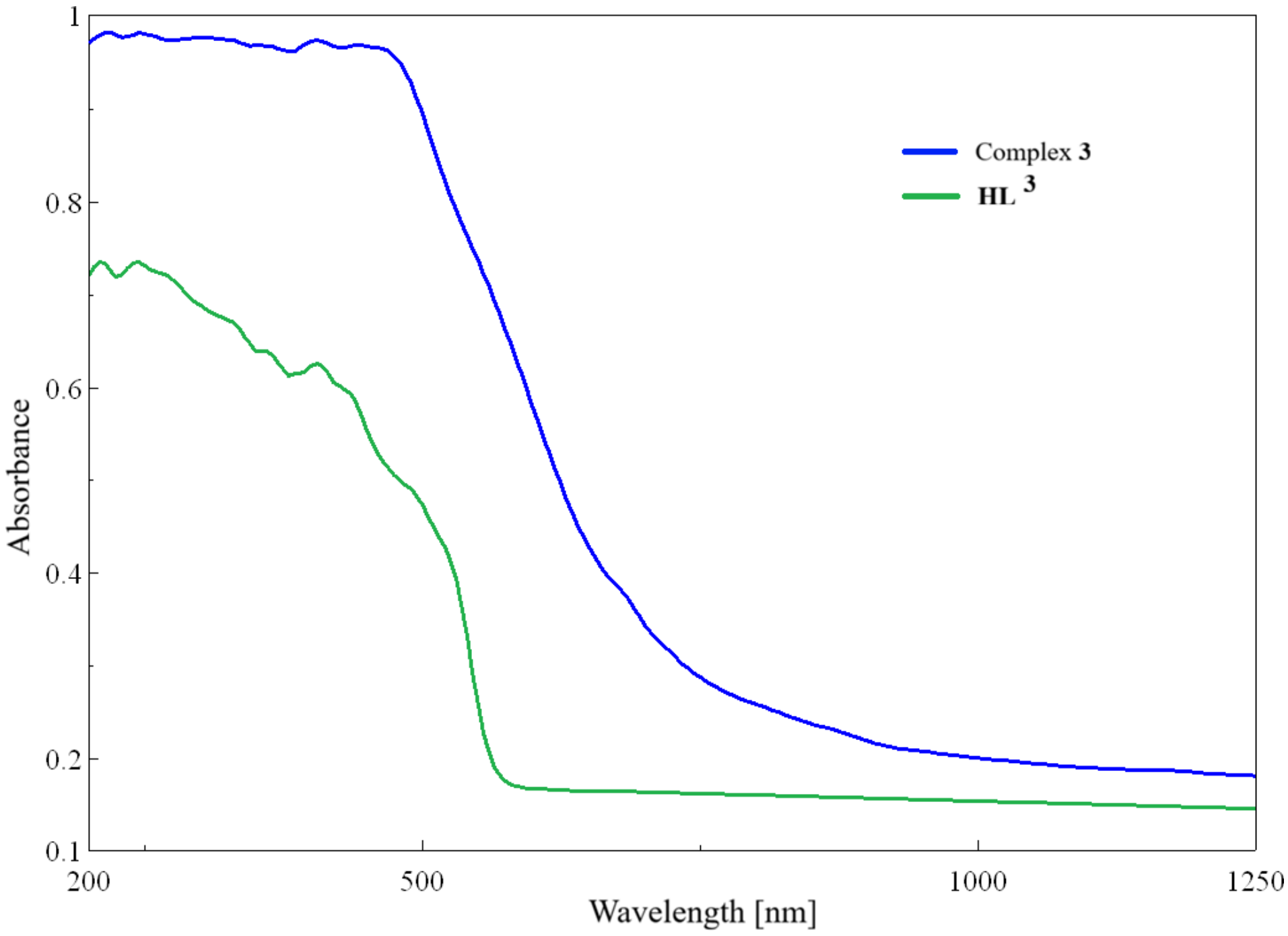

Figure S18. Electronic spectra of the ligand **HL**^{3} and complex **3**.

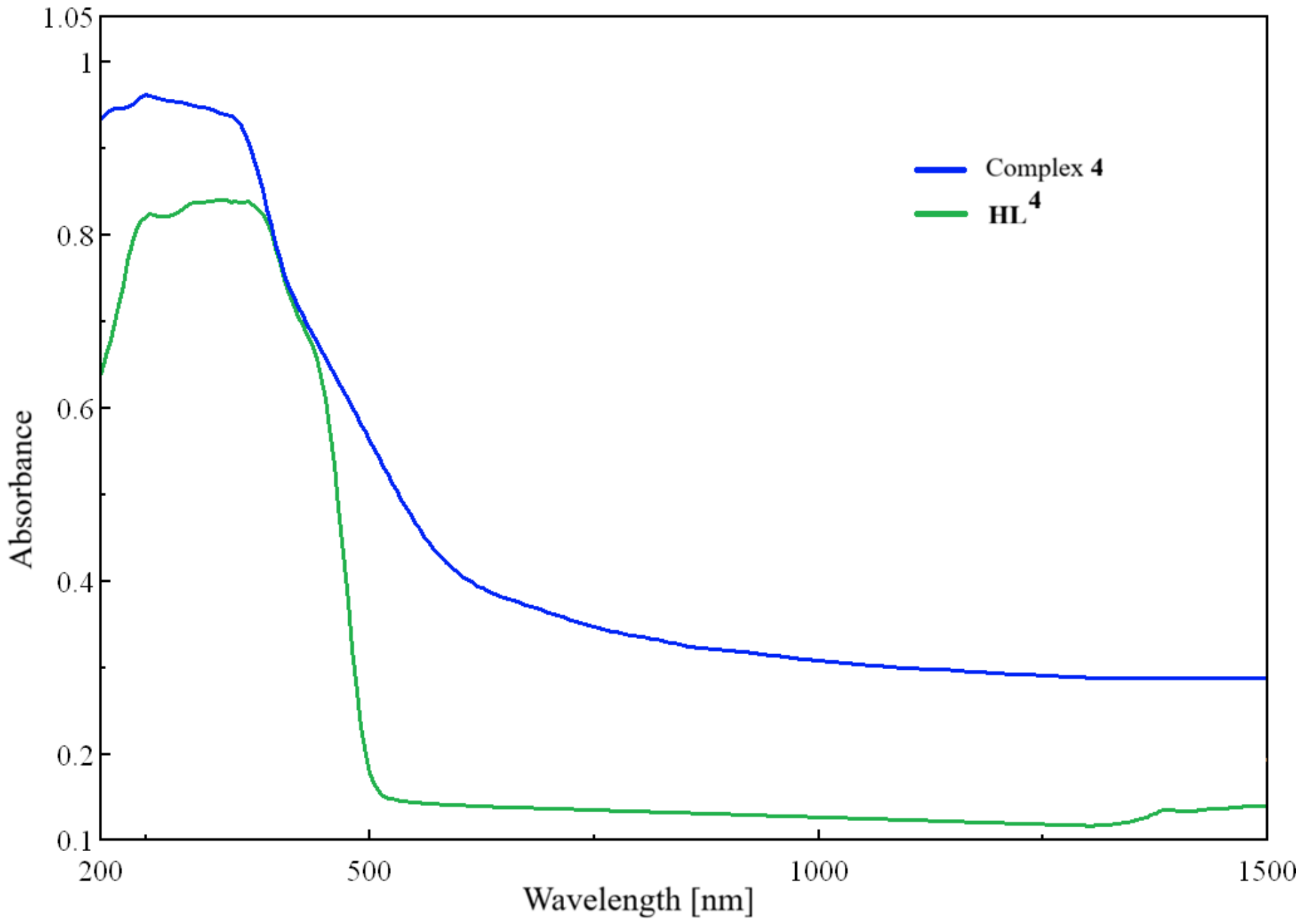

Figure S19. Electronic spectra of the ligand **HL**[4] and complex **4**.

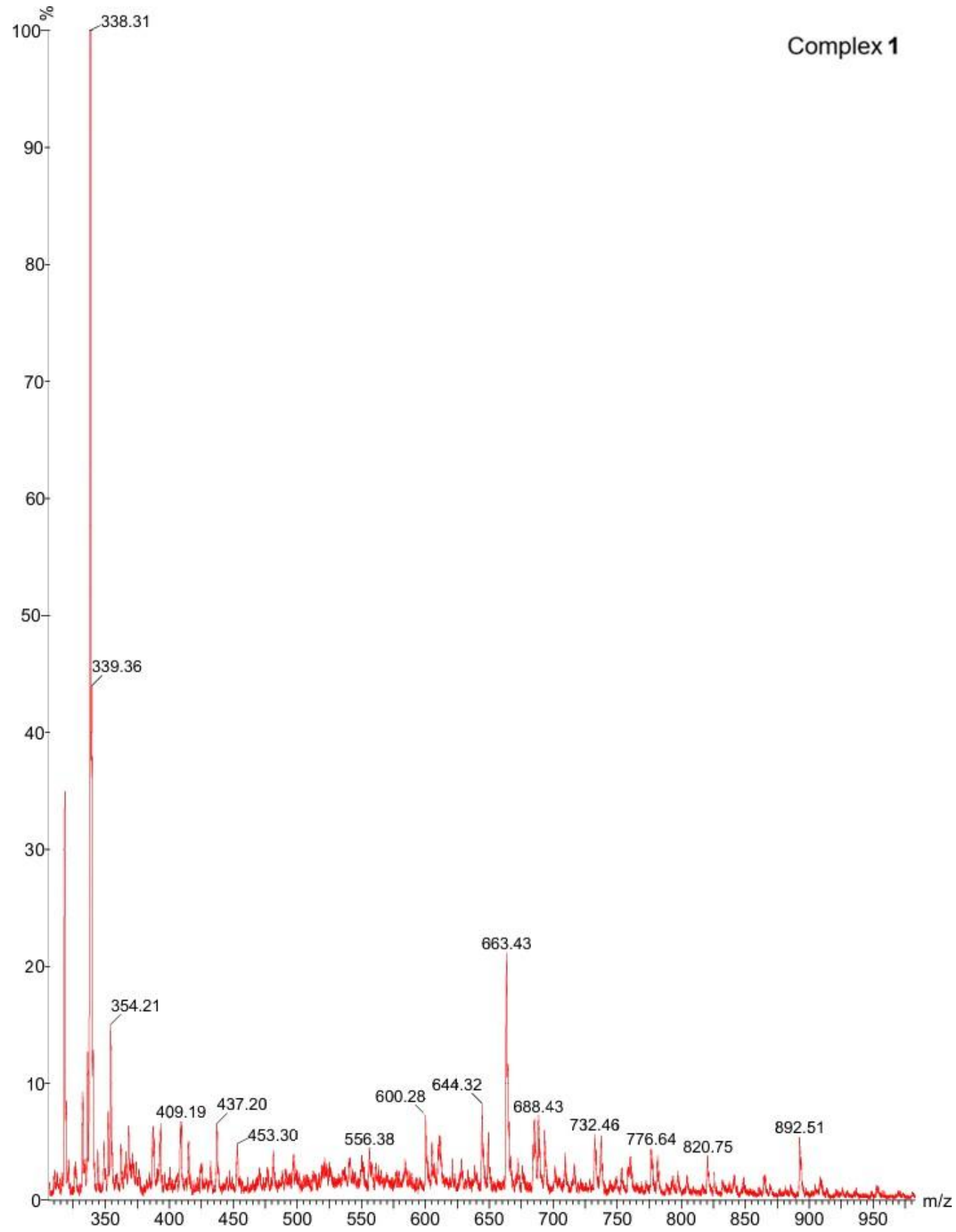

Figure S20. Mass spectrum of complex **1**.

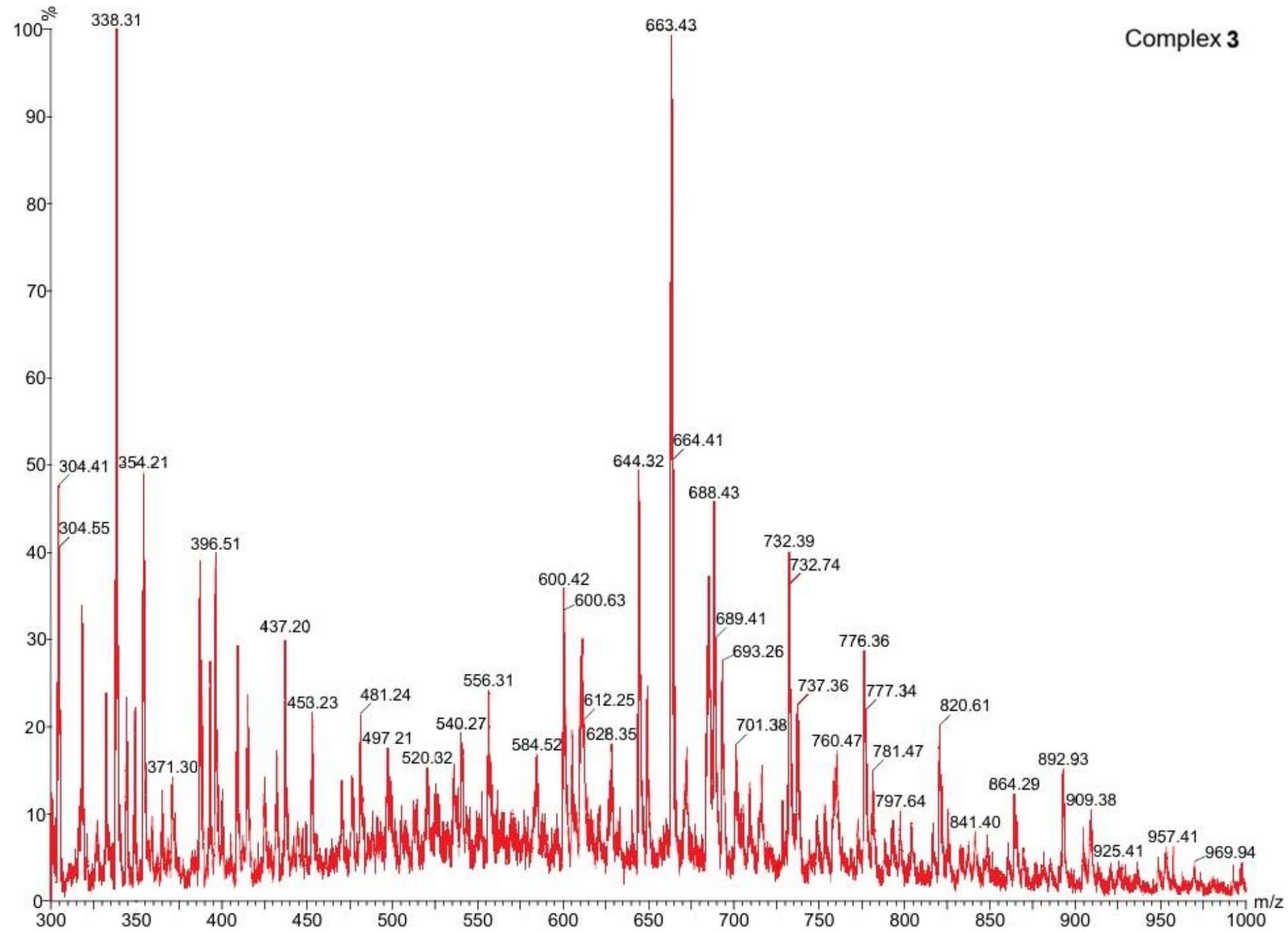

Figure S21. Mass spectrum of complex **3**.

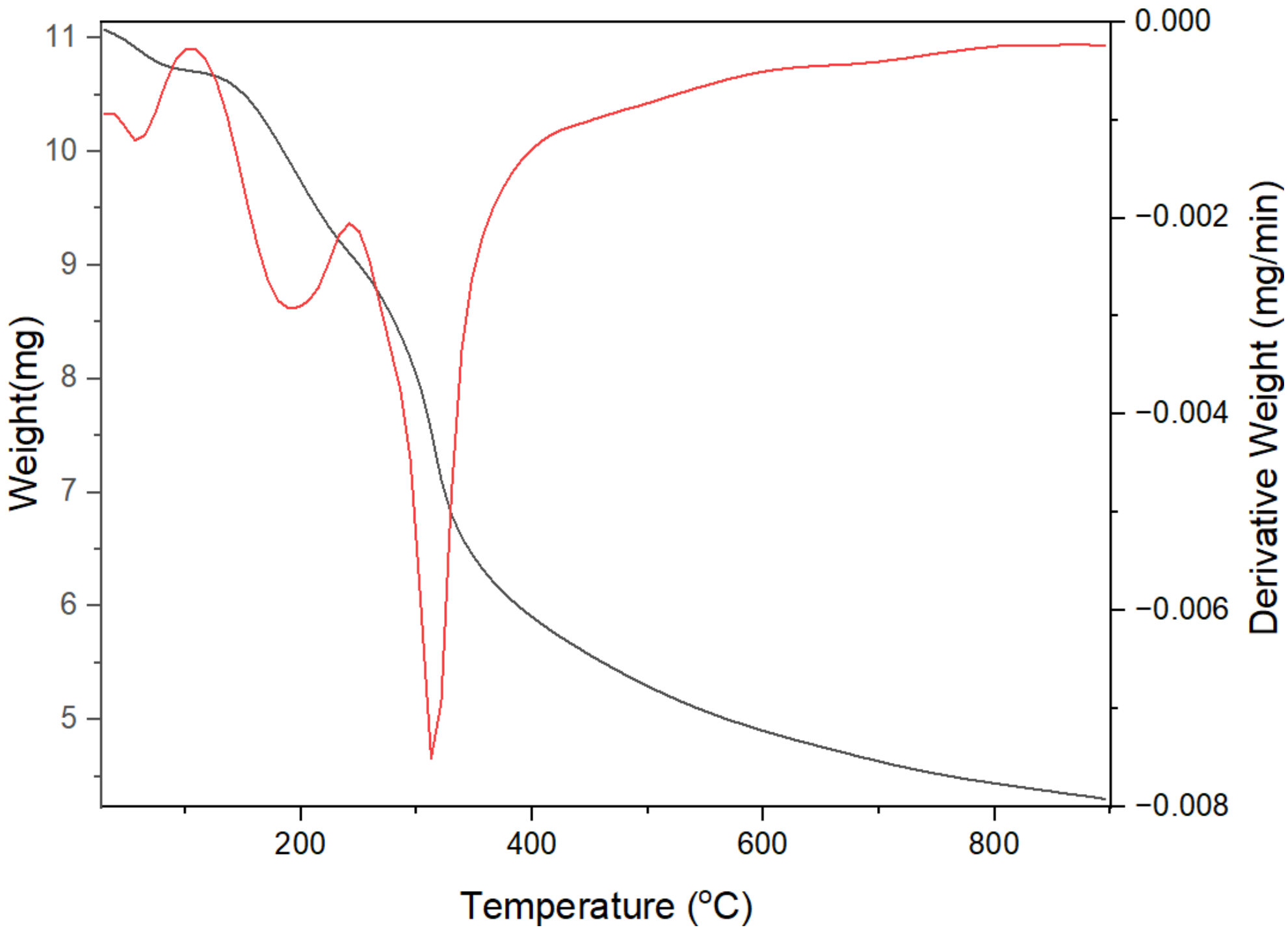

Figure S22. Thermogram of complex **3**.

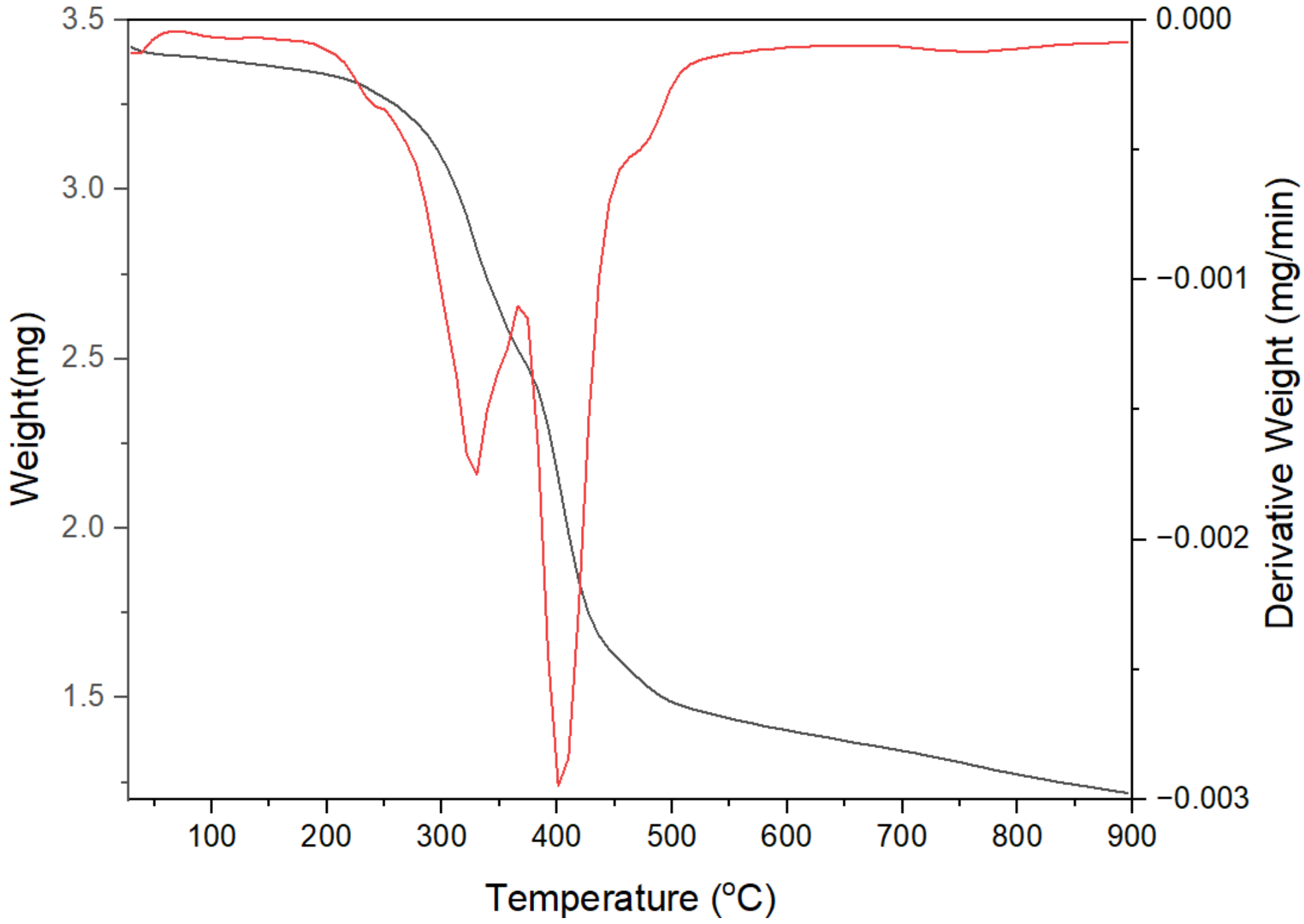

Figure S23. Thermogram of complex **4.**

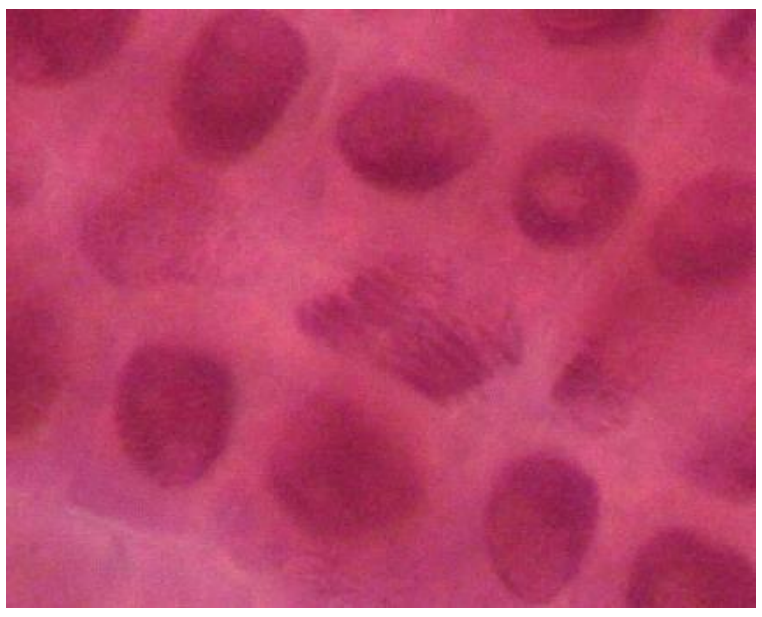

Figure S24. Anaphase in tropokinesis at C_3 concentration of ligand $\mathbf{HL^4}$.

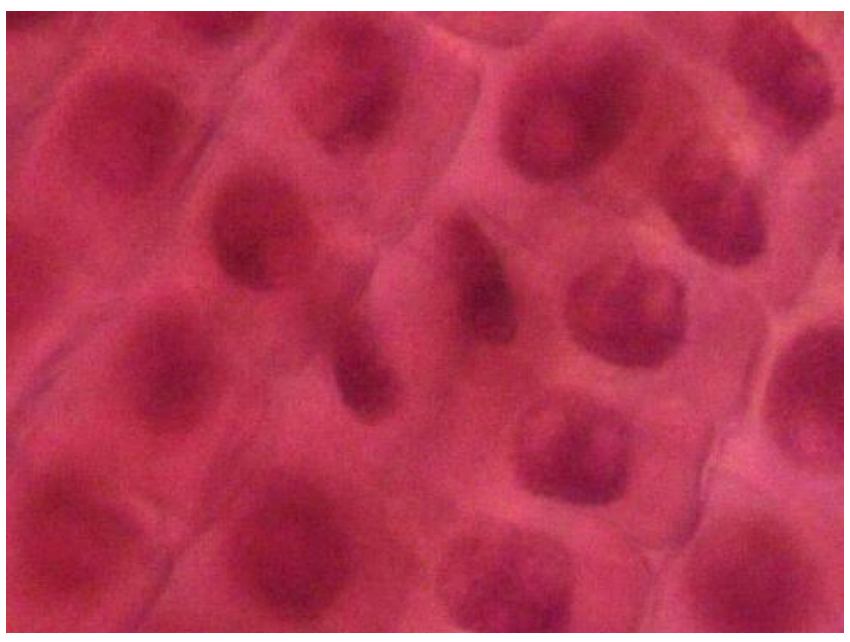

Figure S25. Telophase in tropokinesis at C_5 concentration of ligand $\mathbf{HL^1}$.

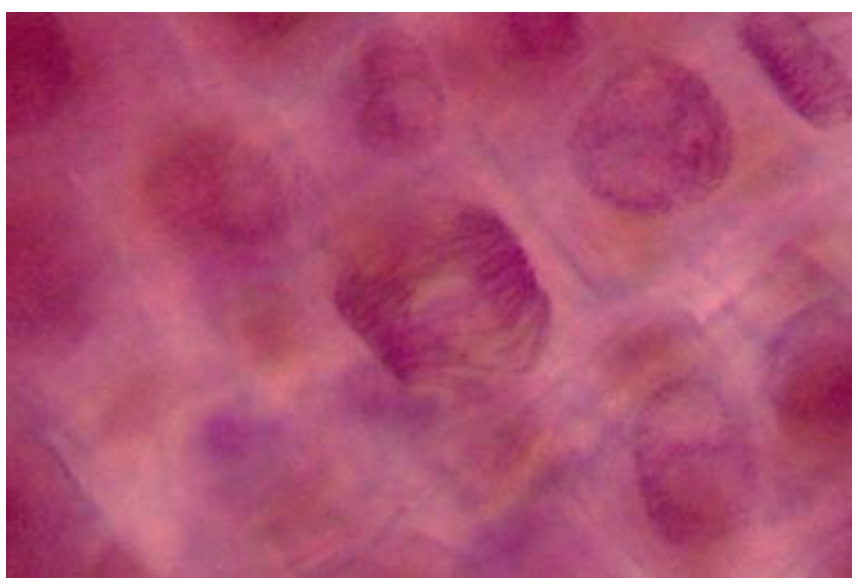

Figure S26. Anaphase with lagging chromosomes at C_1 concentration of ligand $\mathbf{HL^3}$.

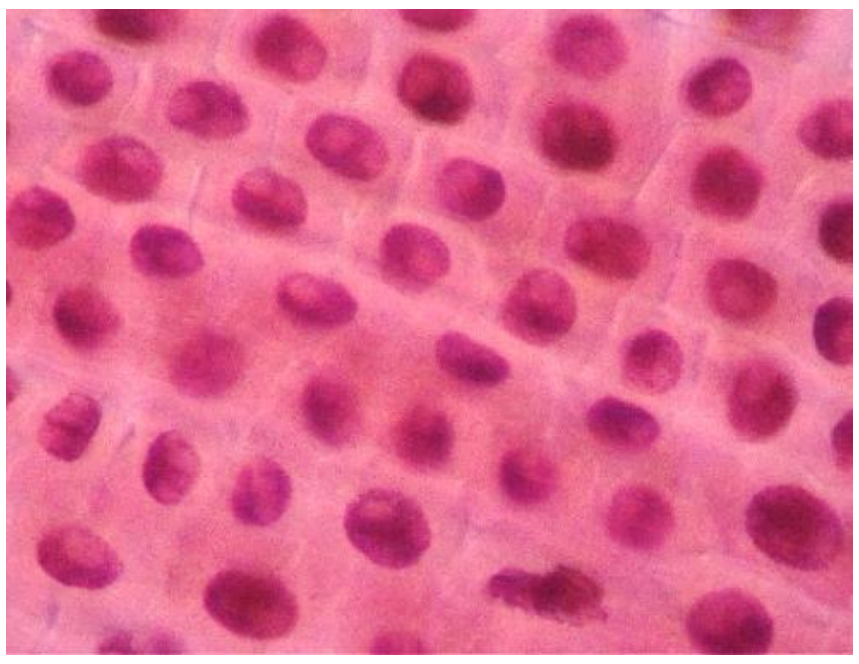

Figure S27. Area with rare cell division phases at C_1 concentration of ligand **HL²**.

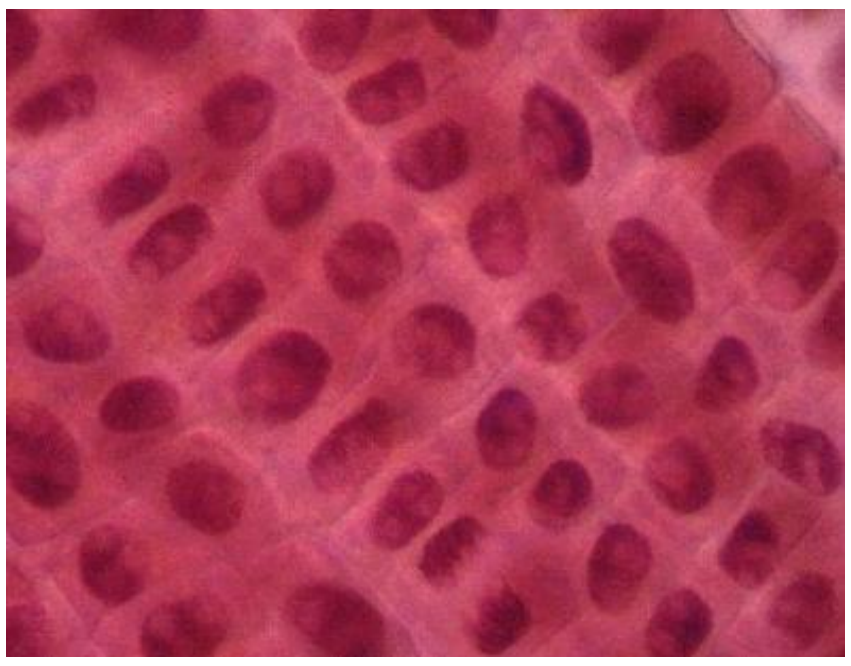

Figure S28. Area with rare cell division phases at C_1 concentration of complex **3**.

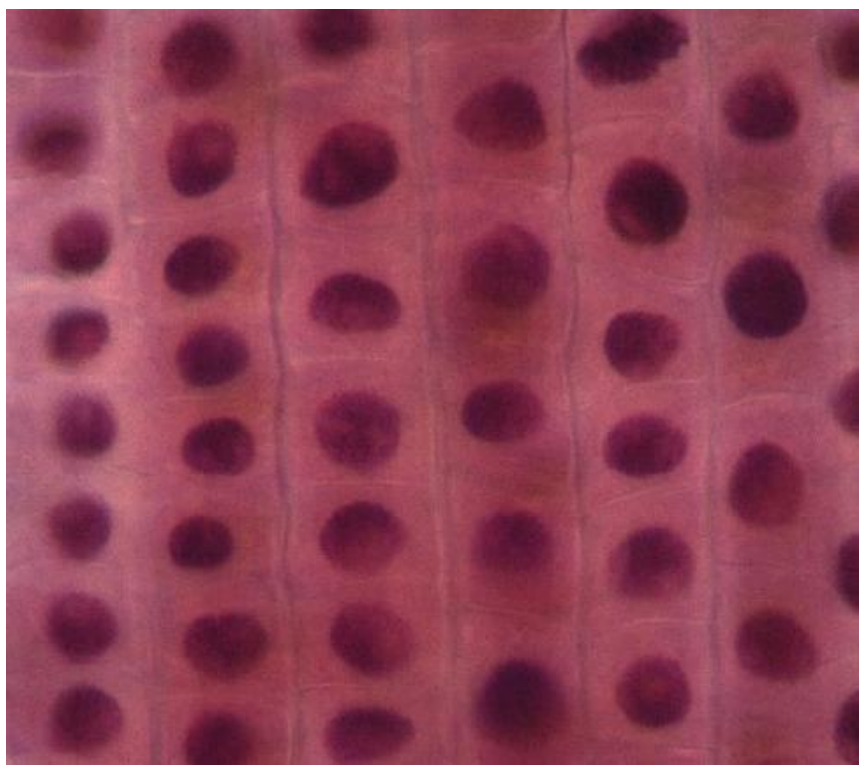

Figure S29. Area with rare cell division phases at C_3 concentration of complex **4**.

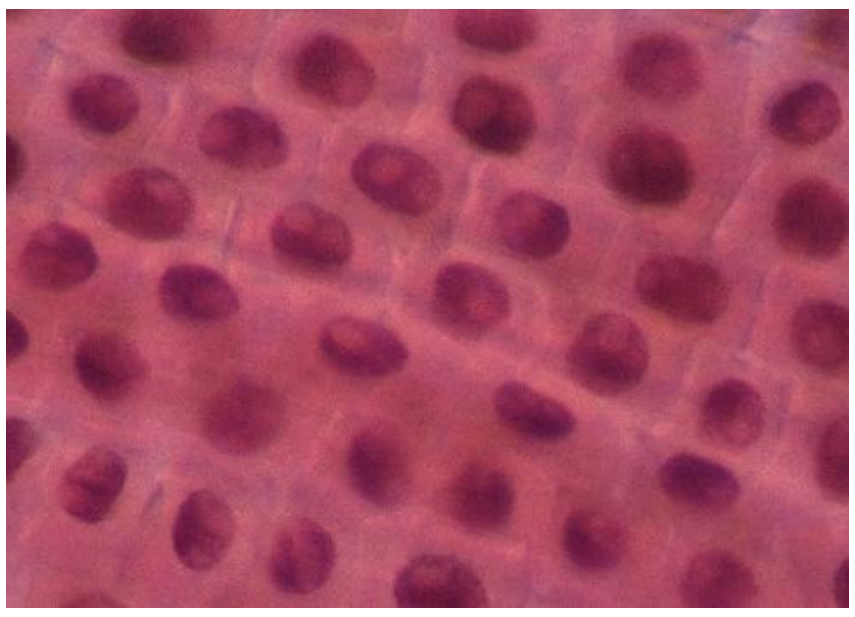

Figure S30. Interphase cells with nuclei showing slight hypertrophied nucleoli at C_1 concentration of ligand **HL²**.

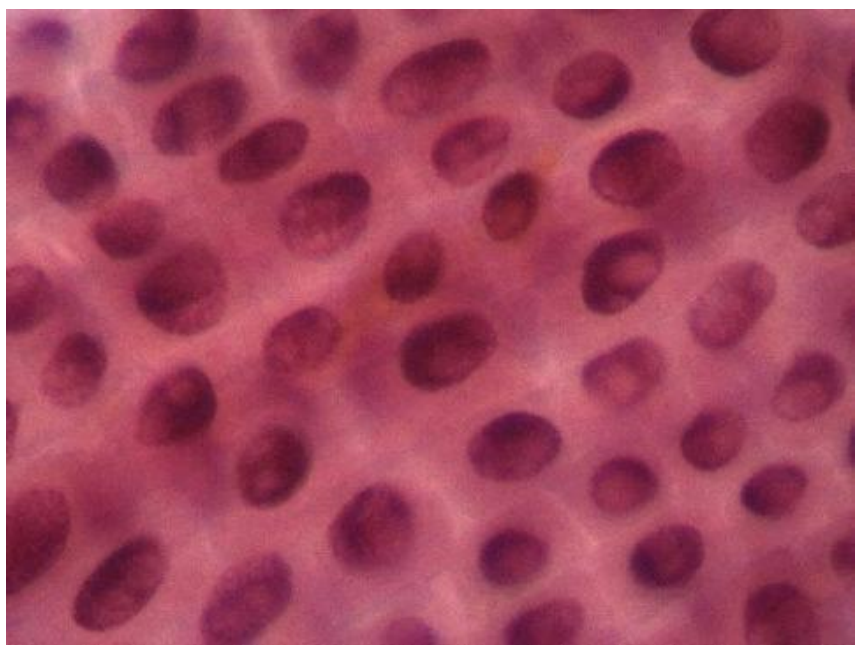

Figure S31. Interphase cells with nuclei showing slight hypertrophied nucleoli at C_1 concentration of complex **3**.

TABLE S1. Bond distances (Å) and angles (°).

HL[1]

**Bonds Lengths (Å)**			**Angles (°)**		
	Molecule			Molecule	
	A	B		A	B
**C1-O1**	1.351(2)	1.350(2)	O1-C1-C2	116.81(17)	117.04(17)
**C1-C2**	1.406(3)	1.401(3)	O1-C1-C6	123.40(17)	123.29(17)
**C1-C6**	1.401(3)	1.409(3)	C6-C1-C2	119.79(17)	119.67(17)
**Cl1-C9**	1.7288(19)	1.7262(19)	C7-N1-C8	121.83(16)	122.64(16)
**F1-C14**	1.316(3)	1.332(2)	O2-C2-C1	120.57(17)	120.64(17)
**N1-C7**	1.289(3)	1.290(2)	O2-C2-C3	119.30(17)	119.22(17)
**N1-C8**	1.408(2)	1.403(2)	C3-C2-C1	120.12(18)	120.13(18)
**F2-C14**	1.336(3)	1.344(2)	C2-C3-C4	120.22(18)	120.51(18)
**C2-O2**	1.364(2)	1.365(2)	C5-C4-C3	120.12(18)	120.00(18)
**C2-C3**	1.384(3)	1.386(3)	C4-C5-C6	120.47(18)	120.48(18)
**F3-C14**	1.296(3)	1.340(2)	C1-C6-C5	119.26(18)	119.21(17)
**C3-C4**	1.398(3)	1.398(3)	C1-C6-C7	121.22(17)	120.86(17)
**C4-C5**	1.381(3)	1.379(3)	C5-C6-C7	119.49(17)	119.91(17)
**C5-C6**	1.408(3)	1.411(3)	N1-C7-C6	121.04(17)	120.83(17)
**C6-C7**	1.450(3)	1.445(3)	C9-C8-N1	117.71(17)	117.19(16)
**C8-C9**	1.407(3)	1.408(3)	C13-C8-N1	125.06(17)	125.38(17)
**C8-C13**	1.396(3)	1.396(3)	C13-C8-C9	117.23(17)	117.44(17)
**C9-C10**	1.386(3)	1.387(3)	C8-C9-Cl1	119.13(15)	119.07(15)
**C10-C11**	1.383(3)	1.385(3)	C10-C9-Cl1	118.91(15)	119.20(15)
**C11-C12**	1.392(3)	1.393(3)	C10-C9-C8	121.95(18)	121.71(17)
**C12-C13**	1.388(3)	1.389(3)	C11-C10-C9	119.86(18)	120.04(18)
**C12-C14**	1.500(3)	1.502(3)	C10-C11-C12	119.15(18)	119.05(18)
			C11-C12-C14	120.35(17)	121.05(17)
			C13-C12-C11	120.98(18)	120.97(18)
			C13-C12-C14	118.67(17)	117.97(17)
			C12-C13-C8	120.83(17)	120.79(17)
			F1-C14-F2	103.07(19)	106.66(16)
			F1-C14-C12	113.24(17)	106.78(16)
			F2-C14-C12	112.08(17)	112.86(16)
			F3-C14-F1	108.8(2)	112.24(16)
			F3-C14-F2	105.8(2)	105.70(16)
			F3-C14-C12	113.16(17)	112.12(16)

HL[2]

**Bonds Lengths (Å)**				**Angles (°)**			
**Cl1-C14**	1.727(4)	C3-C4	1.380(6)	C2-O2-C7	118.6(4)	C14-C9-N1	117.5(3)
**F1-C15**	1.305(5)	C4-C5	1.369(5)	C8-N1-C9	122.1(3)	C11-C10-C9	121.4(3)
**F2-C15**	1.270(6)	C5-C6	1.404(5)	O1-C1-C2	117.6(3)	C10-C11-C12	120.7(4)
**F3-C15**	1.302(6)	C6-C8	1.443(5)	O1-C1-C6	122.9(3)	C10-C11-C15	120.4(4)
**O2-C2**	1.357(4)	C9-C10	1.392(5)	C2-C1-C6	119.5(3)	C12-C11-C15	118.9(3)
**O2-C7**	1.417(5)	C9-C14	1.397(5)	O2-C2-C1	114.6(3)	C13-C12-C11	118.9(3)
**O1-C1**	1.335(4)	C10-C11	1.368(5)	O2-C2-C3	125.8(3)	C14-C13-C12	120.2(3)

**N1-C8**	1.282(4)	C11-C12	1.388(5)	C3-C2-C1	119.5(4)	C9-C14-Cl1	119.2(3)
**N1-C9**	1.404(4)	C11-C15	1.484(6)	C4-C3-C2	120.9(4)	C13-C14-Cl1	119.1(3)
**C1-C2**	1.404(5)	C12-C13	1.381(5)	C5-C4-C3	120.4(4)	C13-C14-C9	121.7(3)
**C1-C6**	1.406(5)	C13-C14	1.374(5)	C4-C5-C6	120.4(4)	F1-C15-C11	114.0(4)
**C2-C3**	1.381(5)	-		C1-C6-C8	120.9(3)	F2-C15-F1	104.4(5)
				C5-C6-C1	119.2(3)	F2-C15-F3	107.1(5)
				C5-C6-C8	119.9(3)	F2-C15-C11	114.8(4)
				N1-C8-C6	121.7(3)	F3-C15-F1	102.5(4)
				C10-C9-N1	125.5(3)	F3-C15-C11	112.9(4)
				C10-C9-C14	117.1(3)	- -	

**HL[3]**							
**Bonds Lengths (Å)**				**Angles (°)**			
**Cl1-C10**	1.734(3)			C8-N1-C9	122.1(2)	C14-C9-N1	124.7(2)
**F1-C15**	1.347(3)			C2-N2-C3	119.3(3)	C14-C9-C10	117.7(2)
**F2-C15**	1.342(3)	C2-C6	1.496(4)	O1-C1-C2	116.7(3)	C9-C10-Cl1	119.5(2)
**F3-C15**	1.340(3)	C3-C4	1.387(4)	O1-C1-C5	123.3(3)	C11-C10-Cl1	118.4(2)
**O1-C1**	1.351(3)	C4-C5	1.407(4)	C5-C1-C2	120.0(3)	C11-C10-C9	122.0(3)
**O2-C7**	1.422(4)	C4-C7	1.503(4)	N2-C2-C1	120.6(3)	C10-C11-C12	119.5(3)
**N1-C8**	1.288(4)	C5-C8	1.467(4)	N2-C2-C6	119.3(3)	C11-C12-C13	119.2(3)
**N1-C9**	1.409(4)	C9-C10	1.403(4)	C1-C2-C6	120.0(3)	C12-C13-C15	121.3(2)
**N2-C2**	1.334(4)	C9-C14	1.392(4)	N2-C3-C4	124.3(3)	C14-C13-C12	121.0(3)
**N2-C3**	1.342(4)	C10-C11	1.389(4)	C3-C4-C5	117.3(3)	C14-C13-C15	117.7(3)
**C1-C2**	1.411(4)	C11-C12	1.390(4)	C3-C4-C7	119.3(3)	C13-C14-C9	120.5(3)
**C1-C5**	1.399(4)	C12-C13	1.393(4)	C5-C4-C7	123.3(3)	F1-C15-C13	112.1(2)
		C13-C14	1.392(4)	C1-C5-C4	118.4(3)	F2-C15-F1	105.4(2)
		C13-C15	1.500(4)	C1-C5-C8	120.8(3)	F2-C15-C13	112.1(2)
				C4-C5-C8	120.8(3)	F3-C15-F1	106.5(2)
				O2-C7-C4	108.6(2)	F3-C15-F2	107.3(2)
				N1-C8-C5	119.1(2)	F3-C15-C13	113.0(2)
				C10-C9-N1	117.6(2)	- -	
**HL[4]**							
**Bonds Lengths (Å)**				**Angles (°)**			
**Cl1-C19**	1.7332(14)	C8-C9	1.4461(18)	N2-N1-C1	119.37(10)	C13-C12-C17	119.96(13)
**F1-C24**	1.3446(17)	C8-C11	1.3834(19)	C7-N1-N2	112.40(11)	C17-C12-C11	118.57(12)
**F2-C24**	1.3375(18)	C9-C10	1.492(2)	C7-N1-C1	128.23(11)	C14-C13-C12	119.84(13)
**F3-C24**	1.3294(18)	C11-C12	1.4875(19)	C9-N2-N1	106.65(11)	C13-C14-C15	120.43(14)
**O1-C7**	1.2518(16)	C12-C13	1.3878(19)	C11-N3-C18	126.89(12)	C16-C15-C14	119.67(15)
**N1-N2**	1.4052(16)	C12-C17	1.3903(19)	C2-C1-N1	120.01(12)	C15-C16-C17	120.29(14)
**N1-C1**	1.4169(18)	C13-C14	1.382(2)	C2-C1-C6	120.66(13)	C16-C17-C12	119.78(14)
**N1-C7**	1.3704(17)	C14-C15	1.389(2)	C6-C1-N1	119.33(13)	C19-C18-N3	119.81(12)
**N2-C9**	1.3074(18)	C15-C16	1.382(2)	C3-C2-C1	119.32(14)	C23-C18-N3	121.49(12)
**N3-C11**	1.3510(17)	C16-C17	1.389(2)	C2-C3-C4	120.45(16)	C23-C18-C19	118.61(12)
**N3-C18**	1.4129(17)	C18-C19	1.4008(19)	C5-C4-C3	119.82(15)	C18-C19-Cl1	120.05(10)
**C1-C2**	1.388(2)	C18-C23	1.3871(19)	C4-C5-C6	120.56(14)	C20-C19-Cl1	118.73(11)
**C1-C6**	1.3918(19)	C19-C20	1.3875(19)	C5-C6-C1	119.17(14)	C20-C19-C18	121.22(13)
**C2-C3**	1.386(2)	C20-C21	1.382(2)	O1-C7-N1	126.34(12)	C21-C20-C19	119.65(13)
**C3-C4**	1.388(2)	C21-C22	1.392(2)	O1-C7-C8	129.12(12)	C20-C21-C22	119.50(12)
**C4-C5**	1.380(2)	C22-C23	1.3882(19)	N1-C7-C8	104.53(11)	C21-C22-C24	119.94(12)
**C5-C6**	1.389(2)	C22-C24	1.493(2)	C9-C8-C7	105.24(11)	C23-C22-C21	120.86(13)
**C7-C8**	1.4509(19)	-		C11-C8-C7	122.17(12)	C23-C22-C24	119.20(13)
				C11-C8-C9	132.56(13)	C18-C23-C22	120.06(13)
				N2-C9-C8	111.14(12)	F1-C24-C22	111.82(13)
				N2-C9-C10	119.26(12)	F2-C24-F1	105.55(12)
				C8-C9-C10	129.58(12)	F2-C24-C22	112.46(12)
				N3-C11-C8	117.71(12)	F3-C24-F1	106.57(12)
				N3-C11-C12	119.63(11)	F3-C24-F2	106.72(13)
				C8-C11-C12	122.58(12)	F3-C24-C22	113.20(12)
				C13-C12-C11	121.46(12)	- -	

Complex 2

**Bonds Lengths (Å)**				**Angles (°)**			
**Pd1-Pd2**	2.8441(16)	C11-C12	1.3900	O1-Pd1-Pd2	96.3(3)	F2-C15-C13	110(2)
**Pd1-O1**	1.967(9)	C11-C10	1.3900	O1-Pd1-O6	175.1(4)	F3-C15-C13	111(2)
**Pd1-O6**	2.040(8)	C12-C13	1.3900	O1-Pd1-O8	88.6(4)	O5-C16-O6	127.9(13)
**Pd1-O8**	2.028(11)	C13-C14	1.3900	O1-Pd1-N1	92.1(4)	O5-C16-C17	116.3(14)
**Pd1-N1**	1.990(12)	C13-C15	1.53(2)	O6-Pd1-Pd2	82.3(3)	C14-C13-C15	118.9(15)
**Pd2-O3**	1.974(10)	C14-C9	1.3900	O8-Pd1-Pd2	80.2(3)	C13-C14-C9	120.0
**Pd2-O5**	2.054(10)	C9-C10	1.3900	O8-Pd1-O6	86.6(4)	C14-C9-N1	120.4(15)
**Pd2-O7**	2.033(11)	C16-C17	1.520(16)	N1-Pd1-Pd2	102.5(3)	C14-C9-C10	120.0
**Pd2-N2**	1.975(11)	C18-C19	1.54(2)	N1-Pd1-O6	92.8(4)	C10-C9-N1	119.3(15)
**Cl1-C10**	1.672(12)	C23-C22	1.3900	N1-Pd1-O8	177.1(4)	C11-C10-Cl1	116(2)
**Cl2-C29**	1.705(10)	C23-C24	1.3900	O3-Pd2-Pd1	97.0(3)	C9-C10-Cl1	124(2)
**F1-C15**	1.271(15)	C22-C21	1.3900	O3-Pd2-O5	88.0(4)	C9-C10-C11	120.0
**F2-C15**	1.271(15)	C21-C20	1.3900	O3-Pd2-O7	175.0(4)	F1-C15-F2	106.3(18)
**F3-C15**	1.272(15)	C20-C25	1.3900	O3-Pd2-N2	92.3(5)	F1-C15-F3	104.9(17)
**F4-C34**	1.326(12)	C25-C24	1.3900	O5-Pd2-Pd1	80.5(3)	F1-C15-C13	118(3)
**F5-C34**	1.323(12)	C25-C27	1.443(18)	O7-Pd2-Pd1	82.5(3)	F2-C15-F3	105.6(18)
**F6-C34**	1.331(13)	C28-C33	1.3900	O7-Pd2-O5	87.0(4)	O6-C16-C17	115.8(14)
**O1-C1**	1.319(11)	C28-C29	1.3900	N2-Pd2-Pd1	101.5(3)	O7-C18-C19	115.6(16)
**O2-C2**	1.376(14)	C33-C32	1.3900	N2-Pd2-O5	177.9(4)	O8-C18-O7	128.9(17)
**O2-C7**	1.408(16)	C32-C31	1.3900	N2-Pd2-O7	92.8(5)	O8-C18-C19	115.5(17)
**O3-C20**	1.310(12)	C32-C34	1.54(2)	C1-O1-Pd1	124.5(8)	C22-C23-C24	120.0
**O4-C21**	1.347(16)	C31-C30	1.3900	C2-O2-C7	117.7(13)	C21-C22-C23	120.0
**O4-C26**	1.37(2)	C30-C29	1.3900	C20-O3-Pd2	125.5(9)	O4-C21-C22	123.3(13)
**O5-C16**	1.248(16)			C21-O4-C26	118.7(17)	O4-C21-C20	116.6(13)
**O6-C16**	1.270(16)			C16-O5-Pd2	124.5(10)	C22-C21-C20	120.0
**O7-C18**	1.256(19)			C16-O6-Pd1	122.6(9)	O3-C20-C21	116.3(12)
**O8-C18**	1.246(17)			C18-O7-Pd2	121.7(11)	O3-C20-C25	123.7(12)
**N1-C8**	1.266(14)			C18-O8-Pd1	124.6(11)	C21-C20-C25	120.0
**N1-C9**	1.419(13)			C8-N1-Pd1	125.9(10)	C20-C25-C27	124.9(12)
**N2-C27**	1.287(17)			C8-N1-C9	122.8(16)	C24-C25-C20	120.0
**N2-C28**	1.431(13)			C9-N1-Pd1	111.2(12)	C24-C25-C27	115.1(13)
**C4-C5**	1.3900			C9X-N1-Pd1	120.6(16)	C25-C24-C23	120.0
**C4-C3**	1.3900			C27-N2-Pd2	124.0(11)	N2-C27-C25	126.1(15)
**C5-C6**	1.3900			C27-N2-C28	116.4(12)	C33-C28-N2	120.4(10)
**C6-C1**	1.3900			C28-N2-Pd2	119.5(8)	C33-C28-C29	120.0
**C6-C8**	1.420(15)			C5-C4-C3	120.0	C29-C28-N2	119.1(10)
**C1-C2**	1.3900			C4-C5-C6	120.0	C28-C33-C32	120.0
**C2-C3**	1.3900			C5-C6-C8	114.3(11)	C33-C32-C31	120.0
		-		C1-C6-C5	120.0	C33-C32-C34	118.6(11)
				C1-C6-C8	125.4(11)	C31-C32-C34	121.4(11)
				O1-C1-C6	124.4(11)	C32-C31-C30	120.0
				O1-C1-C2	115.6(11)	C29-C30-C31	120.0
				C2-C1-C6	120.0	C28-C29-Cl2	119.7(9)
				O2-C2-C1	117.4(11)	C30-C29-Cl2	120.0(9)
				O2-C2-C3	122.6(11)	C30-C29-C28	120.0
				C1-C2-C3	120.0	F4-C34-F6	109.0(14)
				C2-C3-C4	120.0	F4-C34-C32	107.7(18)
				N1-C8-C6	125.3(14)	F5-C34-F4	109.3(14)
				C12-C11-C10	120.0	F5-C34-F6	108.7(14)
				C11-C12-C13	120.0	F5-C34-C32	111.9(18)
				C12-C13-C15	120.6(15)	F6-C34-C32	110.2(18)
				C14-C13-C12	120.0		

Complex 4

**Bonds Lengths (Å)**				**Angles (°)**			
**Pd1-O1**	2.002(3)	C9-C10	1.503(6)	O1-Pd1-O1[1]	180.0	C8-C11-C12	118.3(3)
**Pd1-O1[1]**	2.002(3)	C11-C12	1.501(5)	O1[1]-Pd1-N1	87.09(12)	C13-C12-C11	121.6(4)
**Pd1-N1**	2.052(3)	C12-C13	1.383(6)	O1-Pd1-N1	92.91(12)	C13-C12-C17	120.0(4)
**Pd1-N1[1]**	2.052(3)	C12-C17	1.392(6)	O1[1]-Pd1-N1[1]	92.91(12)	C17-C12-C11	118.3(4)

**Cl1-C19**	1.729(4)	C13-C14	1.397(6)	O1-Pd1-N1^{1}	87.09(12)	C12-C13-C14	119.3(4)
**O1-C7**	1.286(5)	C14-C15	1.382(7)	N1-Pd1-N1^{1}	180.00(16)	C15-C14-C13	120.2(4)
**N1-C11**	1.315(5)	C15-C16	1.377(7)	C7-O1-Pd1	119.6(2)	C16-C15-C14	120.6(4)
**N1-C18**	1.439(5)	C16-C17	1.382(6)	C11-N1-Pd1	125.5(3)	C15-C16-C17	119.6(4)
**N2-N3**	1.401(5)	C18-C19	1.387(6)	C11-N1-C18	117.0(3)	C16-C17-C12	120.4(4)
**N2-C9**	1.310(5)	C18-C23	1.390(6)	C18-N1-Pd1	117.3(2)	C19-C18-N1	121.5(4)
**N3-C1**	1.425(5)	C19-C20	1.386(6)	C9-N2-N3	105.5(3)	C19-C18-C23	119.0(4)
**N3-C7**	1.349(5)	C20-C21	1.373(6)	N2-N3-C1	118.5(3)	C23-C18-N1	119.5(4)
**C1-C2**	1.385(6)	C21-C22	1.386(6)	C7-N3-N2	111.9(3)	C18-C19-Cl1	120.2(3)
**C1-C6**	1.385(6)	C22-C23	1.394(6)	C7-N3-C1	129.0(3)	C20-C19-Cl1	119.1(3)
**C2-C3**	1.389(7)	C22-C24	1.484(6)	C2-C1-N3	118.9(4)	C20-C19-C18	120.7(4)
**C3-C4**	1.379(8)	C24-F1	1.311(8)	C6-C1-N3	119.7(4)	C21-C20-C19	120.2(4)
**C4-C5**	1.378(8)	C24-F2	1.377(8)	C6-C1-C2	121.3(4)	C20-C21-C22	119.9(4)
**C5-C6**	1.399(6)	C24-F3	1.302(7)	C1-C2-C3	118.7(5)	C21-C22-C23	120.0(4)
**C7-C8**	1.408(5)			C4-C3-C2	120.5(5)	C21-C22-C24	119.8(4)
**C8-C9**	1.442(5)			C5-C4-C3	120.8(5)	C23-C22-C24	120.2(4)
**C8-C11**	1.411(5)			C4-C5-C6	119.5(5)	C18-C23-C22	120.2(4)
				C1-C6-C5	119.3(5)	F1-C24-C22	115.0(4)
				O1-C7-N3	121.3(3)	F1-C24-F2	103.5(5)
				O1-C7-C8	132.0(4)	F2-C24-C22	111.2(4)
				N3-C7-C8	106.7(3)	F3-C24-C22	114.9(4)
				C7-C8-C9	104.5(3)	F3-C24-F1	107.2(5)
				C7-C8-C11	124.6(3)	F3-C24-F2	103.9(5)
				C11-C8-C9	130.7(4)		
				N2-C9-C8	111.5(4)		
				N2-C9-C10	118.1(4)		
				C8-C9-C10	130.4(4)		
				N1-C11-C8	122.4(3)		
				N1-C11-C12	119.1(3)		

Symmetry code: ^{1}1-*x*,1-*y*,1-*z*

TABLE S2. Mass spectral data of complexes **1** and **3**.

**Molecular formula**	**Mw (g/mol)**	**Molecular ion peak [M]$^+$**	**The peaks due to complex fragmentation**			
**[Pd(L^1)$_2$] (1)**	735.75	732.46	556.38	600.28	663.43	688.43
**[Pd(L^3)$_2$] (3)**	793.83	781.47	481.24	644.32	737.36	776.36
